# 5-Nitro-1,2-benzothiazol-3-amine and *N*-Ethyl-1-[(ethylcarbamoyl)(5-nitro-1,2-benzothiazol-3-yl)amino]formamide Modulate α-Synuclein and Tau Aggregation

**DOI:** 10.1021/acsomega.3c02668

**Published:** 2023-05-23

**Authors:** Eduardo Ramirez, Susantha K. Ganegamage, Ahmed A. Elbatrawy, Heba Alnakhala, Kazuma Shimanaka, Arati Tripathi, Sehong Min, Jean-Christophe Rochet, Ulf Dettmer, Jessica S. Fortin

**Affiliations:** † Department of Basic Medical Sciences, College of Veterinary Medicine, Purdue University, 625 Harrison Street, West Lafayette, Indiana 47907, United States; ‡ Department of Neurology, Brigham and Women’s Hospital and Harvard Medical School, Ann Romney Center for Neurologic Diseases, Boston, Massachusetts 02115, United States; § Department of Medicinal Chemistry and Molecular Pharmacology, College of Pharmacy, Purdue University, West Lafayette, Indiana 47906, United States

## Abstract

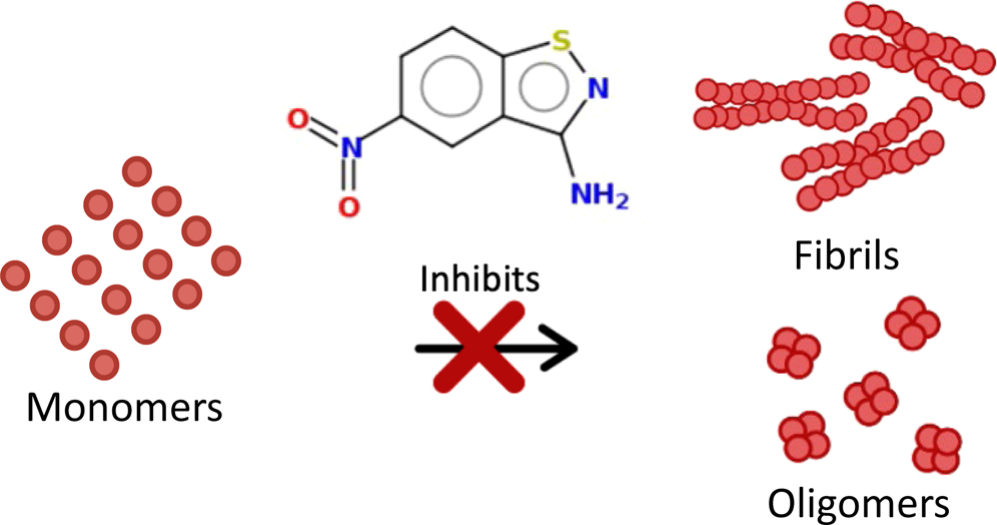

Protein misfolding results in a plethora of known diseases such as Alzheimer’s disease, Parkinson’s disease, Huntington’s disease, transthyretin-related amyloidosis, type 2 diabetes, Lewy body dementia, and spongiform encephalopathy. To provide a diverse portfolio of therapeutic small molecules with the ability to reduce protein misfolding, we evaluated a set of 13 compounds: 4-(benzo[*d*]thiazol-2-yl)aniline (BTA) and its derivatives containing urea (**1**), thiourea (**2**), sulfonamide (**3**), triazole (**4**), and triazine (**5**) linker. In addition, we explored small modifications on a very potent antioligomer 5-nitro-1,2-benzothiazol-3-amine (5-NBA) (compounds **6–13**). This study aims to define the activity of BTA and its derivatives on a variety of prone-to-aggregate proteins such as transthyretin (TTR_81–127_, TTR_101–125_), α-synuclein (α-syn), and tau isoform 2N4R (tau 2N4R) through various biophysical methods. Thioflavin T (ThT) fluorescence assay was used to monitor fibril formation of the previously mentioned proteins after treatment with BTA and its derivatives. Antifibrillary activity was confirmed using transmission electron microscopy (TEM). Photoreactive cross-linking assay (PICUP) was utilized to detect antioligomer activity and lead to the identification of 5-NBA (at low micromolar concentration) and compound **13** (at high concentration) as the most promising in reducing oligomerization. 5-NBA and not BTA inhibited the inclusion formation based on the cell-based assay using M17D neuroblastoma cells that express inclusion-prone αS-3K::YFP. 5-NBA abrogated the fibril, oligomer, and inclusion formation in a dose-dependent manner. 5-NBA derivatives could be the key to mitigate protein aggregation. In the future, the results made from this study will provide an initial platform to generate more potent inhibitors of α-syn and tau 2N4R oligomer and fibril formation.

## Introduction

Proteins are large macromolecules comprised of long chains of amino acids that play various functional roles throughout the body and whose structure is important in order to fulfill that role. When the native structure of the protein is altered, the protein can become nonfunctional and, in some cases, detrimental to the cell. Diseases that form from such distorted proteins are known as protein folding disorders.^1^ There are at least 42 different proteins that have been identified with high propensity to change conformation and form fibrils that accumulate into extracellular amyloid-like deposits.^2,3^ Fibril formation and buildup into extracellular amyloid deposits have been associated with a long list of serious chronic diseases such as AA amyloidosis, Alzheimer’s disease (AD), monoclonal immunoglobulin light-chain amyloidosis, Huntington’s disease, Parkinson’s disease (PD), prion disorders, amyotrophic lateral sclerosis, type 2 diabetes, and transthyretin-related amyloidosis.^4,5^ In each disease, different endogenous proteins self-assemble into highly ordered fibrillar structures. Although there is no specific sequence homology between these proteins, they all undergo major conformational changes to produce β-sheet structures that strongly tend to aggregate into water-insoluble fibrous polymers.^6,7^ Individual misfolded protein monomers conjoin to form oligomers, which elongate to form amyloid fibrils, which will then accumulate extracellularly into deposits during the final state of this process, known formally as amyloidosis.^8^ Short fibrils and intermediate species, such as oligomers, have been shown to be toxic to cells even more than the fibrils, causing membrane leakage, oxidative stress, and activation of caspases 9 and 3.^8^ Therefore, it is crucial to find therapeutic strategies to mitigate the formation of oligomers.

Much effort has been directed toward understanding of the molecular mechanism of amyloid depositions. One of the most well-known protein misfolding diseases is the neurodegenerative condition known as AD. AD is associated with the formation and accumulation of amyloid-β (Aβ) in the brain as well as tangle formation due to misfolding of the tubulin associate unit (tau) protein.^9−23^ Another important misfolding protein, α-synuclein (α-syn), is highly involved in the pathophysiology of PD. When α-syn misfolds, it aggregates together and forms inclusions within the neurons called Lewy bodies. Lewy bodies lead to cell lysis and may spread to other neurons via the synaptic cleft.^24−28^ Transthyretin (TTR) is another protein involved in neurodegenerative diseases such as familial amyloid polyneuropathy and transthyretin amyloid cardiomyopathy.^29,30^ This protein is mostly formed in the liver and plays a key role in the progression of amyloid fibrils after the dissociation of the TTR tetramer into monomers, which then unfold into oligomers.^31^ Currently, there is a critical need to develop additional therapeutics for resolving neurodegenerative diseases associated with protein disorders or halting their progression.

**Figure 1:**
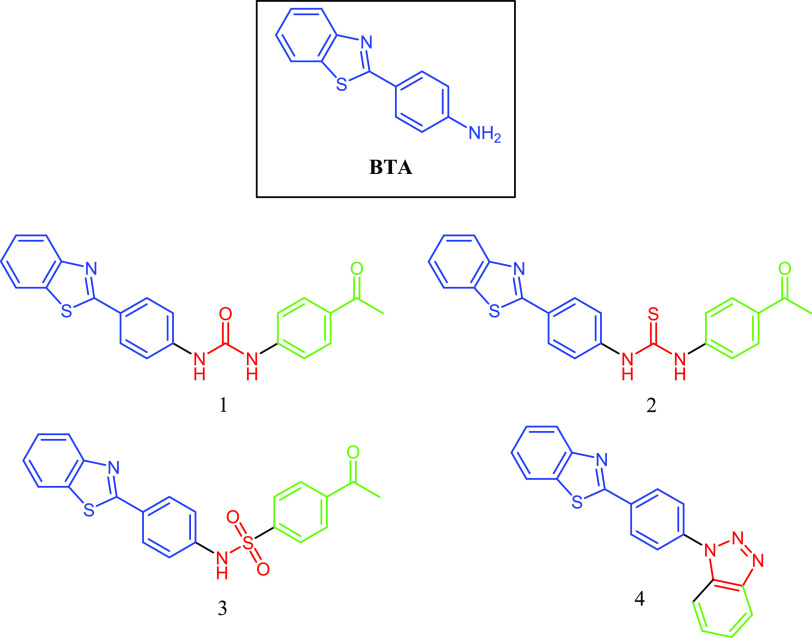
Representative structures of 4-(benzo[*d*]thiazol-2-yl)aniline (BTA) and its derivatives: urea (**1**), thiourea (**2**), sulfonamide (**3**), and triazole (**4**). The original compound is designated in blue, and the derivative structural modifications are indicated in red and green.

One common therapeutic approach to dealing with misfolded proteins is the use of small molecules as stabilizers. Benzothiazoles and their derivatives represent a privileged scaffold, commonly found in several natural products and pharmaceutic agents.^32,33^ For instance, a 2-(4-aminophenyl)benzothiazole (BTA)-based compound, Pittsburgh B, has been used for decades as an amyloid-binding diagnostic agent in Alzheimer’s disease.^34^ BTA-3, another benzothiazole-based compound, was employed to study the specificity of optical probes to the binding sites in amyloid fibrils.^35^ BTA is also present in thioflavin T (ThT), an important diagnostic tool for detecting amyloidosis in histological sections or monitoring the kinetics of fibril formation in vitro.^36^ Based on proven antiaggregation effects in the past, we aimed to study the effect of 4-(benzo[*d*]thiazol-2-yl)aniline (BTA) on common prone-to-aggregate proteins to assess its potential as a starting point in designing more potent molecules. BTA derivatives containing urea (**1**), thiourea (**2**), sulfonamide (**3**), triazole (**4**) (Figure 1), and triazine (**5**) (Table 1) were synthesized. Additionally, small modifications on a very potent antioligomer 5-nitro-1,2-benzothiazol-3-amine (5-NBA) were explored (**6–13**). With this background, we herein explored the antifibrillary effect of all compounds on α-syn and transthyretin (TTR_81–127_). BTA and 5-NBA were further tested on islet amyloid polypeptide (IAPP), α-syn, and transthyretin (TTR_81–127_, TTR_101–125_) using ThT fluorescence assays. The antifibrillary and antioligomer effects of the best compound were further explored with α-syn and tau isoform 2N4R by transmission electron microscopy (TEM) and photoinduced cross-linking of unmodified proteins (PICUP), respectively. These biophysical methods coupled with cell-based assays will determine whether BTA and its derivatives hold potential to inhibit oligomer and fibril formation.

**Table 1:**
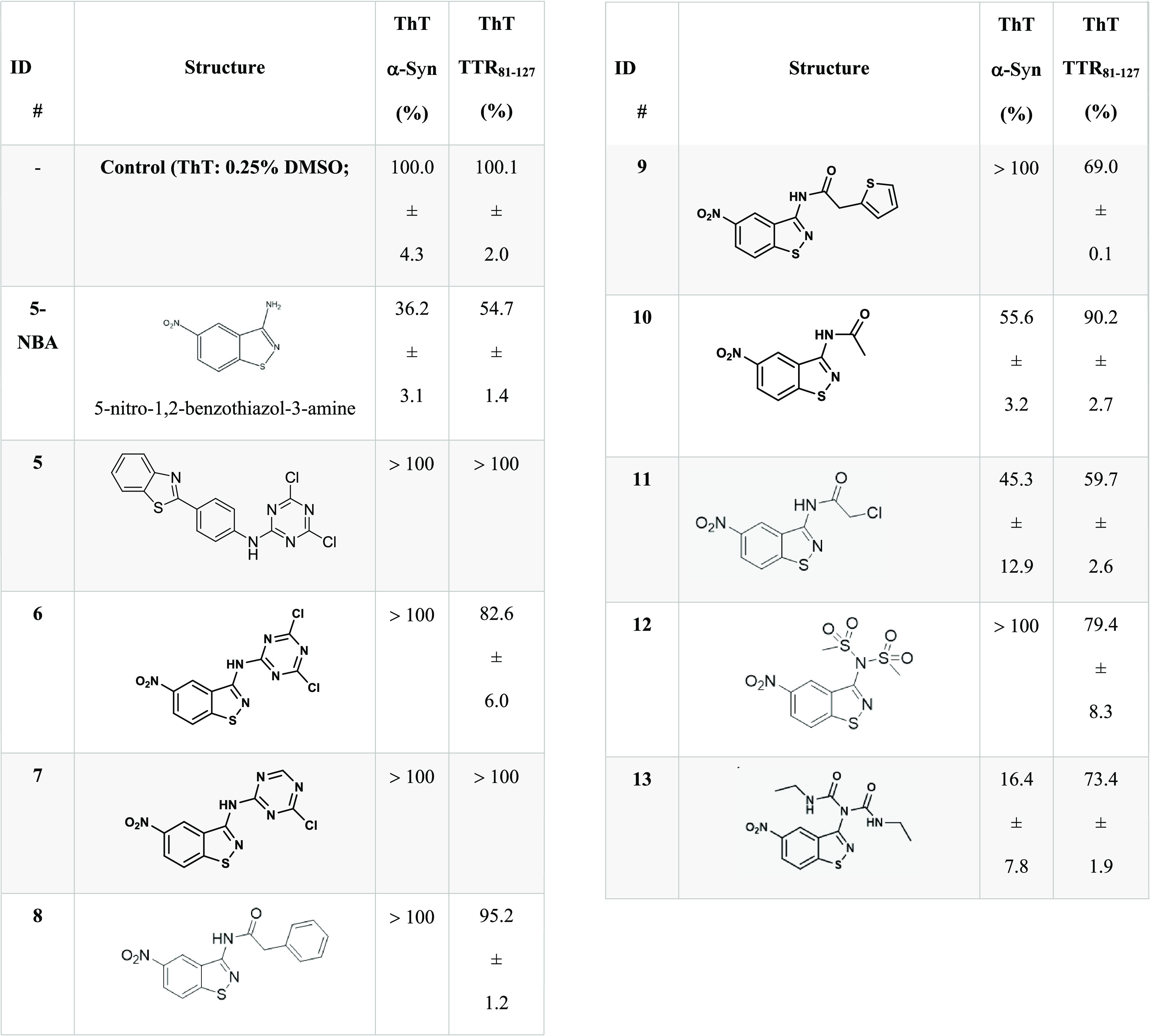
Molecular Structures of Novel Benzothiazole-Linked Derivatives and Their Respective Antifibrillary Activity on α-Synuclein (α-Syn, 2 μM Final Concentration) and Transthyretin Fragment Peptide (TTR_81–127_, 10 μM Final Concentration) Expressed as Maximum Thioflavin T (ThT) Intensity in Percentage in Which the Compounds Were Tested at 100 μM^a^

a Data represent the average of three replicates with SEM.

## Experimental Section

### Chemical Synthesis: General Considerations

All moisture-sensitive reactions were conducted in oven-dried glassware under an atmosphere of dry nitrogen. Reaction solvents (CH_2_Cl_2_, Et_2_O, THF, DMF) were purchased from Sigma-Aldrich in anhydrous form. All other solvents and reagents were purchased from commercial suppliers and used as received unless otherwise specified. Thin-layer chromatography (TLC) was performed with glass plates precoated with silica 60 Å F254 (250 mm) and visualized by UV light. ^1^H and ^13^C NMR spectra were recorded using a 500 MHz Bruker instrument working at a frequency of 500 MHz for ^1^H and at 126 MHz for ^13^C. Chemical shifts are reported in ppm using residual solvent resonances as internal reference (d 7.26 and d 77.0 for ^1^H and ^13^C in CDCl_3_, DMSO-*d*_6_ 2.50 and DMSO-*d*_6_ 39.51 for ^1^H and ^13^C in DMSO-*d*_6_). ^1^H NMR data are reported as follows: b = broad, s = singlet, d = doublet, t = triplet, q = quartet, m = multiplet. Coupling constants are given in hertz. The purity of all compounds and synthetic intermediates was judged to be 95% or better based on ^1^H NMR. IR measurements were performed in a Nicolet FTIR as thin films. High-resolution mass spectrometry analyses were conducted at the Purdue University Bentley and/or Chemistry Mass Spectrometry facility.

### 1-(4-Acetylphenyl)-3-[4-(1,3-benzothiazol-2-yl)phenyl]urea (**1**)

To a stirred solution of 4-(benzo[*d*]thiazol-2-yl)aniline (1 equiv) in anhydrous dichloromethane (10 mL) under a nitrogen atmosphere was added 4-acetylphenyl isocyanate (1.1 equiv) dropwise. The reaction was stirred at room temperature for 24 h until a precipitate was formed. On completion of the reaction monitored by TLC, the precipitate was filtered, washed thrice with ether, and dried *in vacuo* to obtain the desired product (compound **1**, 122 mg) with a yield of 71%. ^1^H NMR (500 MHz, DMSO) δ 9.21 (d, *J* = 4.9 Hz, 2H), 8.19–7.83 (m, 6H), 7.72–7.31 (m, 6H), 2.51 (s, 3H). ^13^C NMR (126 MHz, DMSO) δ 196.8, 167.5, 154.1, 152.4, 144.5, 142.8, 134.7, 131.1, 130.1, 128.6, 127.0, 127.0, 125.6, 123.0, 122.7, 118.9, 117.8, 26.8. HRMS-ESI (*m*/*z*): [M + H]^+^ calcd for C_22_H_17_N_3_O_2_S, 388.1119, found [M + H]^+^ 388.1123.

### 1-(4-Acetylphenyl)-3-[4-(1,3-benzothiazol-2-yl)phenyl]thiourea (**2**)

To a stirred solution of 4-(benzo[*d*]thiazol-2-yl)aniline (1 equiv) in anhydrous tetrahydrofurane (30 mL) under a nitrogen atmosphere was added 4-acetylphenyl isothiocyanate (1.1 equiv) dropwise. The reaction was refluxed for 24 h. On completion of the reaction monitored by TLC, the precipitate was filtered, washed thrice with ether, and dried *in vacuo* to obtain the desired product (compound **2**, 135 mg) with a yield of 76%. ^1^H NMR (500 MHz, DMSO) δ 10.36 (d, *J* = 13.3 Hz, 2H), 8.25–7.86 (m, 6H), 7.83–7.30 (m, 6H), 2.53 (s, 3H). ^13^C NMR (126 MHz, DMSO) δ 197.1, 179.5, 167.3, 154.1, 144.4, 142.7, 134.8, 132.8, 129.4, 129.0, 128.1, 127.1, 125.8, 123.6, 123.1, 122.8, 122.3, 27.0. HRMS-ESI (*m*/*z*): [M + Na]^+^ calcd for C_22_H_17_N_3_OS_2_, 426.0711, found [M + Na]^+^ 426.0711.

### 4-Acetyl-*N*-[4-(1,3-benzothiazol-2-yl)phenyl]benzene-1-sulfonamide (**3**)

To a solution of 2-(4-aminophenyl)benzothiazole (200 mg, 0.88 mmol) in pyridine (2 mL) was added 4-acetylbenzenesulfonyl chloride (1.5 equiv). The reaction mixture was heated under reflux for 10 min and then was cooled, and water (5 mL) was added. The precipitate formed was collected by filtration, washed with water, and dried *in vacuo* to obtain the desired product (compound **3**, 355 mg) with a yield of 91%. ^1^H NMR (500 MHz, DMSO) δ 10.94 (s, 1H), 8.14–8.04 (m, 3H), 8.03–7.89 (m, 5H), 7.49 (m, *J* = 8.3, 7.2, 1.3 Hz, 1H), 7.41 (m, *J* = 8.3, 7.2, 1.2 Hz, 1H), 7.35–7.19 (m, 2H), 2.56 (s, 3H). ^13^C NMR (126 MHz, DMSO) δ 197.7, 167.0, 154.0, 143.3, 140.7, 140.4, 134.8, 129.7, 129.0, 127.6, 127.1, 125.9, 123.1, 122.8, 120.1, 27.5. HRMS-ESI (*m*/*z*): [M + H]^+^ calcd for C_21_H_16_N_2_O_3_S_2_, 409.0680, found [M + H]^+^ 409.0702.

### 1-[4-(1,3-Benzothiazol-2-yl)phenyl]-1*H*-1,2,3-benzotriazole (**4**)

Compound 4 was synthesized using the procedure reported as published previously.^37^ Amount: 128 mg, 88%. ^1^H NMR (500 MHz, DMSO) δ 8.37 (d, *J* = 8.3 Hz, 2H), 8.20 (dd, *J* = 16.4, 8.2 Hz, 2H), 8.11 (t, *J* = 7.7 Hz, 3H), 8.06 (d, *J* = 8.4 Hz, 1H), 7.76–7.66 (m, 1H), 7.56 (dt, *J* = 12.5, 7.5 Hz, 2H), 7.49 (t, *J* = 7.6 Hz, 1H). ^13^C NMR (126 MHz, DMSO) δ 166.4, 154.1, 146.4, 138.9, 135.2, 133.2, 132.0, 129.5, 129.4, 127.3, 126.3, 125.5, 123.6, 123.0, 120.4, 111.7.

### *N*-[4-(1,3-Benzothiazol-2-yl)phenyl]-4,6-dichloro-1,3,5-triazin-2-amine (**5**)

To a stirred solution of amine (1 equiv) in DCM (10 mL) were added cyanuric chloride (1 equiv) and triethylamine (1 equiv). The reaction was stirred for 8–12 h at room temperature. The precipitate obtained was filtered and washed thrice with diethyl ether to obtain the desired product (compound **5**, 76 mg) with a yield of 26%. ^1^H NMR (500 MHz, DMSO) δ 11.42 (s, 1H), 8.16–8.06 (m, 3H), 8.02 (m, *J* = 8.1, 1.2, 0.6 Hz, 1H), 7.80 (d, *J* = 8.8 Hz, 2H), 7.52 (m, *J* = 8.3, 7.2, 1.3 Hz, 1H), 7.43 (m, *J* = 8.3, 7.2, 1.2 Hz, 1H). ^13^C NMR (126 MHz, DMSO) δ 170.2, 167.1, 164.2, 154.1, 140.3, 134.9, 129.4, 128.5, 127.1, 125.9, 123.2, 122.8, 121.9.

### *N*-(4,6-Dichloro-1,3,5-triazin-2-yl)-5-nitro-1,2-benzothiazol-3-amine (**6**)

Cyanuric chloride (138 mg, 0.75 mmol, 1.0 equiv) was dissolved in THF (6.0 mL). The reaction mixture was cooled to 0 °C on an ice bath and treated with DIPEA (117 μL, 0.67 mmol, 0.9 equiv) at 0 °C. After 5 min, the reaction mixture was treated with 3-amino-5-nitrobenzisothiazole (146 mg, 0.75 mmol, 1.0 equiv) and stirred at 0 °C for 30 min. Then, the ice bath was removed and stirred at room temperature. The reaction progression was monitored by TLC (hexane/ethyl acetate; 7:3). After 30 min, the crude was purified by FCC (hexane/ethyl acetate; 8:2) and a clean product compound **6** (66 mg) was obtained with a yield of 26%. Yellow color solid. ^1^H NMR (500 MHz, DMSO) δ 11.91 (s, 1H), 8.80 (d, *J* = 2.7 Hz, 1H), 8.57 (dd, *J* = 9.1, 2.7 Hz, 1H), 7.92 (d, *J* = 9.0 Hz, 1H). ^13^C NMR (126 MHz, DMSO) δ 170.3, 165.5, 145.4, 144.7, 129.8, 129.4, 127.8, 115.4, 109.2. IR (solid) v/cm^–1^: 3303, 3118, 3084, 2233, 1571, 1535, 1504.

### *N*-(4-Chloro-1,3,5-triazin-2-yl)-5-nitro-1,2-benzothiazol-3-amine (**7**)

2,4-Dichloro-1,3,5-triazine (195 mg, 1.00 mmol, 1.0 equiv) was dissolved in THF (10.0 mL). The reaction mixture was cooled to 0 °C in an ice bath. 3-Amino-5-nitrobenzisothiazole (150 mg, 1.00 mmol, 1.0 equiv) was added and stirred at 0 °C for 30 min. Then, the ice bath was removed and stirred at room temperature for 48 h. The reaction progression was monitored by TLC (hexane/ethyl acetate; 7:3), the crude was purified by FCC (hexane/ethyl acetate; 8:2), and a clean product compound **7** (66 mg) was obtained with a yield of 26%. Yellow color solid. ^1^H NMR (500 MHz, CDCl_3_) δ 8.88 (d, *J* = 9.4 Hz, 1H), 8.78 (s, 1H), 8.62–8.44 (m, 2H), 8.14 (s, 1H). ^13^C NMR (126 MHz, CDCl_3_) δ 171.5, 168.0, 163.9, 144.5, 142.9, 129.3, 128.5, 121.2, 114.1, 103.0. IR (solid) v/cm^–1^: 3221, 3063, 2242, 1616, 1567, 1545, 1494, 1397.

### *N*-(5-Nitro-1,2-benzothiazol-3-yl)-2-phenylacetamide (**8**)

3-Amino-5-nitro-benzothiazole (150 mg, 0.77 mmol) was dissolved in 3 mL of pyridine. Then, phenylacetyl chloride was slowly added (0.11 mL, 0.85 mmol) and the reaction mixture was stirred for 4 h at room temperature. The reaction mixture was poured into ice, neutralized by 2 N HCl, then extracted with dichloromethane (15 mL × 3), and dried over anhydrous magnesium sulfate. The crude product was purified by column chromatography (hexane/ethyl acetate, 4:1 v/v) to obtain compound **8** as a pale yellow powder (192 mg, 81%). ^1^H NMR (500 MHz, DMSO) δ 10.78 (s, 1H), 8.70 (d, *J* = 2.7 Hz, 1H), 8.46 (dd, *J* = 9.2, 2.7 Hz, 1H), 7.98 (d, *J* = 9.2 Hz, 1H), 7.37–7.31 (m, 4H), 7.28–7.23 (m, 1H), 3.81 (s, 2H). ^13^C NMR (126 MHz, DMSO) δ 170.7, 146.4, 143.6, 135.5, 129.8, 129.8, 129.4, 128.9, 127.3, 125.0, 115.6, 106.2, 43.1. IR (solid) v/cm^–1^: 3187, 3010, 2228, 1682, 1580, 1505, 1405.

### *N*-(5-Nitro-1,2-benzothiazol-3-yl)-2-phenylacetamide (**9**)

3-Amino-5-nitrobenzisothiazole (98 mg, 0.50 mmol, 1.0 equiv) was dissolved in THF (15.0 mL). The reaction mixture was cooled to 0 °C in an ice bath. The reaction mixture was charged with 2-thiopheneacetyl chloride (64 μL, 0.52 mmol, 1.05 equiv) and stirred for 30 min at 0 °C. After 30 min, it was gradually warmed to room temperature and stirred until all of the amine starting materials were consumed, which was followed by TLC (hexane/ethyl acetate; 7:3). Then, the reaction mixture was directly loaded to a column and purified by FCC (hexane/ethyl acetate; 7:3). The clean product (compound **9**, 20 mg) was obtained with a yield of 13%. Yellow color solid. ^1^H NMR (500 MHz, DMSO) δ 9.38 (s, 1H), 8.10 (dd, *J* = 9.6, 2.4 Hz, 1H), 7.69 (d, *J* = 9.6 Hz, 1H), 7.45 (d, *J* = 5.1 Hz, 1H), 7.04 (dd, *J* = 30.3, 4.0 Hz, 2H), 4.28 (s, 2H). ^13^C NMR (126 MHz, DMSO) δ 169.8, 163.8, 158.1, 142.0, 135.6, 127.7, 127.4, 126.2, 122.6, 122.4, 120.3, 119.8, 35.9. IR (solid) v/cm^–1^: 3280, 3090, 1686, 1603, 1527, 1495, 1418, 1316. HRMS-ESI (*m*/*z*): [M + H]^+^ calcd for C_13_H_9_N_3_O_3_S_2_, 320.01578, found [M + H]^+^ 320.0015.

### *N*-(5-Nitro-1,2-benzothiazol-3-yl)acetamide (**10**)

3-Amino-5-nitrobenzisothiazole (156 mg, 0.80 mmol, 1.0 equiv) was dissolved in DMF (2 mL) at room temperature. Then, the reaction mixture was cooled to 0 °C in an ice bath. At 0 °C, acetyl chloride (57 μL, 0.80 mmol, 1.0 equiv) was added. The reaction mixture was stirred for 30 min at 0 °C and then gradually warmed to room temperature. The mixture was stirred at room temperature until all amine starting materials were consumed, which was monitored by TLC (hexane/ethyl acetate; 7:3). The crude mixture was evaporated by vacuum and purified by FCC (hexane/ethyl acetate; 9:1 to 7:3) to obtained a pure product, compound **10** (12 mg), with a yield of 5%. R_f_: 0.3 (hexanes: EtOAc; 7:3). Yellow color solid. ^1^H NMR (500 MHz, CDCl_3_) δ 9.25 (dd, *J* = 2.6, 0.5 Hz, 1H), 8.90 (dd, *J* = 9.4, 0.6 Hz, 1H), 8.58 (dd, *J* = 9.4, 2.5 Hz, 1H), 2.71 (s, 3H), 2.66 (s, 3H). ^13^C NMR (126 MHz, DMSO) δ 170.0, 164.0, 158.0, 141.9, 122.5, 122.3, 120.4, 119.5, 22.6. IR (solid) v/cm^–1^: 3284, 1690, 1603, 1518, 1492, 1318, 1239. HRMS-ESI (*m*/*z*): [M + H]^+^ calcd for C_9_H_7_N_3_O_3_S, 238.02808, found [M + H]^+^ 238.0137.

### 2-Chloro-*N*-(5-nitro-1,2-benzothiazol-3-yl)acetamide (**11**)

3-Amino-5-nitro-benzothiazole (200 mg, 1.02 mmol) was dissolved in 2 mL of DMF at 0 °C. Anhydrous potassium carbonate (213 mg, 1.54 mmol) was then added, and the reaction mixture was stirred for 30 min. Chloroacetyl chloride (0.16 mL, 2.04 mmol) was then added in small portions, and the reaction was stirred overnight at room temperature. The crude product was purified by column chromatography (hexane/ethyl acetate, 5:1 v/v) to obtain compound **11** as a yellow powder (211 mg, 76%). ^1^H NMR (500 MHz, DMSO) δ 9.28 (d, *J* = 2.4 Hz, 1H), 8.07 (dd, *J* = 9.6, 2.4 Hz, 1H), 7.93 (s, 1H), 7.68 (d, *J* = 9.6 Hz, 1H), 4.64 (s, 2H). ^13^C NMR (126 MHz, DMSO) δ 166.4, 163.2, 162.8, 158.1, 142.2, 122.6, 122.4, 120.1, 42.4. IR (solid) v/cm^–1^: 3105, 2838, 1710, 1657, 1604, 1572, 1536. HRMS-ESI (*m*/*z*): [M + H]^+^ calcd for C_9_H_6_ClN_3_O_3_S, 271.98908, found [M + H]^+^ 271.1367.

### *N*-Methanesulfonyl-*N*-(5-nitro-1,2-benzothiazol-3-yl)methanesulfonamide (**12**)

3-Amino-5-nitro-benzothiazole (200 mg, 1.02 mmol) was dissolved in 3 mL of pyridine. Methanesulfonyl chloride (0.15 mL, 2.04 mmol) was added gradually, and the reaction mixture was stirred overnight at room temperature. The reaction mixture was poured into ice, neutralized by 2 N HCl, then extracted with dichloromethane (15 mL × 3), and dried over anhydrous magnesium sulfate. The crude product was purified by column chromatography (hexane/ethyl acetate, 5:1 v/v) to obtain the compound as a yellow powder (265 mg, 79%). ^1^H NMR (500 MHz, DMSO) δ 8.96 (s, 1H), 8.61 (d, *J* = 8.2 Hz, 1H), 8.17 (d, *J* = 8.3 Hz, 1H), 3.66 (s, 6H). ^13^C NMR (126 MHz, DMSO) δ 148.84, 140.9, 134.7, 129.9, 129.8, 116.9, 115.4, 44.2. IR (solid) v/cm^–1^: 3046, 2242, 1612, 1528, 1351, 1162.

### *N*-Ethyl-1-[(ethylcarbamoyl)(5-nitro-1,2-benzothiazol-3-yl)amino]formamide (**13**)

3-Amino-5-nitrobenzisothiazole (131 mg, 0.80 mmol, 1.0 equiv) was dissolved in THF (10.0 mL). The reaction mixture was charged with ethyl isocyanate (126 μL, 1.6 mmol, 2.00 equiv) and stirred and followed by TCL (hexane/ethyl acetate 7:3) until all of the amine starting materials were consumed. Then, the reaction mixture was diluted with hexane (15 mL) and the resulting precipitate was filtered and washed with (hexane/Et_2_O 1:1). The product compound **13** (159 mg) was obtained with a yield of 59%. Yellow color solid. ^1^H NMR (500 MHz, DMSO) δ 8.74 (d, *J* = 2.5 Hz, 1H), 8.66 (t, *J* = 5.8 Hz, 1H), 8.49–8.40 (m, 1H), 8.37 (d, *J* = 9.4 Hz, 1H), 8.02 (d, *J* = 5.6 Hz, 1H), 3.23 (d, *J* = 6.1 Hz, 4H), 1.12 (q, *J* = 6.7 Hz, 6H). ^13^C NMR (126 MHz, DMSO) δ 170.6, 165.0, 152.2, 149.0, 141.7, 128.6, 122.8, 120.0, 118.0, 36.0, 35.9, 15.2, 15.0. IR (solid, v/cm^–1^): 3366, 3291, 3978, 1660, 1618, 1607, 1509, 1469, 1325,1307,1264,1242.

### Chemical and Peptide/Protein Source

Hexafluoroisopropanol (HFIP), dimethylsulfoxide (DMSO), and thioflavin T (ThT) were purchased from Alfa Aesar (Ward Hill, MA). 4-(2-Benzothiazolyl)aniline (BTA) and 5-nitro-1,2-benzothiazol-3-amine (5-NBA) were obtained from Sigma-Aldrich (Burlington, MA). Synthetic human IAPP and TTR fragments 81–127 were purchased from AnaSpec (Fremont, CA). TTR fragments 1–25, 26–50, 51–75, 76–100, 81–105, 101–125, and 101–125 were synthesized and obtained from GenScript (Piscataway, NJ). Recombinant α-syn was purchased from rPeptide (Watkinsville, GA).

Concerning the preparation of tau 2N4R, the bacterial expression plasmid consisting of the vector pRK172 carrying a cDNA encoding the human Tau 2N4R isoform was a kind gift of Dr. David Eliezer (Weill Cornell Medicine). For protein expression, *E. coli* BL21(DE3) cells were transformed with the plasmid and grown in LB media supplemented with ampicillin (100 μg/mL). Protein overexpression was induced by the addition of 1 mM of IPTG for 4 h at 37 °C, and cells were pelleted by centrifugation at 6000*g* for 15 min at 4 °C. The cells were resuspended in lysis buffer (20 mM MES, 400 mM NaCl, 0.2 mM MgCl_2_, 1 mM EGTA, protease inhibitor cocktail (P8340, Sigma-Aldrich), 0.25 mg/mL lysozyme, and 1 μg/mL DNase I, pH 6.8) and lysed by a French press cell disruptor at 4 °C, and the lysate was boiled for 20 min. Denatured proteins were pelleted by centrifugation at 30,000*g* for 30 min at 4 °C, and the supernatant was dialyzed overnight against cation-exchange buffer (20 mM MES, 50 mM NaCl, 1 mM MgCl_2_, 1 mM EGTA, 2 mM DTT, 0.1 mM PMSF, pH 6.8). The dialysate was loaded onto a HiPrep SP HP column, and proteins were eluted with a linear gradient ranging from 50 mM to 1 M NaCl. Fractions containing tau isoform 2N4R were pooled, and the resulting protein solution was dialyzed against PBS (pH 7.4) and stored at −80 °C.

Procedures to avoid oligomerization prior to the experiment have been performed as follows. Fresh batches of purified tau from the FPLC (Akta) and freshly received batches of commercial protein/peptides have been used for each experiment. Proteins and peptides purchased commercially from rPeptide (α-syn) and AnaSpec (hIAPP, TTR81-127) have been validated by the company with proper quality control to ensure the monomeric state of the protein. Concerning IAPP, fibril formation occurs quickly (about 1 h). For this reason, the peptide was solubilized in HFIP at a concentration of 1 mM and kept at 4 °C overnight prior to ThT assays.

### Thioflavin T (ThT) Fluorescence Assays

Thioflavin T (ThT) fluorescence assays were used to monitor fibril formation of recombinant α-syn, recombinant tau isoform 2N4R, synthetic IAPP, and synthetic TTR peptides treated with BTA and its derivatives. IAPP ThT assay was performed in 10 mM PBS (pH 7.4) at a final concentration of 10 μM for both IAPP and ThT as published previously.^38^ Kinetics of α-syn fibrillization were performed using a concentration of 2 μM using a similar procedure as published previously.^39,40^ TTR fragment kinetics of fibril formation was assessed at 10 μM in 100 mM sodium acetate buffer supplemented with 100 mM KCl and 1 mM ethylenediaminetetraacetic acid (EDTA, pH 4) with 20 μM ThT. The fluorescence emission experiments were performed with the excitation and emission wavelengths set at 440 and 485 nm, respectively, with a Synergy HT multimode microplate reader (BioTek, Winooski, VT). Samples were measured in three replicates, and the experiments were repeated three times using at least two different stock solutions. For each time point, arbitrary units of fluorescence were calculated from the mean values normalized against the maximum value in each completed assay. The lag time for each condition was calculated as previously described.^39^ All results contained in histograms were presented as mean ± SEM. Data were analyzed by the one-way analysis of variance with Dunnett’s multiple comparisons between controls and compounds. Differences were considered statistically significant at p < 0.05.

Compounds with the highest antifibrillary activity were tested at 3.125, 6.25, 12.5, 25, 50, and 100 μM to obtain dose–response curves with prepared α-syn and 2N4R tau at 2 and 6 μM, respectively. Compounds were tested with α-syn using the procedure published previously.^40^ Concerning the tau (isoform 2N4R) kinetics of fibril formation, measurements of ThT fluorescence were performed with a solution of the protein diluted to a final concentration of 6 μM in PBS (pH 7.4) supplemented with 1.5 μM heparin, 20 μM ThT, 2.5 mM dithiothreitol (DTT), and 100 μM compound. Aliquots of the diluted protein solution (100 μL each) were pipetted into the wells of a 96-well plate, and a Teflon ball was added to each well. The plate was incubated at 37 °C with constant shaking at 1000 rpm in a Tecan Spark plate reader. ThT fluorescence was measured every 15 min with excitation and emission wavelengths respectively, of 440 and 480 nm, and the data were plotted using GraphPad Prism.

### Transmission Electron Microscopy (TEM)

After performing analysis with ThT fluorescence assay, TEM was utilized to detect fibril formation. A volume of 10 μL was applied on a 400-mesh Formvar-carbon-coated copper grid (Electron Microscopy Sciences, Hatfield, PA). The grids were incubated for 1 min and washed three times with distilled water. They were carefully air-dried and incubated for 1 min in a fresh solution of 1% uranyl acetate. Samples were air-dried and observed using a transmission microscope. Visualization of the grids was performed with transmission electron microscopy (JEOL 1400 Flash, Japan). Acquisition of pictures was performed with the following settings: accelerating voltage of 100 kV and magnification of 20k and/or 40k.

### Photoinduced Cross-Linking of Unmodified Protein (PICUP) Assay

To induce oligomerization by cross-linking, α-syn (from Rpeptide, LLC) and tau isoform 2N4R were diluted in 10 mM phosphate buffer (pH 7.4) to reach a final concentration of 60 and 6 μM, respectively. Different compounds were added to the protein solution at a final concentration of 50 μM. To confirm the gradual effect of our compounds on the inhibition of α-syn oligomerization, compounds were tested at a final concentration of 3.125, 6.25, 12.5, 25, 50, 100, and 200 μM. The controls consisted of samples without light exposition, without Ru(bpy)_3_ or ammonium persulfate, and without compound (i.e., 0.125% DMSO). The cross-linking reaction was initiated by the addition of 2 μL of Ru(bpy)_3_ (300 μM final concentration) and 2 μL of ammonium persulfate (6 mM final concentration).^40,41^ Samples were subjected to light immediately. Light exposure was of a 1 second duration for α-syn and a 3 second duration for tau isoform 2N4R, with a 53 W (120 V) incandescent lamp installed in a homemade dark box. Each tube contained a final volume of 20 μL. After irradiation, 8.3 μL of Laemmli loading buffer containing 15% β-mercaptoethanol was immediately added to the solution, followed by incubation at 95 °C for 10 min. The cross-linked samples were separated on a 16% SDS-PAGE gel and visualized by Coomassie blue staining.

### α-Syn (or αS) Inclusion-Forming Neuroblastoma Cell Experiment

Doxycycline(dox)-inducible neuroblastoma cells M17D-TR/αS-3K::YFP have been used previously.^42^ 96-well plates were used with a cellular density of 30,000 cells per well. Compounds were added after 24 h, and αS-3K::YFP transgene expression was induced 48 h later. Induction was done by adding 1 μg per mL (final concentration) dox to culture media. Cells were incubated in the Incucyte Zoom 2000 platform (Essen Biosciences), and images (green, bright field) were taken continuously. Endpoint analysis of inclusion formation or growth was performed 48 h after induction (96 h after plating). The Incucyte processing definition “Inclusions” was created as follows: parameters, fixed threshold, threshold (GCU) 50; edge split on, edge sensitivity 100; cleanup, hole fill (μm^2^): 10, adjust size (pixels): 0; filters, area (μm^2^): max 50, mean intensity: min 60, integrated intensity: min 2000. Cell confluence was measured by the processing definition “Cells”: parameters, segmentation adjustment 0.7; Cleanup, all parameters set to 0; filters, area (μm^2^): min 345.00. As described previously for the evaluation of protein expression by SDS-PAGE and Western blotting in the LiCor system, αS-specific monoclonal antibody 4B12 (Thermofisher, Waltham, MA; 1:1000) and a polyclonal antibody to GAPDH (Sigma-Aldrich, St. Louis, MO, G9545; 1:5000) were used.^43^

## Results and Discussion

### Chemistry

4-(Benzo[*d*]thiazol-2-yl)aniline (BTA) has been found as a potent inhibitor of fibril formation by testing small molecules in our laboratory. Small chemical modifications were then applied in the hope of keeping or improving the antiaggregation activity of BTA. The preparation of BTA derivatives with urea (**1**), thiourea (**2**), sulfonamide (**3**), triazole (**4**), and triazine (**5**) linkers was executed. BTA derivatives containing urea (**1**) and thiourea (**2**) were obtained without difficulty by coupling BTA with the commercially available substituted respective isocyanate or isothiocyanate in anhydrous DCM at room temperature for 24 h. The BTA sulfonamide derivative (**3**) was synthesized by nucleophilic substitution reactions of BTA with 4-acetyl benzene sulfonyl chloride in anhydrous DCM in the presence of a base such as triethylamine. Concerning the BTA triazole derivative, compound **4** was prepared using BTA and 2-(trimethylsilyl) phenyl trifluoromethane-sulfonate.^37^ For the preparation of BTA triazine derivative, compound **5**, commercially available aromatic amines were reacted with cyanuric chloride in anhydrous DCM in the presence of a base such as triethylamine for 24 h. These compounds were not significantly capable of reducing aggregation, and we opted to apply chemical alterations on the commercial 5-NBA due to its antioligomer activity detected in the laboratory.

Compound **6** resulted from the reaction between the commercially available aromatic amine, 5-NBA, with cyanuric chloride in a similar manner to the procedure utilized for the preparation of compound **5**. Compound **7** was prepared with commercially available 2,4-dichloro-1,3,5-triazine in THF cooled to 0 °C on an ice bath before the introduction of 5-NBA. Low temperature was maintained for 30 min. The reaction was allowed at room temperature for 48 h. Compounds **8**–**11** were obtained from the slow addition of acetyl chloride derivatives to the 5-NBA. Some reactions were performed using a base (compound **8**), low temperature with a base (compound **11**), or low temperature without a base (compounds **9–10**). Compound **12** resulted from the 5-NBA solubilized in pyridine by the gradual addition of methanesulfonyl chloride. Compound **13** was prepared from the 5-NBA and chloroethylisocyanate. Compound **13** is the only derivative resulting in the diminution of oligomer formation. For this reason, we attempted to generate the BTA formamide derivative at room temperature or at 65 °C using tetrahydrofuran (THF). These reaction trials failed after 2 days as only the monosubstituted BTA resulted from the reaction condition attempted. All of the compounds were characterized using ^1^H NMR, ^13^C NMR, and HRMS or IR.

### BTA Is a General Inhibitor of Prone-to-Aggregate Proteins

**Figure 2:**
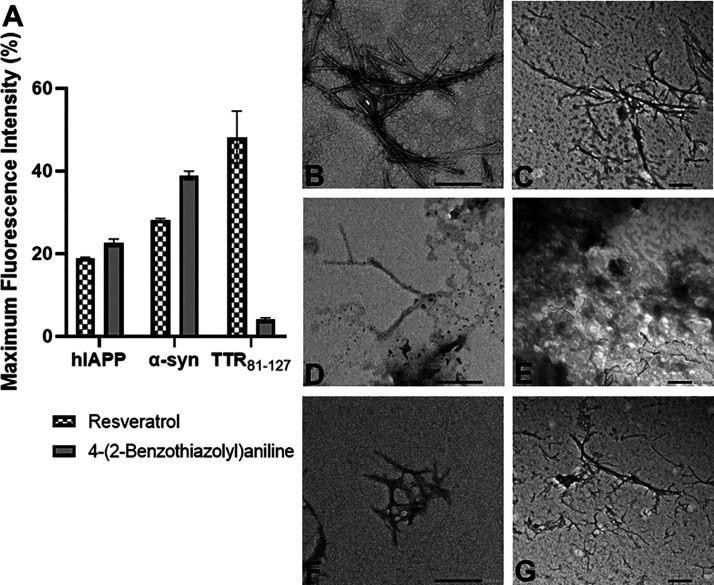
4-(Benzo[*d*]thiazol-2-yl)aniline (BTA) is a general inhibitor of fibril formation. (A) A bar graph depicting the arbitrary maximum fluorescence intensity in percentage for fibril type including IAAP, α-syn, and TTR_81–127_. Since IAPP, α-syn, and TTR_81–127_ had arbitrary percent fluorescence under 40% for both resveratrol BTA treatments, analysis via electron micrograph (EM) was performed. The error bars represent the individual standard error of mean (SEM) for each condition. (B–J) Electron micrograph (EM) of peptides incubated with either 100 μM resveratrol, 100 μM BTA, or 0.1% DMSO control at 37 °C for 1 h (10 μM of IAPP) in PBS, 24 h (2 μM of α-syn in PBS), and 24 h (10 μM of TTR_81–127_) in sodium acetate buffer, pH 4. (B) IAPP with ≤0.1% DMSO at 40k magnification. (C) TTR_81–127_ with ≤0.1% DMSO at 40k magnification. (D) IAPP with 100 μM BTA at 40k magnification. (E) TTR_81–127_ with 100 μM BTA at 40k magnification. (F) IAPP with 100 μM resveratrol at 40k magnification. (G) TTR_81–127_ with 100 μM resveratrol at 40k magnification. Scale bars = 200 nm.

To determine if BTA is a general or specific inhibitor of fibril formation, we studied the kinetics aggregation of α-syn, human IAPP, and TTR_81–127_ in the presence and absence of BTA and resveratrol (general inhibitor of fibril formation). IAPP, α-syn, and TTR_81–127_ had arbitrary percent fluorescence under 40% for both resveratrol and BTA treatments. Transmission electron micrograph (TEM) was performed as a follow-up to confirm fibril alteration (Figure 2). IAPP and TTR_81–127_ were treated with either 100 μM resveratrol, 100 μM BTA, or 0.1% DMSO control at 37 °C for 1 day in PBS (IAPP) or sodium acetate buffer (TTR_81–127_) before analysis *via* TEM microscopy. Each sample was observed at 40k magnification (IAPP and TTR_81–127_). Magnification depended on the ability to observe the fibrils, and the best magnification was selected for each type, with scale bars kept at 200 nm. Imaging via TEM confirmed reduced fibril formation in TTR fibrils treated with BTA and resveratrol (Figure 2G,J). The control-treated samples featured the classic linear, branched fibril structures (Figure 2B–C), while less fibrils were observed on the copper grid containing samples treated with 100 μM BTA or resveratrol (Figure 2D–G).

**Figure 3:**
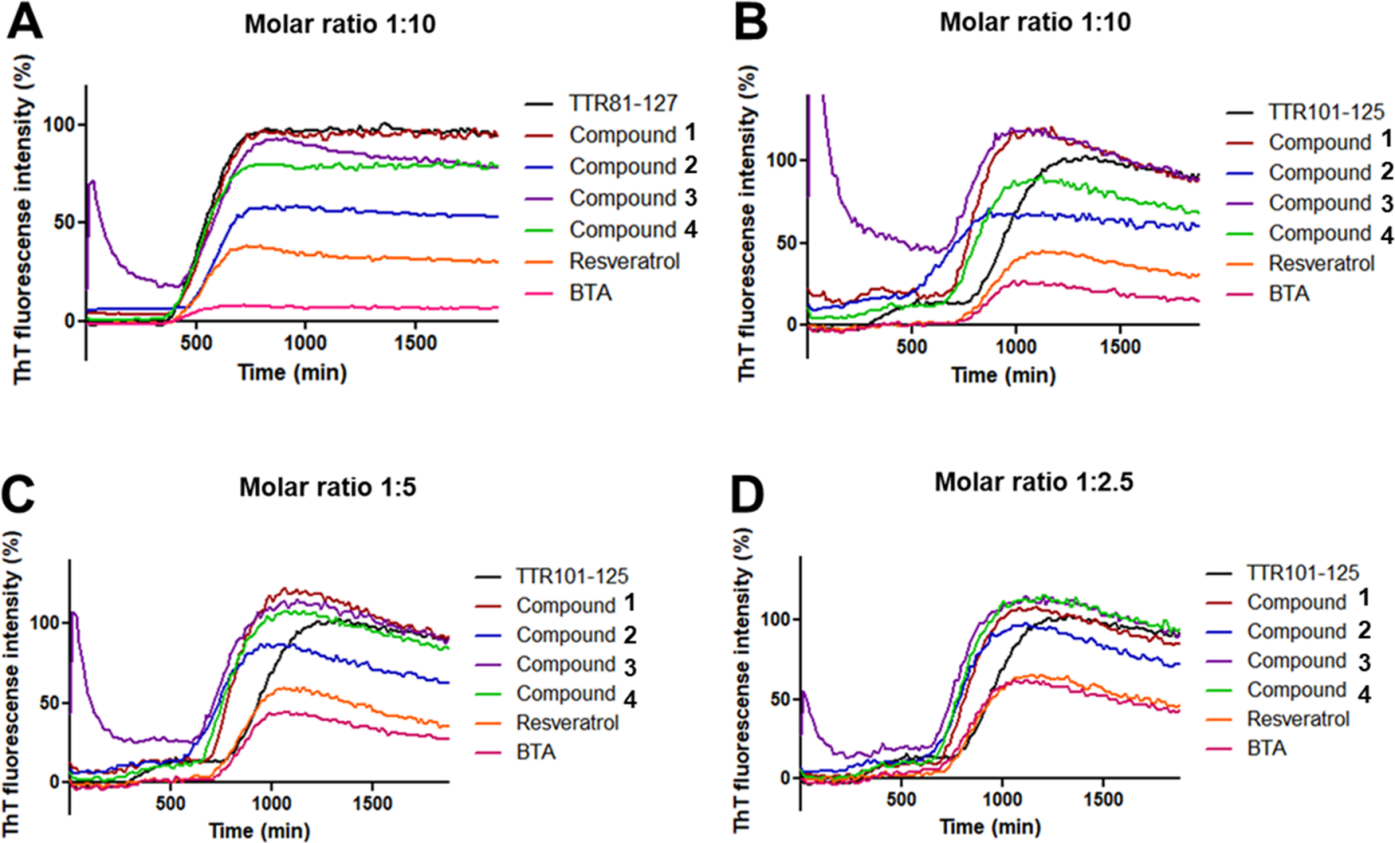
Compounds **1–4** failed to substantially abrogate the fibril formation of both TTR fragments (TTR_81–127_, TTR_101–125_). (A) TTR_81–127_ fibril formation of 4-(benzo[*d*]thiazol-2-yl)aniline (BTA) derivatives **1–4**, resveratrol (positive control), and BTA at 100 μM (molar ratio 1:10) assessed by ThT fluorometric assays in a time-dependent manner in the presence of DMSO (0.1%, control; CTRL). (B) TTR_101–125_ fibril formation of BTA derivatives **1–4**, resveratrol (positive control), and BTA at 100 μM (molar ratio 1:10) by ThT fluorometric assays in a time-dependent manner. Panels (C) and (D) are the same as panel (B) but evaluate compounds at a final concentration of 50 μM (molar ratio 1:5) and 25 μM (molar ratio 1:2.5).

Truncated peptides, TTR_81–127_ and TTR_101–125_, were treated with BTA and four derivatives: compounds **1** (urea), **2** (thiourea), **3** (sulfonamide), and **4** (triazole). ThT experiments were performed using the fragment peptides TTR_81–127_ (Figure 3A) and TTR_101–125_ (Figure 3B) to validate the antiaggregation effect on these peptides. BTA and, to a lesser extent, compound **2** reduced fibril formation for both fragments at a molar ratio of 1:10 (Figure 3A,B). BTA and compounds **1–4** were tested at a lower concentration using the TTR_101–125_ fragment. Compound **2** was weak in inhibiting TTR_101–125_ fibril formation at 50 μM (molar ratio 1:5) (Figure 3C). BTA continued to reduce TTR_101–125_ fibrils at 25 μM (molar ratio 1:2.5) (Figure 3D). The antifibrillary effects of BTA and compound **2** were confirmed by TEM (please see the Supporting Information). The antiaggregation effect of compound **2** was weaker in comparison to BTA, and more compounds were designed (Table 1). Our lab explored the 5-NBA derivatives recently and observed a significant improvement in the antiaggregation activity.

### Antifibrillary Effect of Additional BTA and 5-NBA Derivatives

To provide more insights into the effect on BTA and 5-NBA derivatives, 13 compounds were synthesized and their antiaggregation activity on α-syn and TTR_81–127_ was compared with aniline (i.e., BTA or 5-NBA) (Table 1). We applied structural modifications on BTA and 5-NBA cores due to their antifibrillary activities. the identification as a potent inhibitor of fibrils. Both the antifibrillary and antioligomer effects were evaluated on α-syn. As a starting point, compound **5** is mostly related to BTA and previously prepared compounds **1–4**. Compounds **5–13** had a weak effect on the aggregation of TTR_81–127_, with 5-NBA exhibiting the lowest fluorescence intensity (54.7 ± 1.4%). Compounds **5–9** and **12** did not demonstrate a strong antifibrillary activity on α-syn. The 5-NBA (36.2 ± 3.1%), compound **10** (55.6 ± 3.2%), compound **11** (45.3 ± 12.9%), and compound **13** (16.4 ± 7.8%) were the best compounds to abrogate α-syn fibril formation. Interestingly, these compounds bear an *N*-acetamide (compound **10**), 2-chloro-*N*-acetamide (compound **11**), or an *N*-ethyl-1-formamide (compound **13**). The presence of larger substituents such as triazine (compounds **6** and **7**), phenyl ring (compound **8**), or thiophene (compound **9**) led to the significant loss of antifibrillary activity. All compounds **5–13** were tested for their antioligomer activity on α-syn at a concentration of 50 μM (Figure S5). Only the 5-NBA reduced the oligomer formation using a molar ratio of ∼1:1 (α-syn) and 1:5 (tau) (protein/compound) (Figure 5). Its derivative, compound **13**, reduced the oligomer formation at higher concentration (Figure 6).

### 5-NBA Exhibits Broad Antifibrillary Effect on Different Prone-to-Aggregate Proteins

**Figure 4:**
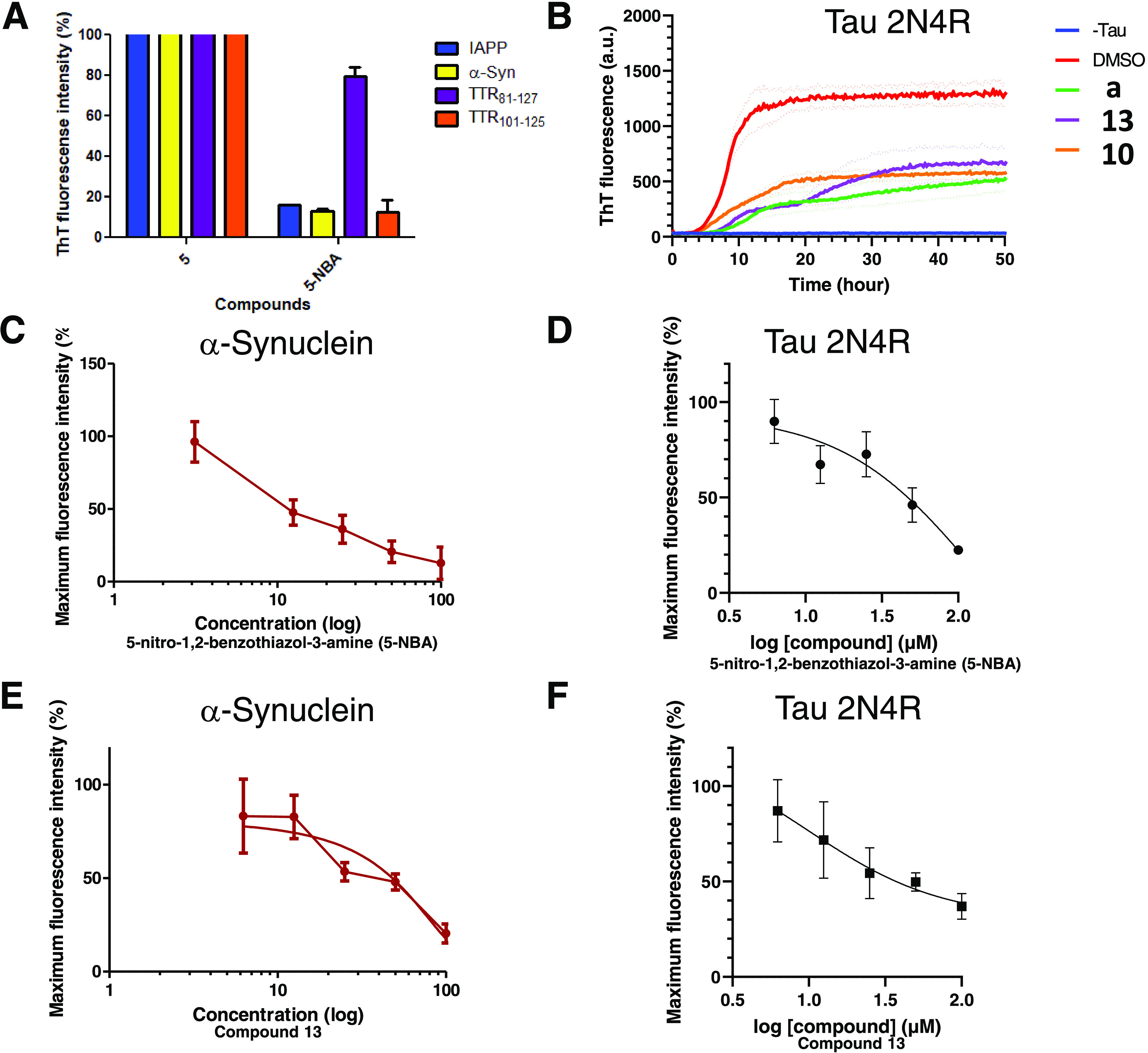
Antifibrillary effect of 5-nitro-1,2-benzothiazol-3-amine (5-NBA) and several derivatives on different prone-to-aggregate proteins with special emphasis on α-syn and tau isoform 2N4R. (A) Histogram representing thioflavin T fluorescence intensity of prone-to-aggregate proteins incubated with compound **5** (negative control, BTA derivatives) and 5-NBA. IAPP, TTR_81–127_, and TTR_101–125_ were tested at 10 μM. The final compound concentration corresponded to 100 μM. (B) Tau isoform 2N4R kinetics of fibril formation in the presence or absence of 5-NBA, compound **10** (*N*-(5-nitro-1,2-benzothiazol-3-yl)acetamide), and compound **13** (*N*-ethyl-1-[(ethylcarbamoyl)(5-nitro-1,2-benzothiazol-3-yl)amino]formamide). 5-NBA and compound **13** delayed the lag time. (C) Dose dependency of 5-NBA on the inhibition of α-syn fibril formation. (D) Similar dose dependency response but applied tau isoform 2N4R with 5-NBA. (E) Dose dependency of *N*-ethyl-1-[(ethylcarbamoyl)(5-nitro-1,2-benzothiazol-3-yl)amino]formamide (compound **13**) on the inhibition of α-syn fibril formation. Log(agonist) vs. normalized response (variable slope) was applied using Prism software and resulted in a LogIC50 of 48.00 ± 7.96 μM. (F) Dose dependency of *N*-ethyl-1-[(ethylcarbamoyl)(5-nitro-1,2-benzothiazol-3-yl)amino]formamide (compound **13**) on the inhibition of tau 2N4R fibril formation. For each concentration (3.125, 6.25, 12.5, 25, 50, 100 μM), triplicate data were collected at the plateau phase for α-syn and at 15 h for tau. For all ThT assays, α-syn was tested at 2 μM (A, C, E) and tau was utilized at 6 μM (B, D, F). The error bars represent the individual standard error of mean (SEM) for each condition evaluated in triplicate.

Three important prone-to-aggregate proteins, namely, IAPP, α-syn, and TTR (fragments TTR_101–125_ and TTR_81–127_), were examined for thioflavin T fluorescence intensity (%) using 5-NBA because of its outstanding antioligomer activity (Figure 4A). Compound **5** produced an FI of greater than 100% across all five proteins and was used as a negative control. 5-NBA was significantly more effective at preventing the aggregation of α-syn, TTR_101–125_, and IAPP. hIAPP antiaggregation activities of 5-NBA were comparable to those of BTA (Figure 2A). Based on the screen on IAPP, 5-NBA exhibited excellent antifibrillar activity resulting in a fluorescence intensity of 15.9 ± 0.2% and comparable to BTA (22.6 ± 0.9%). 5-NBA as a weaker antibribrillization effect on fragment TTR_81–127_ versus fragment TTR_101–125_.

We further examined the antiaggregation activity of tau isoform 2N4R using a small selection of compounds. Figure 4B displays the tau kinetic aggregation curves obtained from a ThT assay of 5-NBA, compound **10** (intermediate inhibitor of α-syn fibrillization), and compound **13** (best inhibitor of α-syn fibrillization). All reduced the production of tau isoform 2N4R fibrils, 5-NBA, demonstrating the best antifibrillary activity. However, only 5-NBA and compound **13** delayed the lag time. We examined the lead compounds impact on α-syn and tau aggregation kinetics at lower molar ratios and observed that there is a dose-dependent relationship between compound concentration and protein aggregation. Our data in panels C, D and E, F of Figure 4 demonstrate a link between a decrease in fluorescence intensity and the concentration of the 5-NBA and compound 13 (dose–response), respectively. When exposed to increased concentrations of the 5-NBA or its derivative (compound **13**), protein fibrillization was reduced in both α-syn and tau isoform 2N4R.

### 5-NBA Is an Early Stage Aggregation Inhibitor of α-Syn and Tau (2N4R)

**Figure 5:**
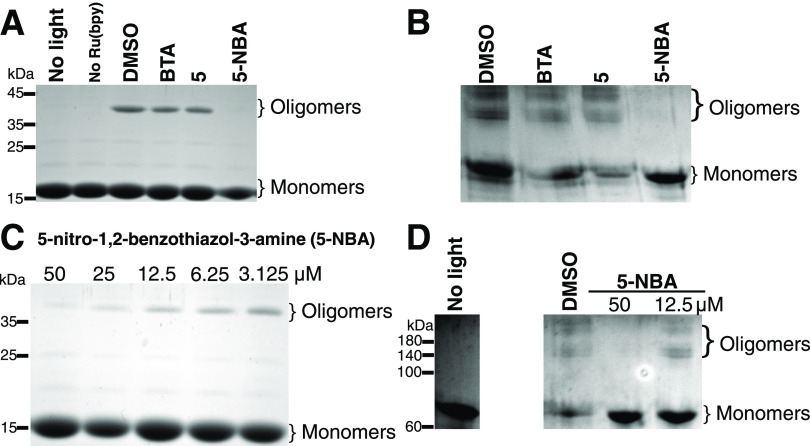
5-Nitro-1,2-benzothiazol-3-amine (5-NBA) reduced α-syn and tau isoform 2N4R oligomer formation by photoinduced cross-linking of unmodified proteins (PICUP). (A) α-Syn (60 μM) was cross-linked (PICUP assay) with 4-(benzo[*d*]thiazol-2-yl)aniline (BTA), 5-NBA, and compound **5** at 50 μM. DMSO, BTA, and compound 5 (BTA derivative) failed to prevent the formation of high molecular bands located between 35 and 45 kDa and corresponding to oligomers. Additional controls consist of no light and no cross-linking agent (no Ru(bpy)_3_), which resulted in no high molecular bands. (B) Tau isoform 2N4R (6 μM) was cross-linked (PICUP assay) with different compounds at 50 μM. (C) Dose-dependent inhibitory activity of 5-NBA on α-syn oligomerization. The protein (60 μM) was incubated with 5-NBA: 50 μM, 25 μM , 12.5 μM, 6.25 μM, and 3.125 μM. (D) Similar conditions were applied to evaluate the dose-dependent inhibitory activity on tau isoform 2N4R (6 μM). 5-NBA stopped the formation of α-syn and tau oligomers (un-cross-linked) in a dose-dependent manner. A lower concentration of 5-NBA showed a higher prominence of oligomer formation. Coomassie blue-stained polyacrylamide gels showed high-molecular-weight α-syn oligomers with control (0.125% DMSO).

**Figure 6:**
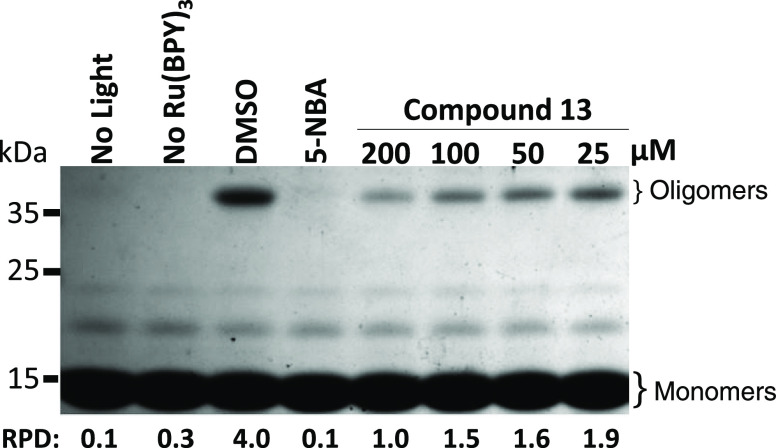
Dose-dependent inhibitory activity of compound **13** on α-syn oligomerization by PICUP. Compound **13** reduced the oligomer formation at a high concentration, i.e., 200 μM. 5-NBA was used as a positive control and tested at 200 μM. The protein was tested at 60 μM. High-molecular-weight α-syn oligomer results from control condition (0.125% DMSO). Additional controls consist of no light and no cross-linking agent (no Ru(bpy)_3_), which resulted in no cross-linking protein. The pixel density of the high-molecular-weight bands labeled as oligomers and the low molecular bands identified as monomers has been measured using image J. The pixel density of the higher molecular bands has been divided by the pixel density of the band corresponding to the monomeric state for each condition. The ratio is indicated as RPD (for relative pixel density) below the Coomassie blue-stained polyacrylamide gel.

To test the effectiveness of the small molecule at the early stage of aggregation, oligomer formation of α-syn (Figure 5A) and tau isoform 2N4R (Figure 5B) were induced in the presence of BTA, compound **5** (negative control), and 5-NBA at a concentration of 50 μM by performing a photoinduced cross-linking of unmodified protein (PICUP) assay. The resulting products after short-light exposure were analyzed on an SDS-PAGE gel stained with Coomassie blue. High-molecular-weight bands representing the oligomeric species become evident across the two proteins used. 5-NBA reduced α-syn and tau isoform 2N4R oligomer formation in comparison to the DMSO control (Figure 5A,B). Results from the PICUP assay indicated that BTA was not successful in preventing oligomerization of the two proteins, despite the inhibition of fibril growth. Compound **5**, which was used as a negative control, resulted in high-molecular-weight bands representing the oligomeric species of α-syn and tau 2N4R. 5-NBA reduced the oligomer formation in a dose-dependent manner (Figure 5C,D). We examined the antioligomer activity of compound **13** at a higher concentration since the delay of the lag time was observed in one of the tau ThT assays. The 5-NBA derivative, compound **13**, reduced substantially the α-syn oligomer formation at high concentration, i.e., 100 and 200 μM (Figure 6). In contrast to BTA, 5-NBA is an effective inhibitor of α-syn and tau oligomerization as shown by its potential to reduce both fibrillization and oligomerization via ThT and PICUP. Other derivatives presented in Table 1 were subjected to PICUP and failed to inhibit the α-syn oligomer formation. Results are available in supplemental data (Figure S5).

### Ultrastructural Changes of α-Syn and Tau Isoform 2N4R Treated with 5-NBA

**Figure 7:**
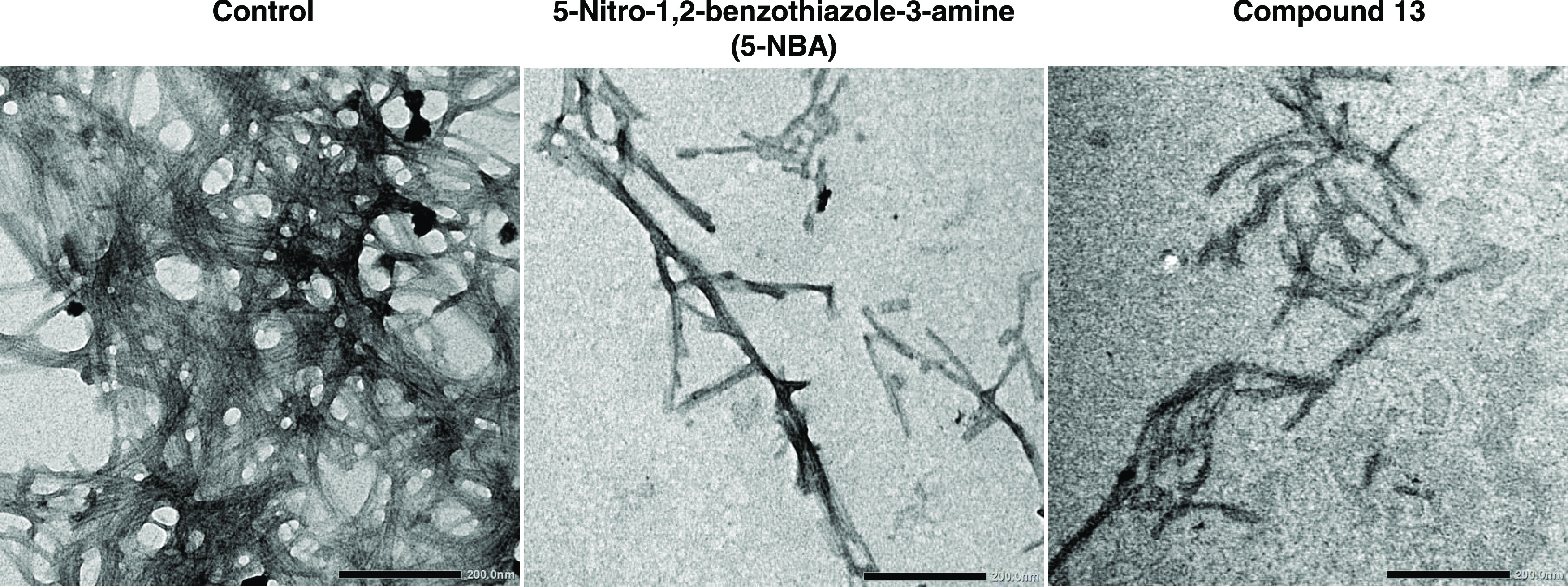
5-Nitro-1,2-benzothiazol-3-amine (5-NBA) and its derivative, compound **13**, reduced α-syn fibril formation as validated by transmission electron microscopy (TEM). α-Syn (2 μM) was incubated with DMSO (0.25%; “CTRL”), 5-NBA (100 μM), or compound **13** (at 100 μM) for ∼48 h prior to TEM visualization. High magnifications (40K) showed fewer fibrils in protein samples supplemented with 5-NBA and compound **13** in comparison with DMSO control. TEM results validate the reduction in fibrils monitored by ThT assays. Scale bars, 200 nm.

**Figure 8:**
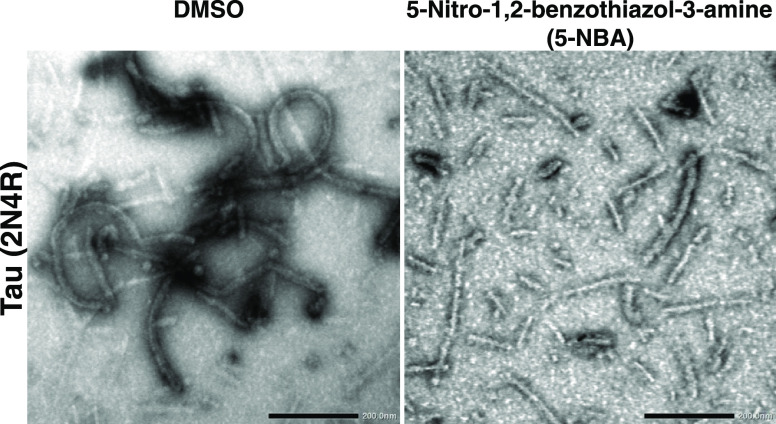
5-Nitro-1,2-benzothiazol-3-amine (5-NBA) reduced tau fibril formation as validated by transmission electron microscopy (TEM). Tau isoform 2N4R (6 μM) was incubated with DMSO (0.25%; “CTRL”) or 5-NBA (100 μM) for ∼50 h (i.e., in previously described experiments aimed at monitoring fibril formation by ThT fluorescence) prior to TEM visualization. Scale bars, 200 nm.

Transmission electron microscopy (TEM) analyses were performed using a solution of α-syn (2 μM) and tau isoform 2N4R (6 μM) at the end of kinetics of fibril formation (48 to 50 h incubation) with 0.25% DMSO or 5-NBA at 100 μM to visualize direct changes in fibril morphology (Figures 7 and 8). 5-NBA exhibited a clear effect in reducing α-syn and tau fibrillization. In contrast to control α-syn samples treated with DMSO without compound, short fibrils were seen in samples containing 5-NBA and compound **13** (Figure 7). The tau fibrils were shorter and less defined when compared to the DMSO control (Figure 8). The effect of compound **13** on tau fibrils was not confirmed by TEM due to the lower capability to reduce oligomer in comparison with 5-NBA. TEM data confirmed that 5-NBA was effective at reducing the formation of α-syn and tau isoform 2N4R fibrils.

### α-Syn (or αS) Inclusion Formation and Toxicity in Neuroblastoma Cells

**Figure 9:**
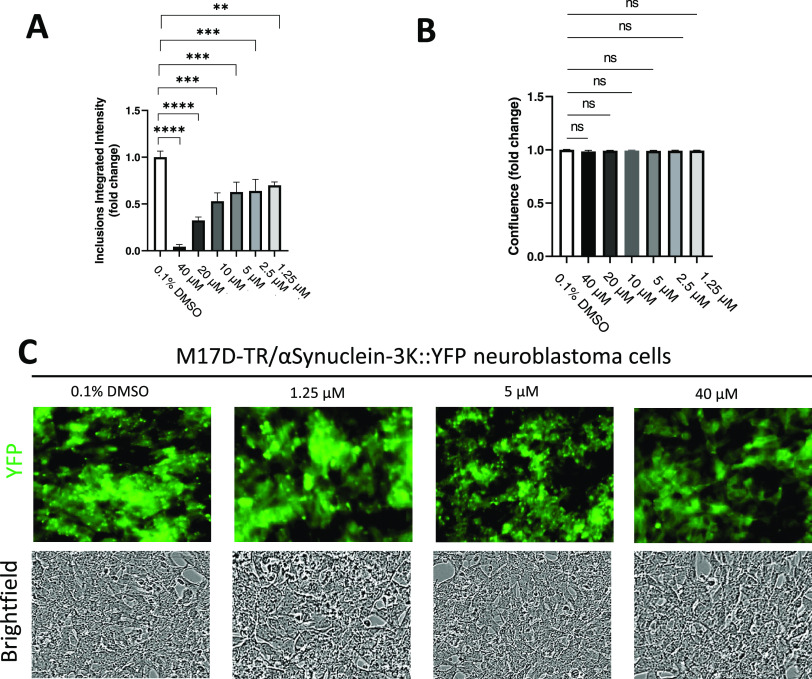
5-Nitro-1,2-benzothiazol-3-amine (5-NBA) abrogated the inclusion formation in M17D neuroblastoma cells that express inclusion-prone αS-3K::YFP. (A) Incucyte-based analysis of punctate YFP signals at *t* = 96 h, normalized to 0.1% DMSO. 8 independent experiments (*N* = 8) were performed. Student’s *t*-test, ****, *p* < 0.0001, ***, *p* < 0.001, **, *p* < 0.01. (B) Same as panel A, but confluence was plotted. (C) M17D cells that express an αS-3K::YFP fusion protein (doxycycline (dox)-inducible) were treated with 0.1% DMSO (vehicle control) or different concentrations of 5-NBA at *t* = 24 h. Cells were induced with dox at *t* = 48 h. Representative images (YFP, top; bright field, bottom).

**Figure 10:**
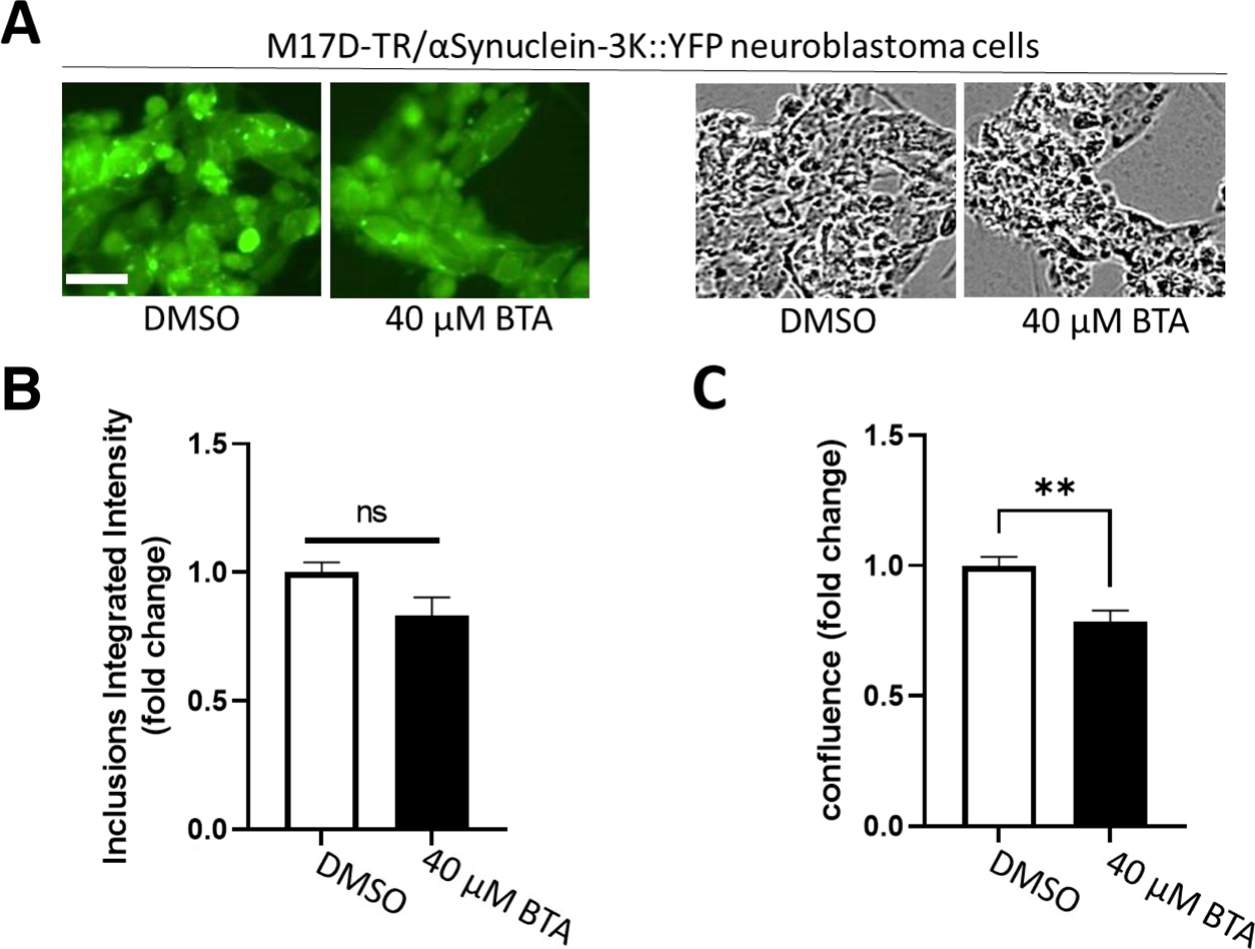
4-(Benzo[*d*]thiazol-2-yl)aniline (BTA) reduced the confluence but not the inclusion formation in M17D neuroblastoma cells that express inclusion-prone αS-3K::YFP. (A) M17D cells that express an αS-3K::YFP fusion protein (dox-inducible) were treated with 0.1% DMSO (vehicle control) or 40 μM BTA at *t* = 24 h. Cells were induced with doxycycline (dox) at *t* = 48 h. Representative images (YFP, left; bright field, right); scale bar, 25 μm. (B) Incucyte-based analysis of punctate YFP signals at *t* = 96 h, normalized to 0.1% DMSO. 8 independent experiments (*N* = 8) were performed. Student’s *t*-test, **, *p* < 0.01. (C) Same as panel B, but confluence was plotted.

αS E35K + E46K + E61K (= αS-3K) “amplifies” the familial-PD-linked αS missense mutation E46K. This model is known to generate round-shaped cytoplasmic inclusions in cultured cells.^44,45^ αS-3K expression leads to cell stress/toxicity, which results in a delayed growth of neuroblastoma cells.^42^ Using the same system in previous studies, stearoyl-CoA desaturase inhibitors prevented both αS inclusion formation^43^ and αS-induced cytotoxicity.^42^ Herein, we employed neuroblastoma cells, M17D, that express an αS-3K::YFP fusion protein in a doxycycline-inducible fashion. The α-syn model was used to evaluate the effect of BTA and 5-NBA on the inclusion formation. The effect of compound **13** on the inclusion was not tested due to the lower antioligomer effect in comparison with 5-NBA. 24 h of induction of αS-3K: YFP resulted in pronounced round YFP-positive inclusions in the presence of vehicle (DMSO) alone, whereas 5-NBA reduced the number of inclusions in a dose-dependent manner (starting at 10 μM) without any effect on the cell confluence (Figure 9). In contrast, BTA (40 μM) did not affect the number of inclusions present in the neuroblastoma cells (Figure 10). Also, the treatment of BTA led to a reduction in confluence in contrast to 5-NBA.

## Conclusions

In this work, we evaluated the inhibitory potential of BTA and its derivatives on the aggregation of IAPP, TTR_81–127_, TTR_101–125_, α-syn, and tau 2N4R (only with the best compounds). We first synthesized a series of 13 compounds derived from BTA or 5-NBA. We performed a ThT fluorescence assay with BTA on a few prone-to-aggregate proteins (IAPP, α-syn, TTR_81–127_) and validated the formation of fibrils with TEM to demonstrate the effect of BTA in reducing fibril formation. In addition, we performed a ThT assay using transthyretin (TTR_81–127_, TTR_101–125_) on BTA and our original four compounds at various molar ratios and found that the four compounds were not more effective than BTA in reducing fibrillization. We then evaluated fibrillization of our remaining compounds using ThT and found that only 5-NBA was effective in reducing α-syn oligomer and fibril formation. Its antifibrillar activity is similar to BTA and affects most of our prone-to-aggregate proteins. In contrast to 5-NBA, BTA does not inhibit the oligomer formation. We also performed a ThT assay and PICUP on tau 2N4R using 5-NBA and witnessed a reduction in fibrillization and oligomerization. We followed up with a ThT and PICUP dose–response analyses and were able to detect a concentration-dependent effect of 5-NBA on α-syn and tau 2N4R fibrillization and oligomerization. TEM analysis allowed us to confirm the presence of α-syn and tau 2N4R fibrils when treated with DMSO control and the reduction of these fibrils when treated with 5-NBA. One derivative of 5-NBA, compound **13**, was capable of inhibiting α-syn and tau fibril formation in a dose-dependent manner. However, compound **13** reduced the α-syn oligomer formation at high micromolar concentration and was not moved to biological testing. Finally, BTA and 5-NBA were challenged with cell-based assays using M17D neuroblastoma cells expressing inclusion-prone αS-3K::YFP. Only 5-NBA successfully reduced inclusions in a dose-dependent manner and without affecting cell confluence. Based on our data, we propose that the 5-NBA represents an important building block for designing additional early-stage aggregation inhibitors.

## Supporting Information Available

Additional data pertaining to the kinetics of aggregation of TTR fragments, the antiaggregation activities of BTA and 5-NBA derivatives, the photoinduced cross-linking of unmodified proteins (PICUPs), and the compound characterization (PDF)

## Notes

All information in this article is provided for academic and scientific purposes only. The authors declare no competing financial interest.

## Abbreviations

Aβ: amyloid-β
AD: Alzheimer’s disease
BTA: 4-(benzo[*d*]thiazol-2-yl)aniline
dox: doxycycline
IAPP: islet amyloid polypeptide
5-NBA: 5-nitro-1,2-benzothiazol-3-amine
PD: Parkinson’s disease
PICUPs: photoinduced cross-linking of unmodified proteins
RPD: relative pixel density
SEM: standard error of the mean
α-syn: α-synuclein
TEM: transmission electron microscopy
ThT: thioflavin T
TTR: transthyretin
tau: tubulin associate unit
