# Supplementary material for: 5-Nitro-1,2-benzothiazol-3-amine and N-Ethyl-1-[(ethylcarbamoyl)(5-nitro-1,2-benzothiazol-3-yl)amino]formamide Modulate α-Synuclein and Tau Aggregation
Source: ACS Omega. 2023 May 23;8(22):20102–15. doi: 10.1021/acsomega.3c02668 (PMC10249125; doi:10.1021/acsomega.3c02668)
Supplement: Supplementary file 1 — ao3c02668_si_001.pdf [file ao3c02668_si_001.pdf]

5-Nitro-1,2-benzothiazol-3-amine and *N*-ethyl-1-[(ethylcarbamoyl)(5-nitro-1,2-benzothiazol-3-yl)amino]formamide modulate alpha-synuclein and tau aggregation

Eduardo Ramirez[1], *Susantha K. Ganegamage*[1], *Ahmed A. Elbatrawy*[1], *Heba Alnakhala*[2], *Kazuma Shimanaka*[2], *Arati Tripathi*[2], *Sehong Min*[3], *Jean-Christophe Rochet*[3], *Ulf Dettmer*[2], *Jessica S. Fortin*[1*]

[1]Department of Basic Medical Sciences, College of Veterinary Medicine, Purdue University, 625 Harrison Street, West Lafayette, Indiana, USA; [2]Ann Romney Center for Neurologic Diseases, Department of Neurology, Brigham and Women's Hospital and Harvard Medical School, Boston, MA 02115, USA; [3]Department of Medicinal Chemistry and Molecular Pharmacology, College of Pharmacy, Purdue University, USA

Supportive information (SI)

Prediction of the aggregation propensity by in silico analysis. The human TTR amino acid sequences (127 amino acids) were obtained from NCBI. An aggregation score for each region of the human TTR as depicted in **Figure S1** was obtained by *in silico* analysis using the bioinformatics program Tango.[1-3] This bioinformatic program is based on the physicochemical principles of beta-sheet formation, extended by the assumption that the core regions of an aggregate are fully buried. TANGO also correctly predicts pathogenic vs protective mutations of the Alzheimer's β-peptide, human lysozyme, and transthyretin. Furthermore, this program can discriminate between β-sheet propensity and aggregation. We obtained the aggregation (Agg) scores of the different regions of TTR with the TANGO program.

$TTR_{81\text{-}127}$ exhibits a high aggregation score. Bioinformatic analysis of human TTR amino acid regions indicated that the C-terminal portion 87-127 (ultimately cleaved) exhibits a high propensity to aggregate in comparison with the N-terminal portion of the protein (**Figure S1**).[4] Within the protein of 127 amino acids, the Agg score of the proximal segment 26-75 was null, thus representing the least prone-to-aggregate region of the protein (**Figure S1**). The portions 1-25 and 76-100 received Agg scores of 49 and 31, respectively. Such is indicative of a poor ability to aggregate (**Figure S1**). This confirms that $TTR_{81\text{-}127}$ is the most prone-to-aggregate region.

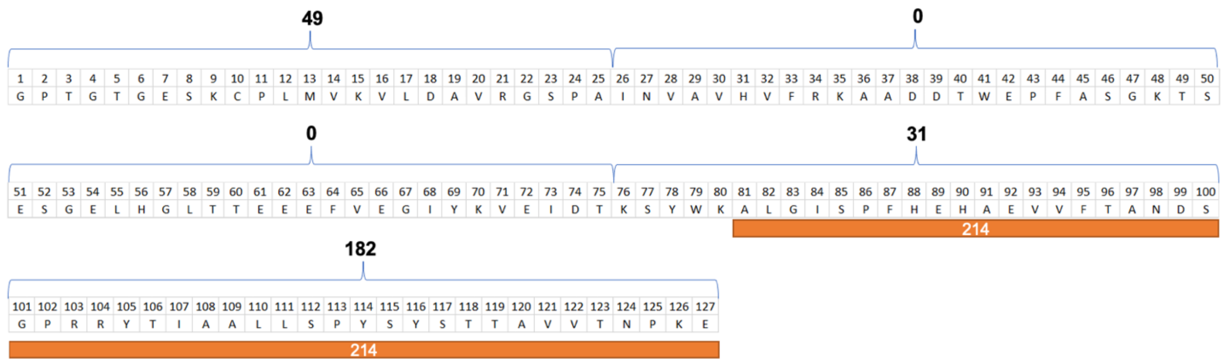

Figure S1. Propensity of aggregation of human transthyretin (TTR) regions. Each of the five segments of the human TTR sequence are labelled with a numerical aggregation (Agg) score. The highest Agg score of 214 resulted from the pathogenic C-terminal region of fragments 81-127 (represented by the orange bar). The amino acids 26-75 exhibited a null propensity of aggregation; hence, these sectors received an Agg score of 0. Sequences 1-25 and 76-100 amino acids were mildly prone to aggregation, as indicated by the Agg scores of 49 and 31, respectively. All Agg scores were obtained with the Tango program.

Prior to our study with different compounds, we evaluated the aggregation propensity of various TTR fragments using the Thioflavin T (ThT) assay. Fragments exhibiting a null score failed to form fibrils as confirmed by the low fluorescence intensity obtained during the kinetics of aggregation. $TTR_{105-125}$, $TTR_{101-125}$, and TTR_{81-127}, were the most prone-to-aggregate fragments as demonstrated by the emission of high fluorescence intensity (**Figure S2**).

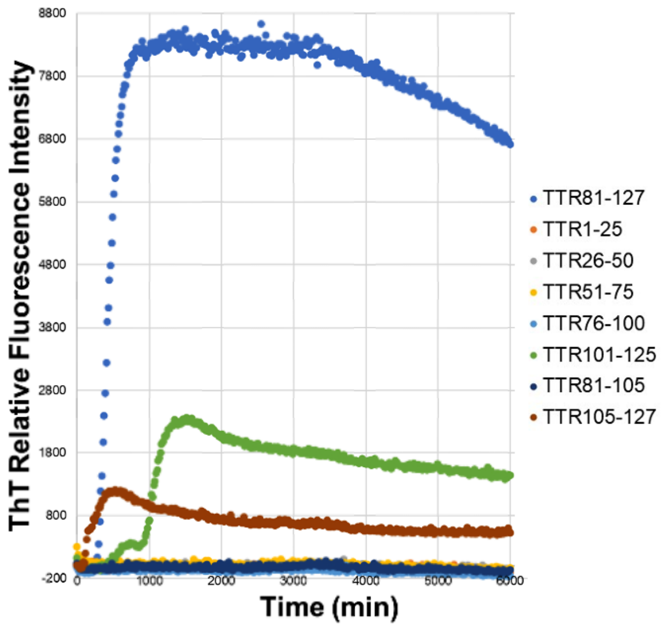

Figure S2. Transthyretin (TTR) fragments 81-127, 101-125, and 105-127 exhibit the sigmoidal curve of prone-to-aggregate proteins. Thioflavin T (ThT) relative fluorescence intensity over time of each TTR fragment peptides. The high relative fluorescence intensity of TTR 81-127 (data points in blue) indicates a high aggregation propensity, especially when compared to the lower curves of the other TTR regions.

4-(benzo[*d*]thiazol-2-yl)aniline (BTA) is a potent inhibitor of TTR_{81-127} fibrils as monitored by ThT fluorescence assays. We decided to evaluate the effect of BTA and derivatives on the two fragments resulting in the highest fluorescence intensity and sigmoidal curves: TTR_{81-127} and $TTR_{101-125}$. We relied on the ThT aggregation kinetics to assess fibril formation in presence of DMSO (0.25%, control; CTRL), resveratrol (positive control), and BTA at 100 µM. Resveratrol (polyphenol) was used as a control, previously proven to be a general inhibitor of fibril formation.[5] Resveratrol is a known inhibitor of human islet amyloid polypeptide fibril formation.[6] By using resveratrol as a positive control, a baseline for inhibition of aggregation potential was established, allowing for the comparison with BTA to determine fibril aggregation inhibition. Fibrils treated with BTA exhibited a significantly smaller fluorescent value when compared to the neutral control and positive control of resveratrol. The kinetic curve for the BTA-treated TTR fragment was lower compared to the curves of the control and resveratrol-treated TTR fragment (**Figure S3A**). The higher maximum fluorescence intensities measured in the control and positive control are

indicative of increased fibril formation and continuation (**Figure S3B**). The percentages of fibril formation measured at the plateau phase of the kinetics of fibril formation obtained with control and BTA and its derivatives were compared. With the ThT fluorescence of BTA measured to be 4.1%, the fibril inhibition is much greater than that of resveratrol (48.2% fluorescence) and the control (100% fluorescence) (**Figure S3B**). BTA demonstrated a ten-fold increase in reduction of fluorescence intensity than the positive control, resveratrol, in inhibiting fibril formation. While not significant, the time required for the BTA treated sample to elongate into fibrils (lag time) was less than that of both the neutral and positive control (**Figure S3C**).

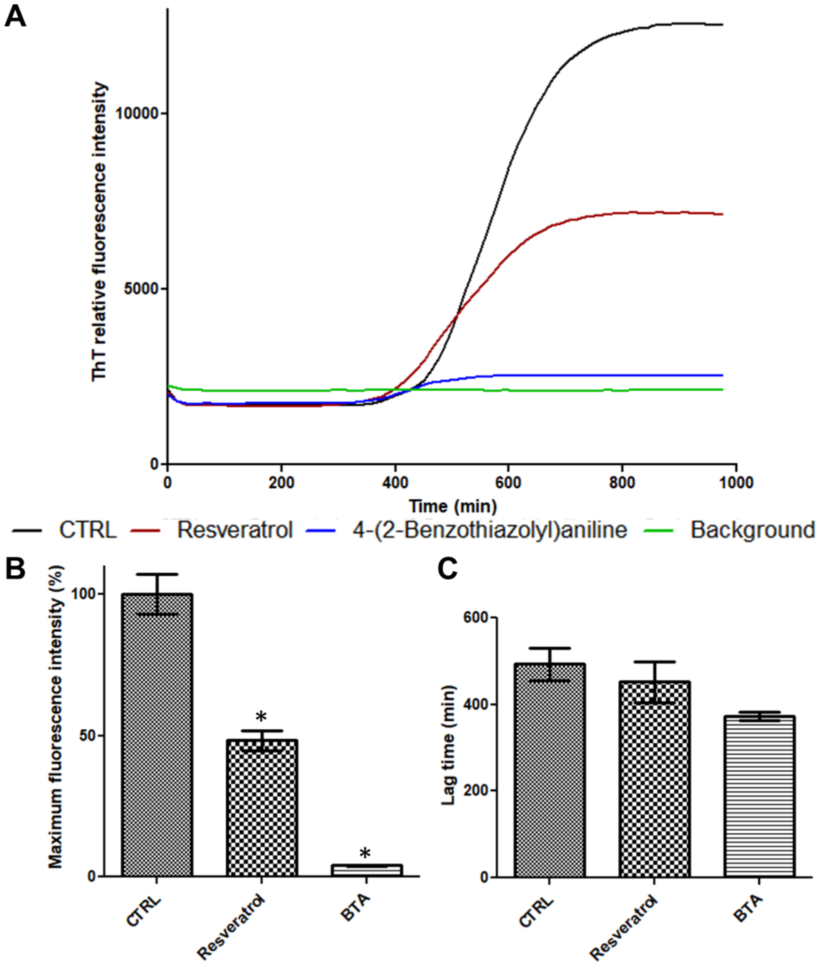

Figure S3. 4-(benzo[*d*]thiazol-2-yl)aniline (BTA) reduces significantly the $TTR_{81\text{-}127}$ fibril formation, without delaying the lag time. (**A**) Kinetic curves of $TTR_{81\text{-}127}$ fibril formation as monitored by Thioflavin T (ThT) fluorescence emission in presence of resveratrol (positive control) and BTA at 100 µM. (**B**) Histogram representing the percentage of $TTR_{81\text{-}127}$ fibril

formation at plateau phase. Each bar represents the average of the last five fluorescence values obtained and the error bar is the standard error of the mean (SEM) ($p < 0.05$). (**C**) The time required for the elongation into fibrils (lag time) for each condition was plotted in the histogram (mean and SEM). No significant changes were noted by One-way analysis of variance or Dunnett's multiple comparison test.

To confirm the anti-fibrillization effect of BTA and compound **2**, TEM was performed on 60 µM TTR_{81-127} and $TTR_{101-125}$ fragments treated with 1.5% DMSO control, 600 µM of compounds **2**, compound **3**, resveratrol, and BTA (molar ratio 1:10) for 40 hours in sodium acetate buffer, pH 4 at 37 °C (**Figure S4**). Dense mats of fibrils were obtained with the control and plaques were also observed with compound **3** and resveratrol treatment. Fibrils were substantially reduced on the grids prepared with both TTR_{81-127} and $TTR_{101-125}$ fragments treated with compound **2** and BTA.

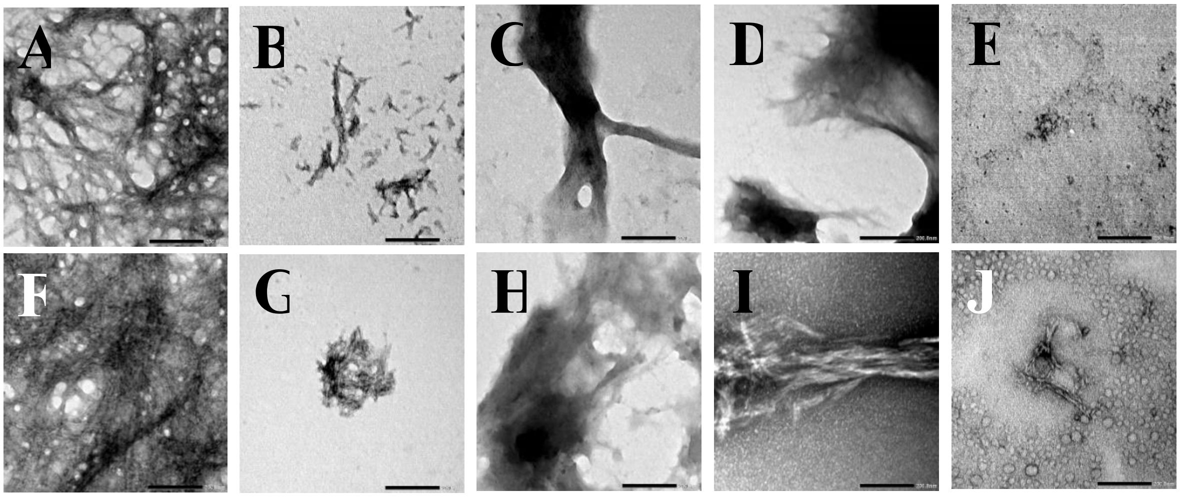

Figure S4. Compound **2** and BTA diminish the fibril formation of both fragments TTR_{81-127} and $TTR_{101-125}$, as confirmed by transmission electron microgram (TEM). TTR_{81-127} and $TTR_{101-125}$ fragments were incubated at 600 µM with each compound at a concentration of 60 µM (molar ratio 1:10) for 40 hours in sodium acetate buffer, at pH 4 and 37ºC. Microphotographs were acquired at magnification of 40K. **A)** TTR_{81-127} incubated with 1.5% DMSO control. **B)** TTR_{81-127} incubated with 100 µM of compound **2**. **C)** TTR_{81-127} incubated with 100 µM of compound **3**. **D)** TTR_{81-127} incubated with 100 µM of Resveratrol. **E)** TTR_{81-127} incubated with 100 µM of BTA. **F)** $TTR_{101-125}$ incubated with 1.5% DMSO control. **G)** $TTR_{101-125}$ incubated with 100 µM of

compound **2**. **H)** TTR$_{101-125}$ incubated with 100 µM of compound **3**. **I)** TTR$_{101-125}$ incubated with 100 µM of Resveratrol. **J)** TTR$_{101-125}$ incubated with 100 µM of BTA. Scale bars = 200 nm.

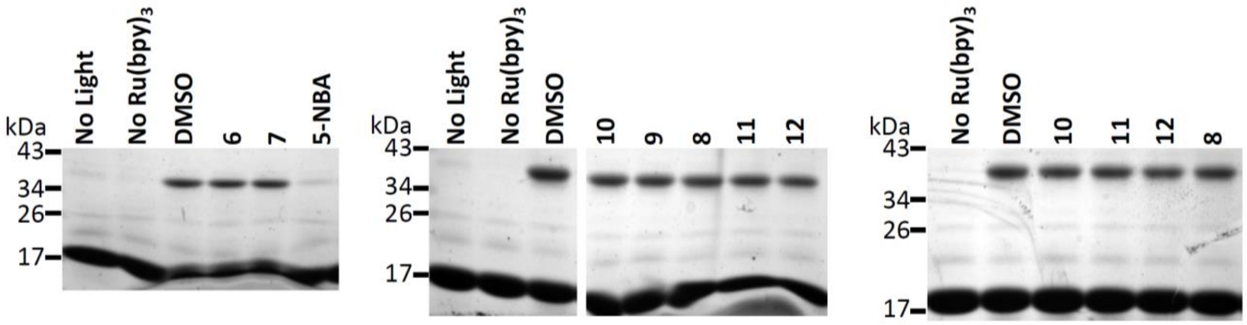

Figure S5. Additional Photo-induced Cross-linking of Unmodified Proteins (PICUP) assays performed with α-synuclein and compounds 6-12 listed in Table 1. Compounds **6-12** were tested at 50 µM and failed to inhibit substantially the formation of oligomers. 5-NBA refers to the 5-nitro-1,2-benzothiazol-3-amine and was utilized as a positive control. Additional controls for assay validation consist of no light and no cross-linking agent (no Ru(bpy)$_3$).

Characterization of compounds

NMR spectra have been acquired in deuterated DMSO. IR spectra have been obtained in solid state.

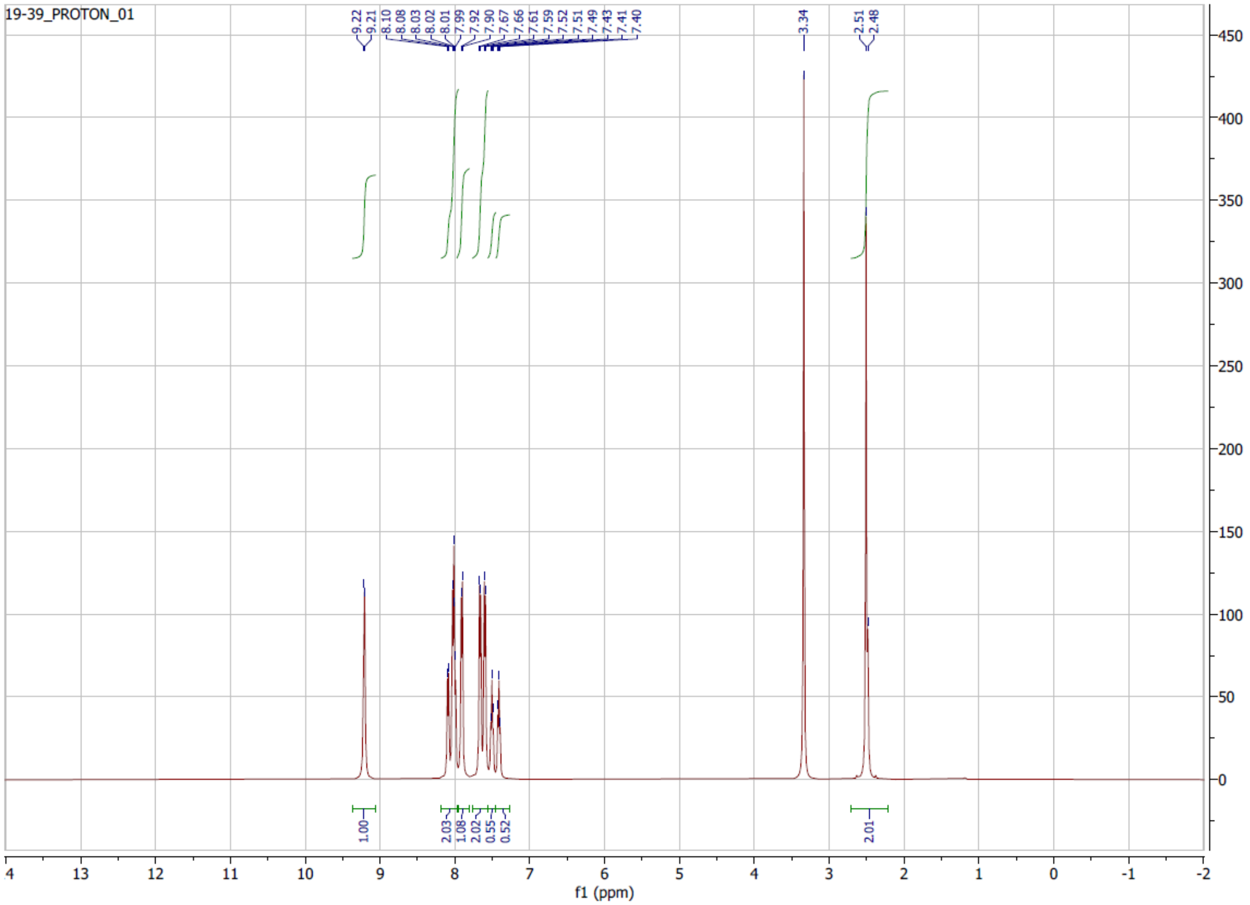

Figure S6. ^{1}H NMR for 1-(4-acetylphenyl)-3-[4-(1,3-benzothiazol-2-yl)phenyl]urea (**1**).

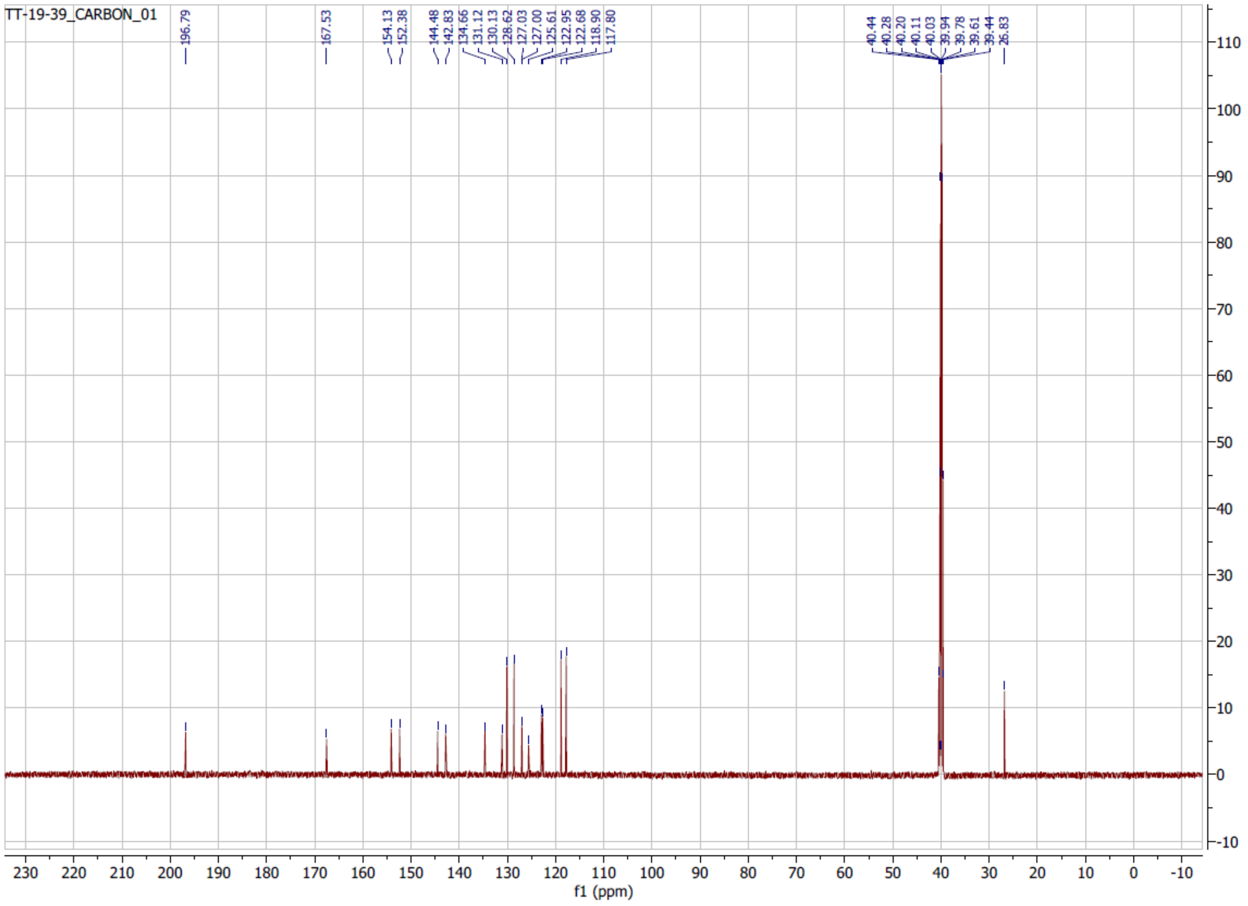

Figure S7. ^{13}C NMR for 1-(4-acetylphenyl)-3-[4-(1,3-benzothiazol-2-yl)phenyl]urea (**1**).

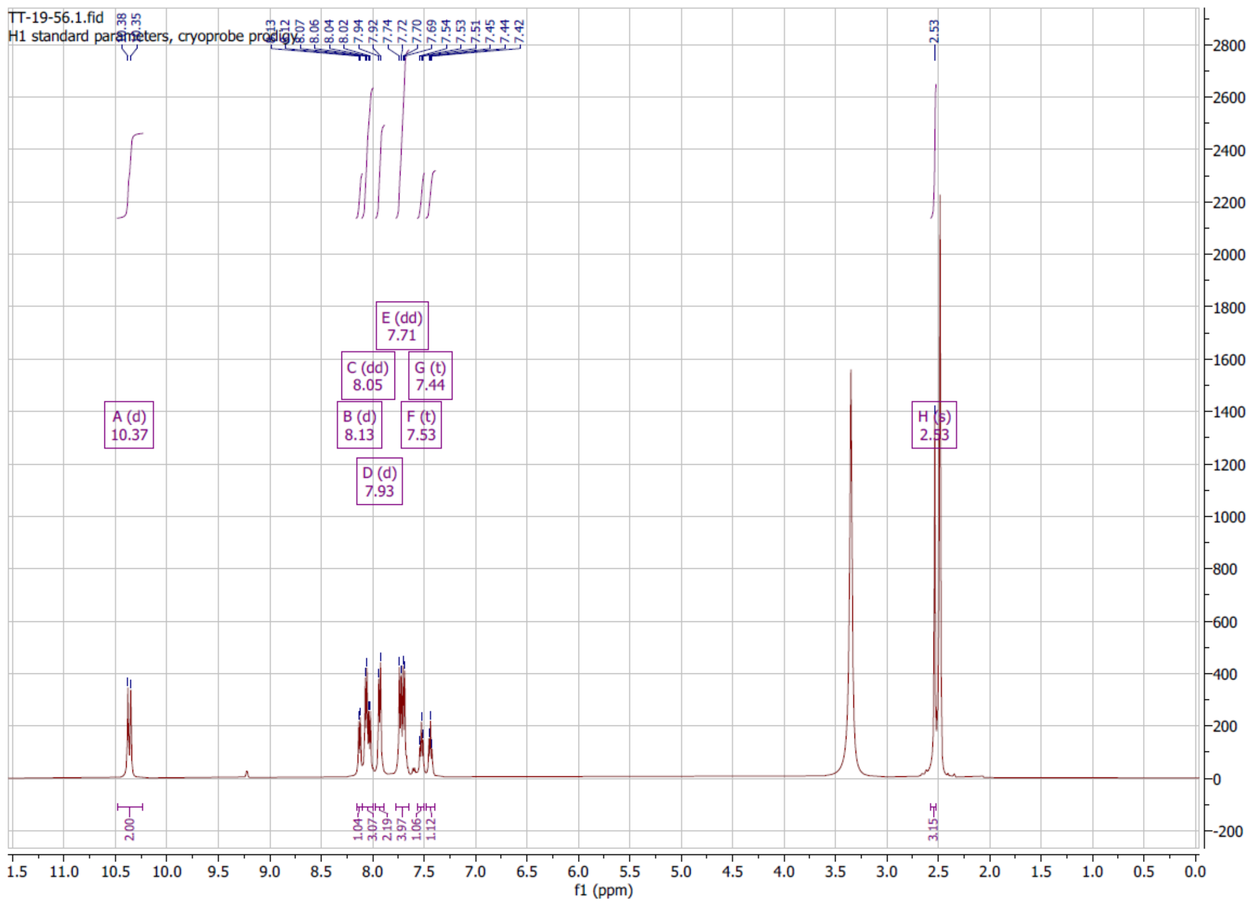

Figure S8. ^{1}H NMR for 1-(4-acetylphenyl)-3-[4-(1,3-benzothiazol-2-yl)phenyl]thiourea (**2**).

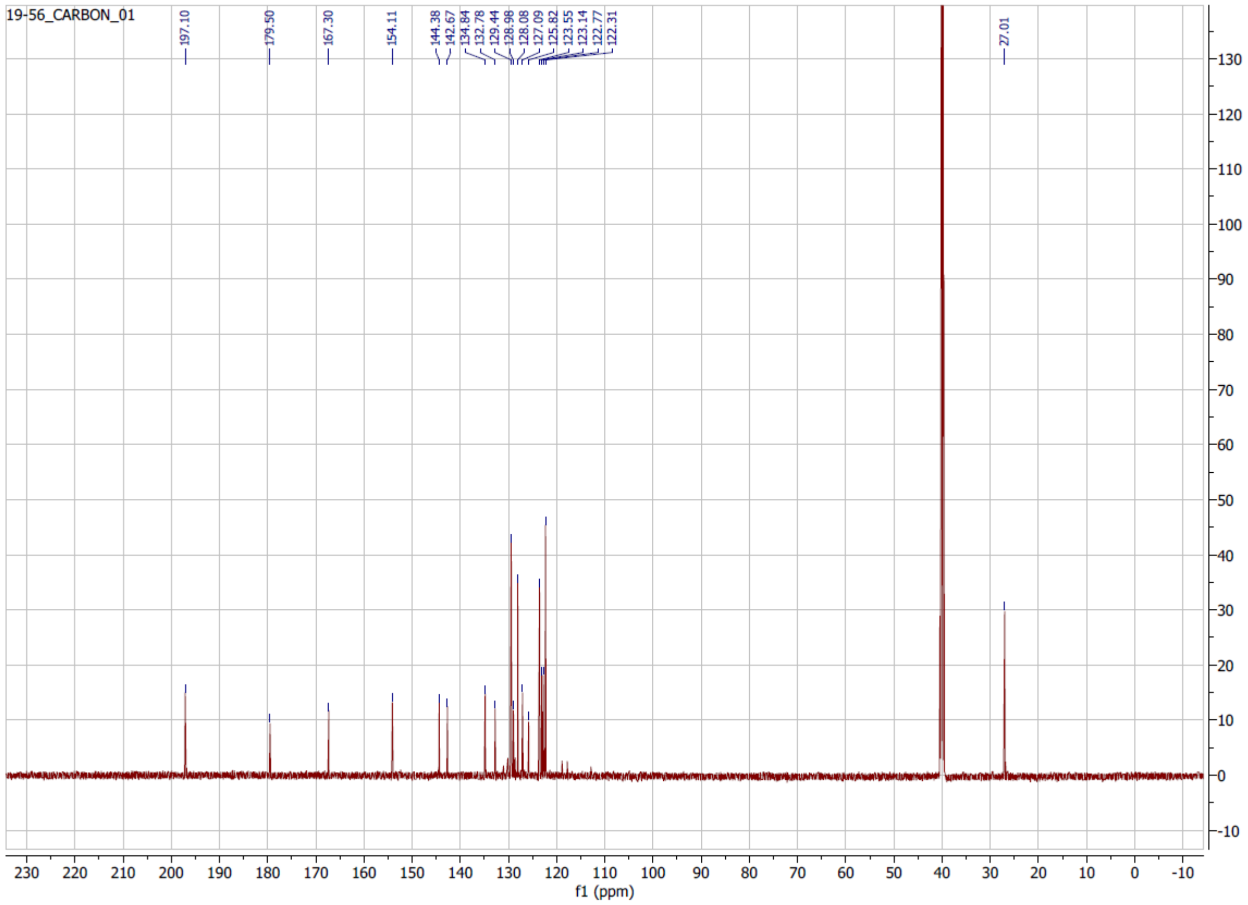

Figure S9. ^{13}C NMR for 1-(4-acetylphenyl)-3-[4-(1,3-benzothiazol-2-yl)phenyl]thiourea (**2**).

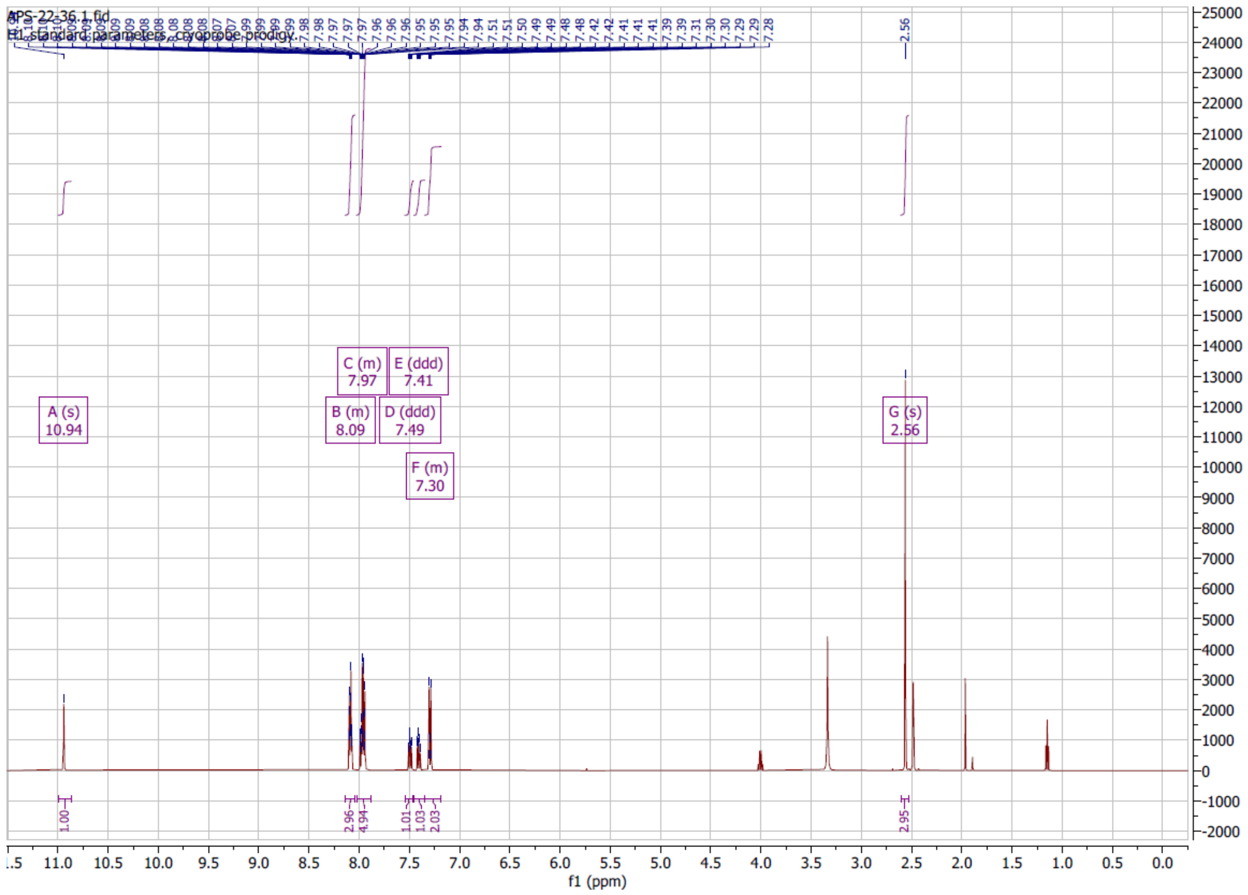

Figure S10. ^{1}H NMR for 4-acetyl-N-[4-(1,3-benzothiazol-2-yl)phenyl]benzene-1-sulfonamide (**3**).

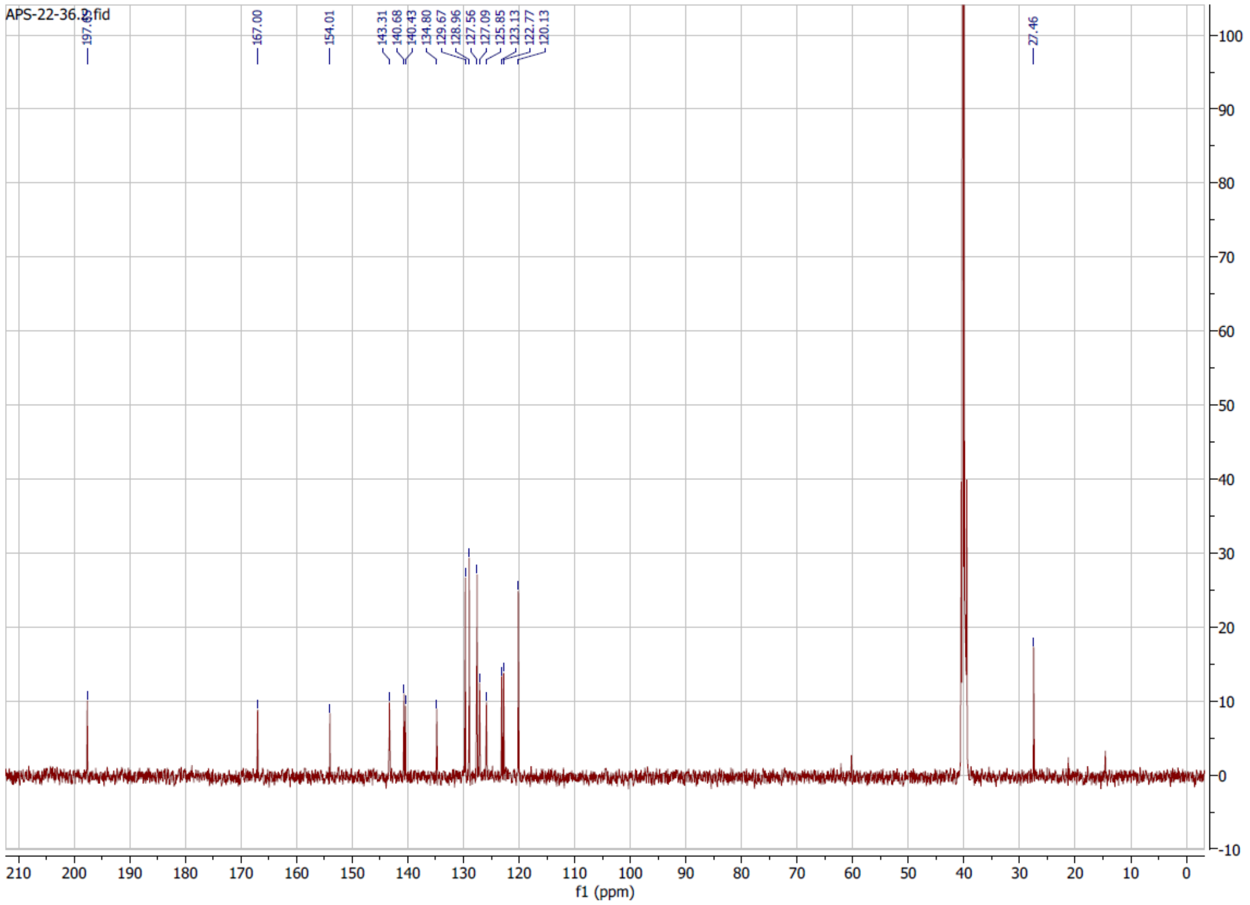

Figure S11. ^{13}C NMR for 4-acetyl-N-[4-(1,3-benzothiazol-2-yl)phenyl]benzene-1-sulfonamide (**3**).

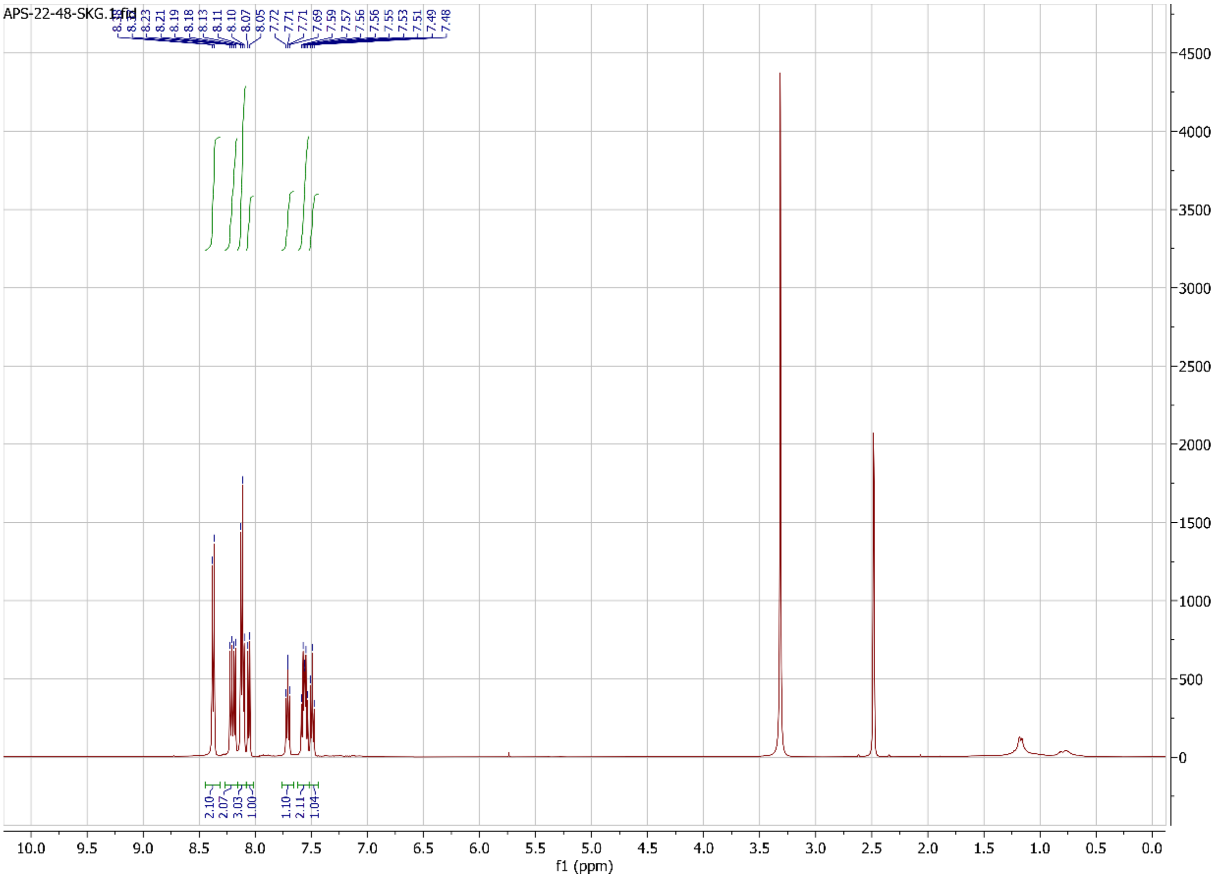

Figure S12. ^{1}H NMR for 1-[4-(1,3-benzothiazol-2-yl)phenyl]-1H-1,2,3-benzotriazole (**4**).

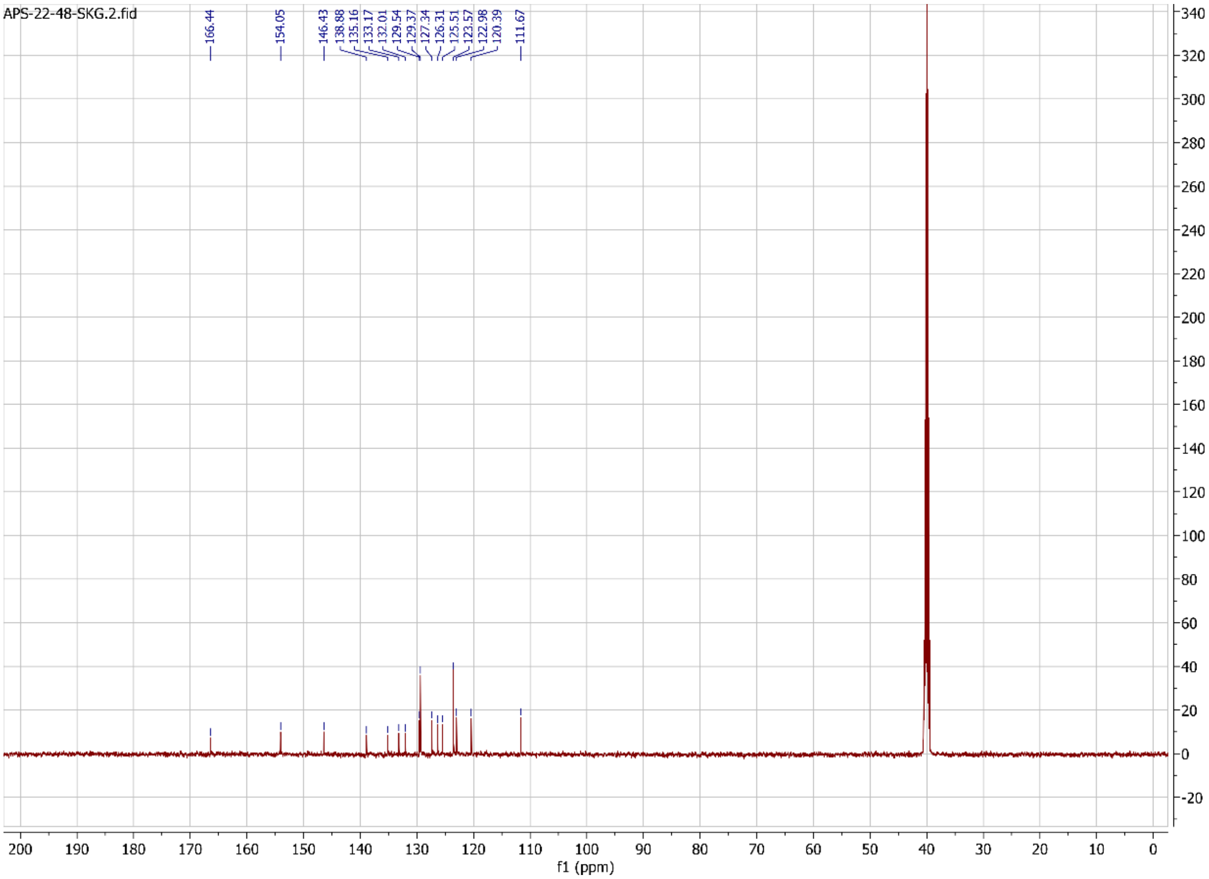

Figure S13. ^{13}C NMR for 1-[4-(1,3-benzothiazol-2-yl)phenyl]-1H-1,2,3-benzotriazole (**4**).

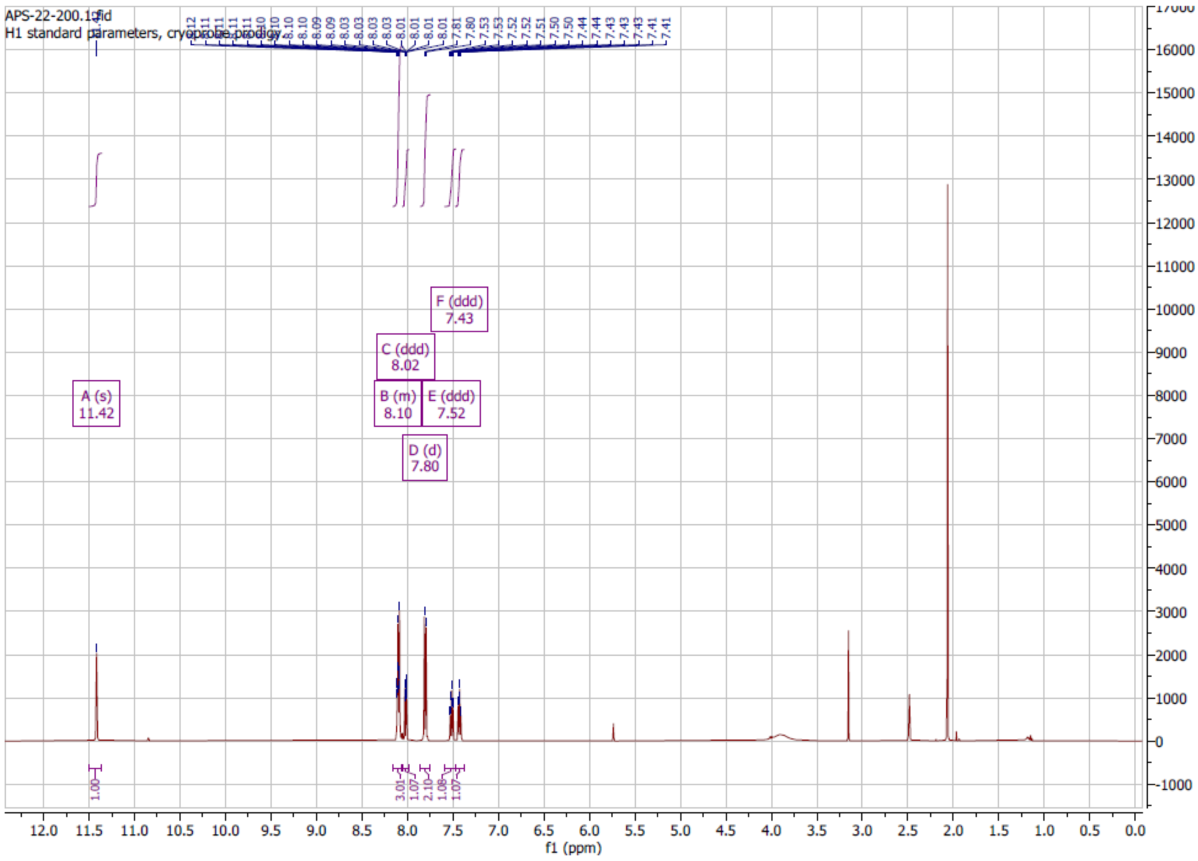

Figure S14. ¹H NMR for *N*-[4-(1,3-benzothiazol-2-yl)phenyl]-4,6-dichloro-1,3,5-triazin-2-amine (**5**).

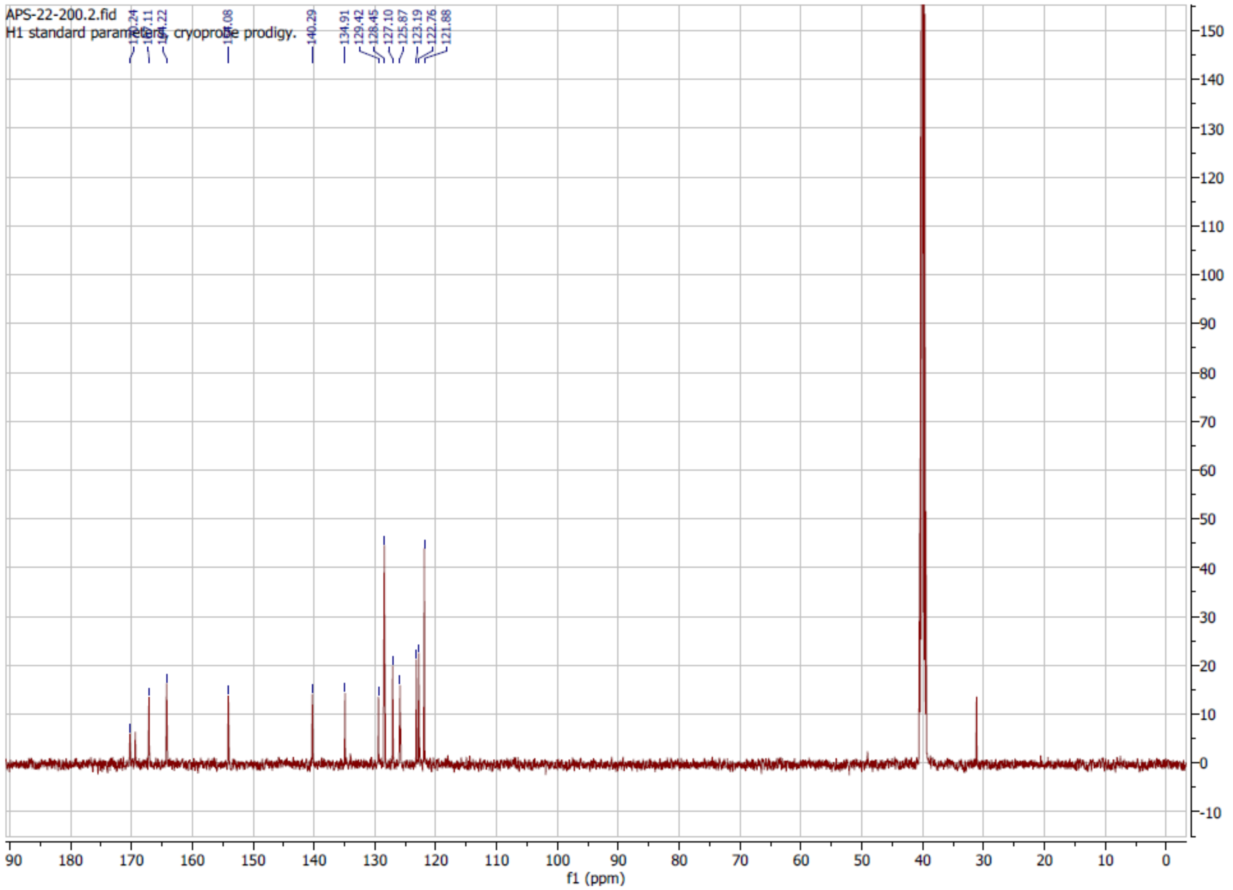

Figure S15. ^{13}C NMR for *N*-[4-(1,3-benzothiazol-2-yl)phenyl]-4,6-dichloro-1,3,5-triazin-2-amine (**5**).

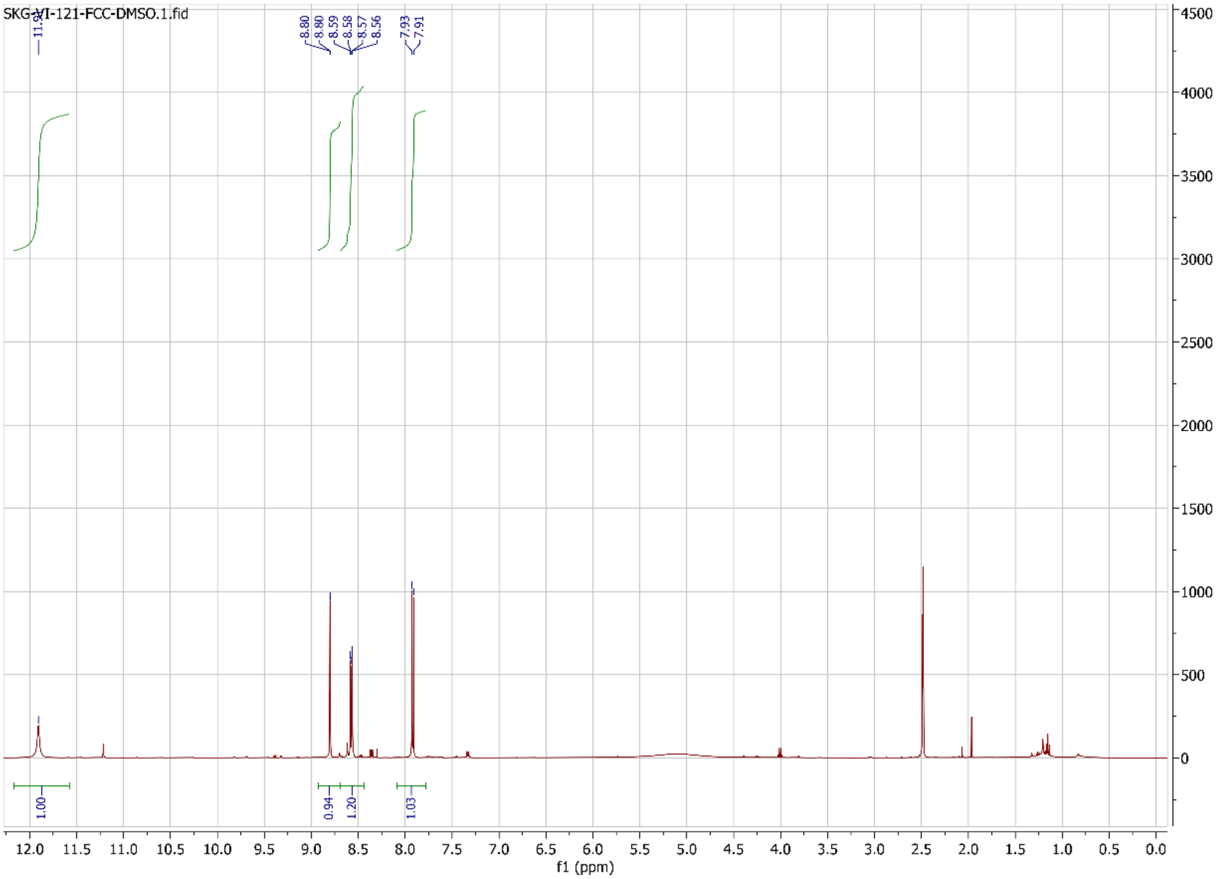

Figure S16. ^{1}H NMR for *N*-(4,6-dichloro-1,3,5-triazin-2-yl)-5-nitro-1,2-benzothiazol-3-amine (**6**).

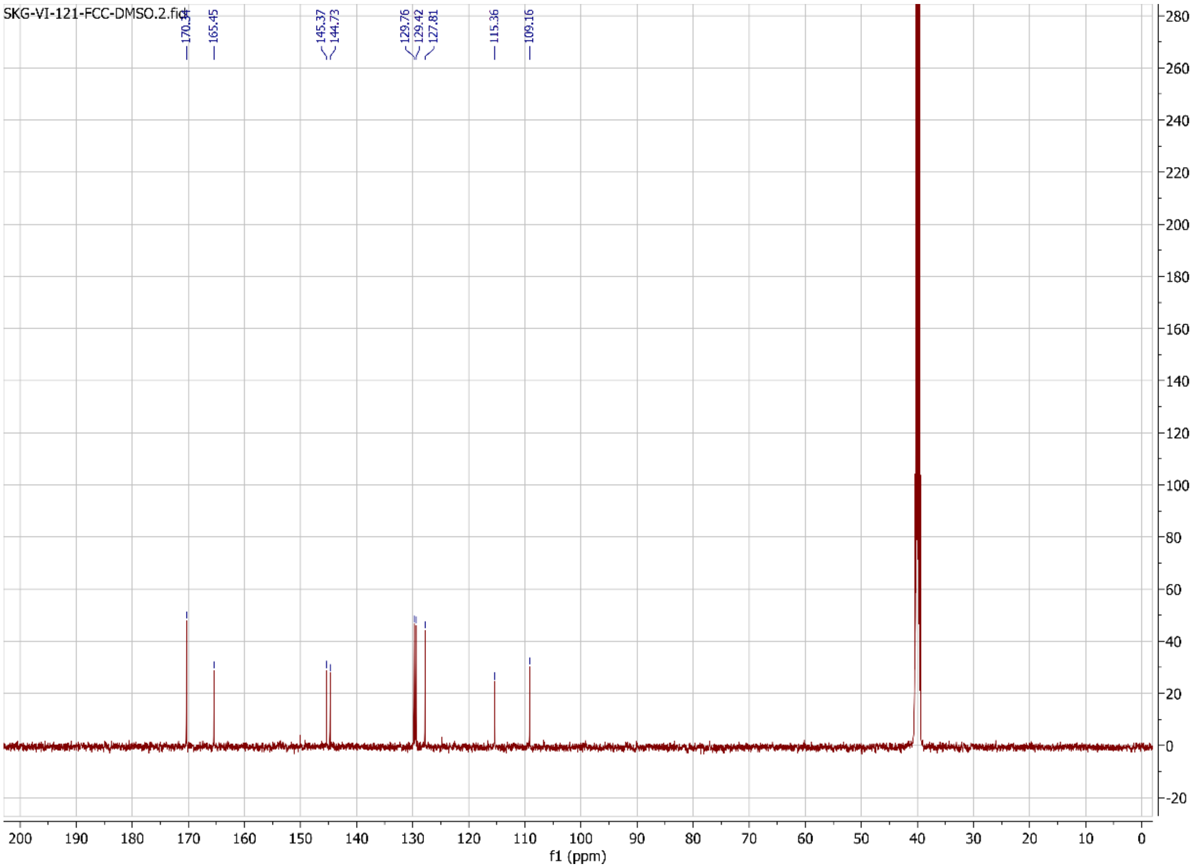

Figure S17. ^{13}C NMR for *N*-(4,6-dichloro-1,3,5-triazin-2-yl)-5-nitro-1,2-benzothiazol-3-amine (**6**).

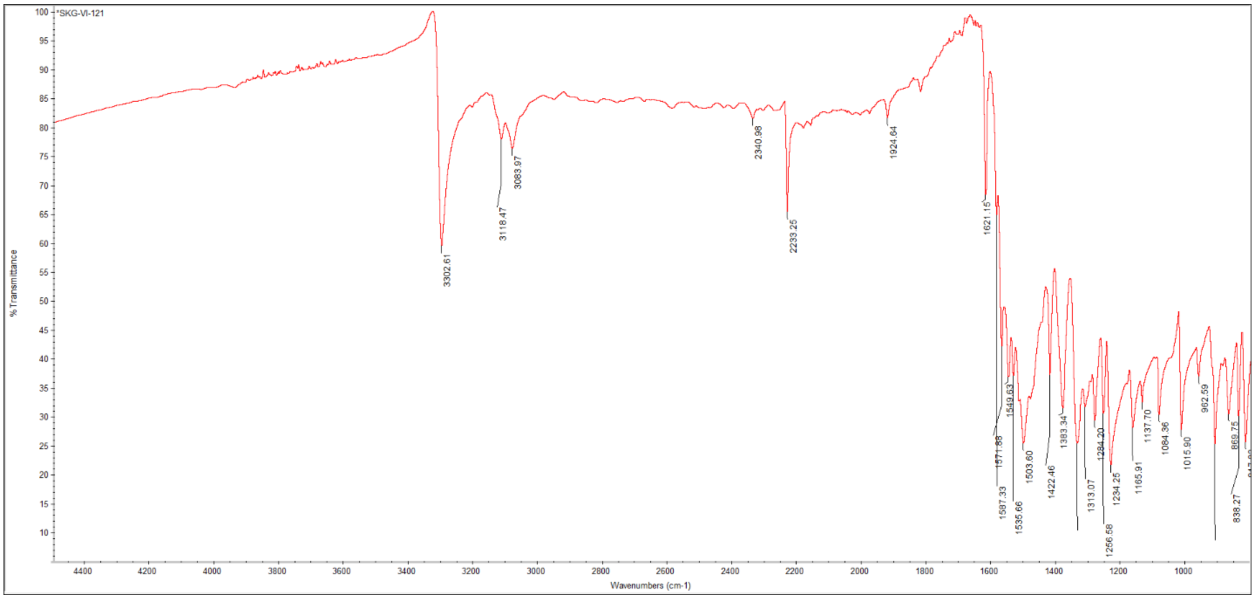

Figure S18. IR (solid) ν/cm^{-1} for *N*-(4,6-dichloro-1,3,5-triazin-2-yl)-5-nitro-1,2-benzothiazol-3-amine (**6**).

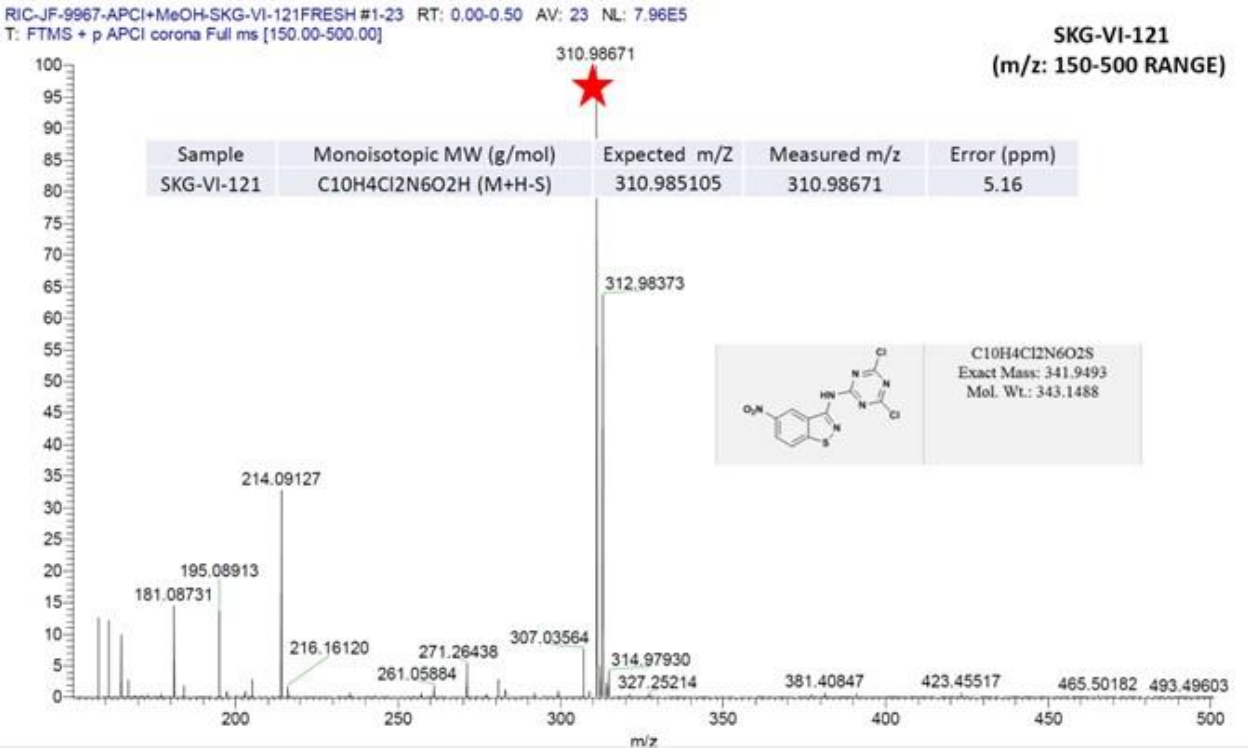

Figure S19. Mass spectrum (ionization in APCI) for *N*-(4,6-dichloro-1,3,5-triazin-2-yl)-5-nitro-1,2-benzothiazol-3-amine (**6**).

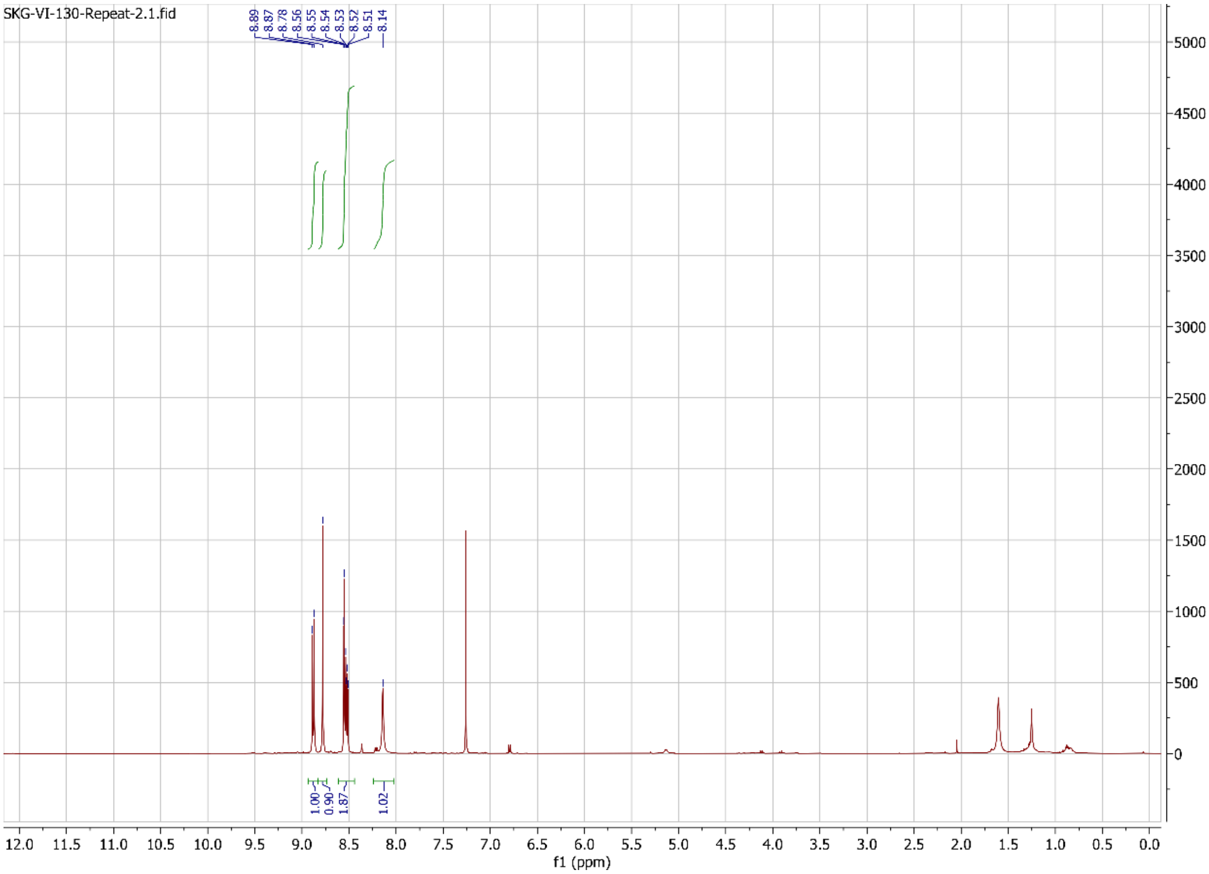

Figure S20. ^{1}H NMR for *N*-(4-chloro-1,3,5-triazin-2-yl)-5-nitro-1,2-benzothiazol-3-amine (**7**).

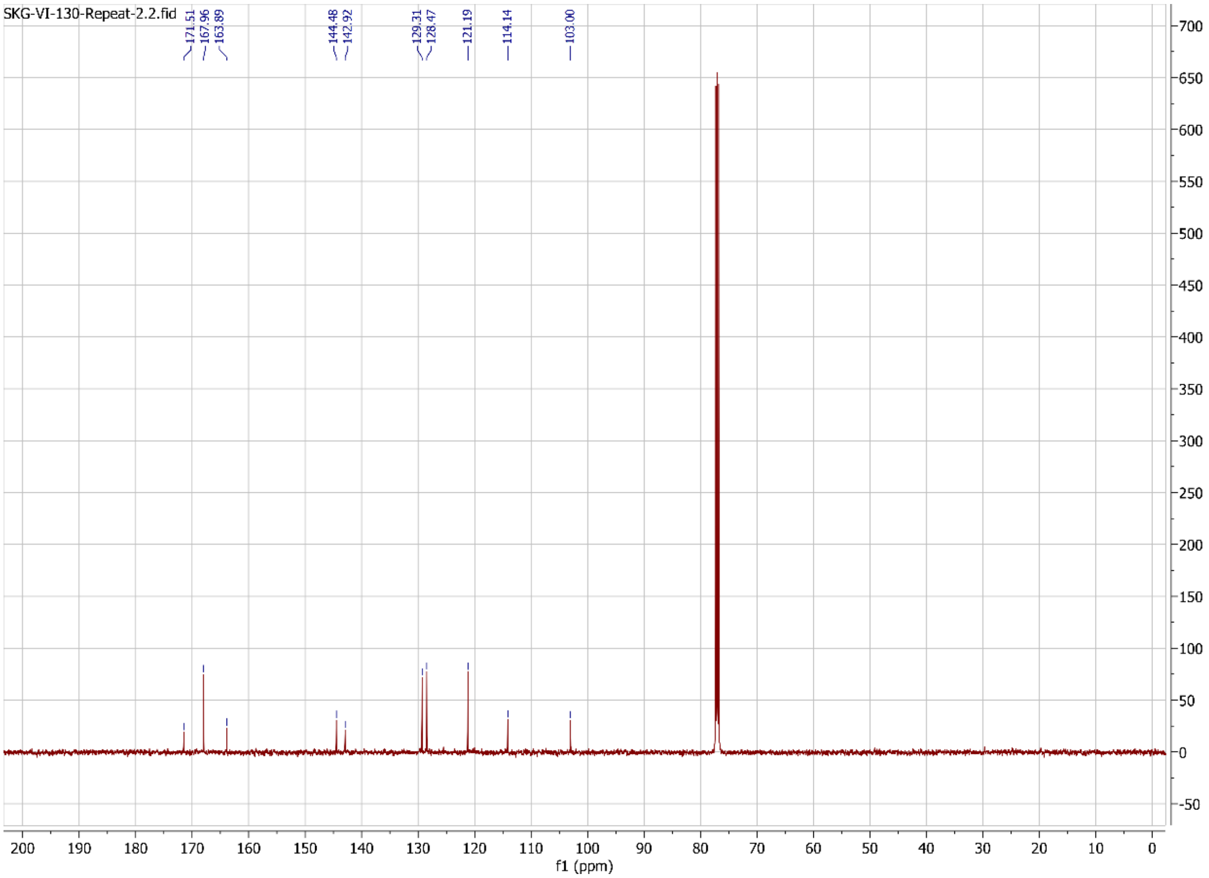

Figure S21. ^{13}C NMR for *N*-(4-chloro-1,3,5-triazin-2-yl)-5-nitro-1,2-benzothiazol-3-amine (**7**).

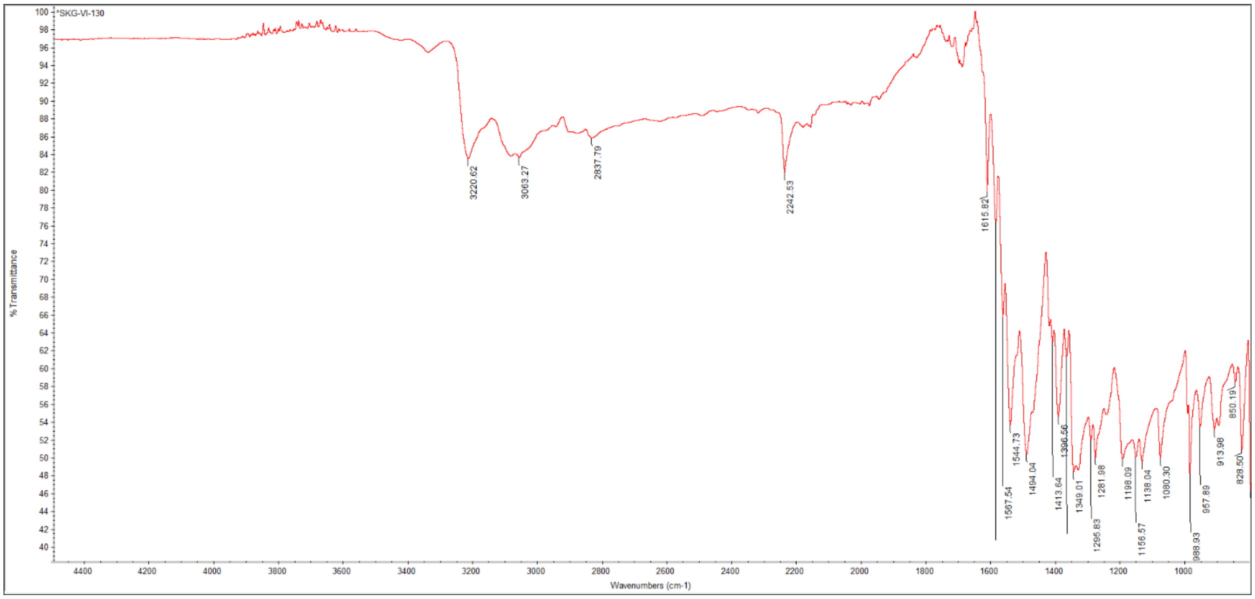

Figure S22. IR (solid) v/cm^{-1} for *N*-(4-chloro-1,3,5-triazin-2-yl)-5-nitro-1,2-benzothiazol-3-amine (**7**).

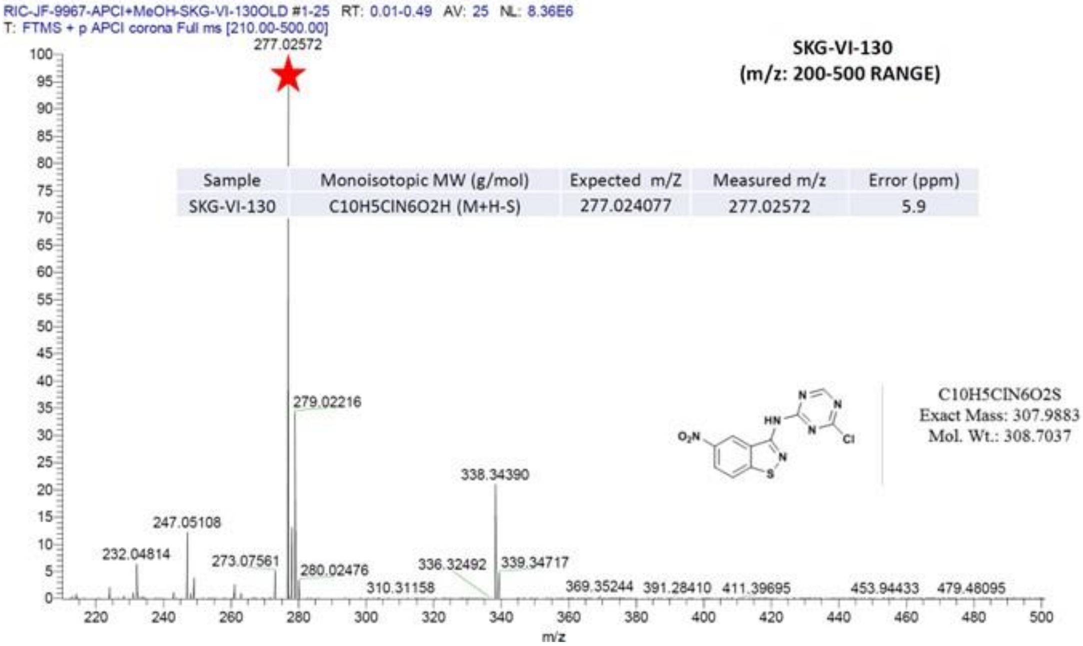

Figure S23. Mass spectrum (ionization in APCI) for *N*-(4-chloro-1,3,5-triazin-2-yl)-5-nitro-1,2-benzothiazol-3-amine (**7**).

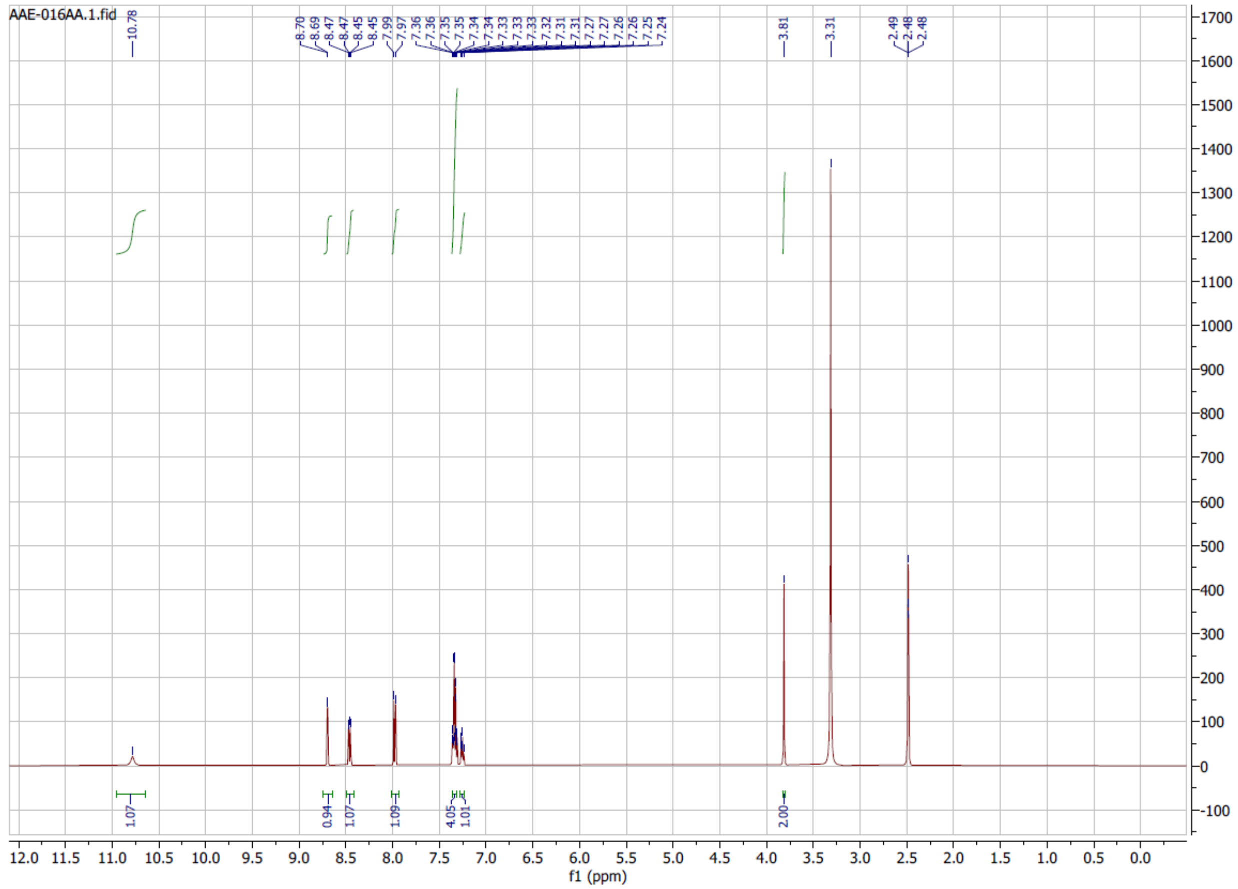

Figure S24. ^{1}H NMR for *N*-(5-nitro-1,2-benzothiazol-3-yl)-2-phenylacetamide (**8**).

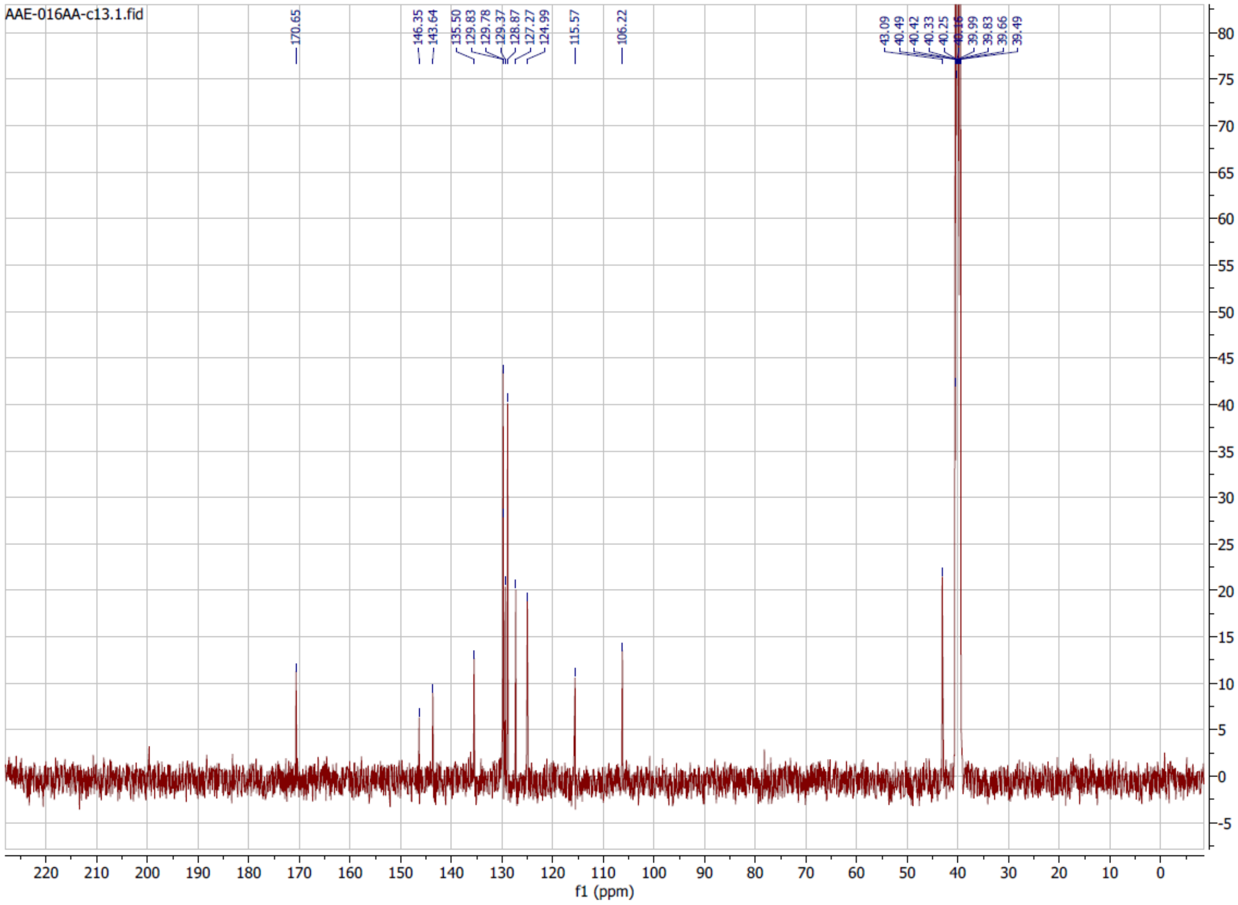

Figure S25. ^{13}C NMR for *N*-(5-nitro-1,2-benzothiazol-3-yl)-2-phenylacetamide (**8**).

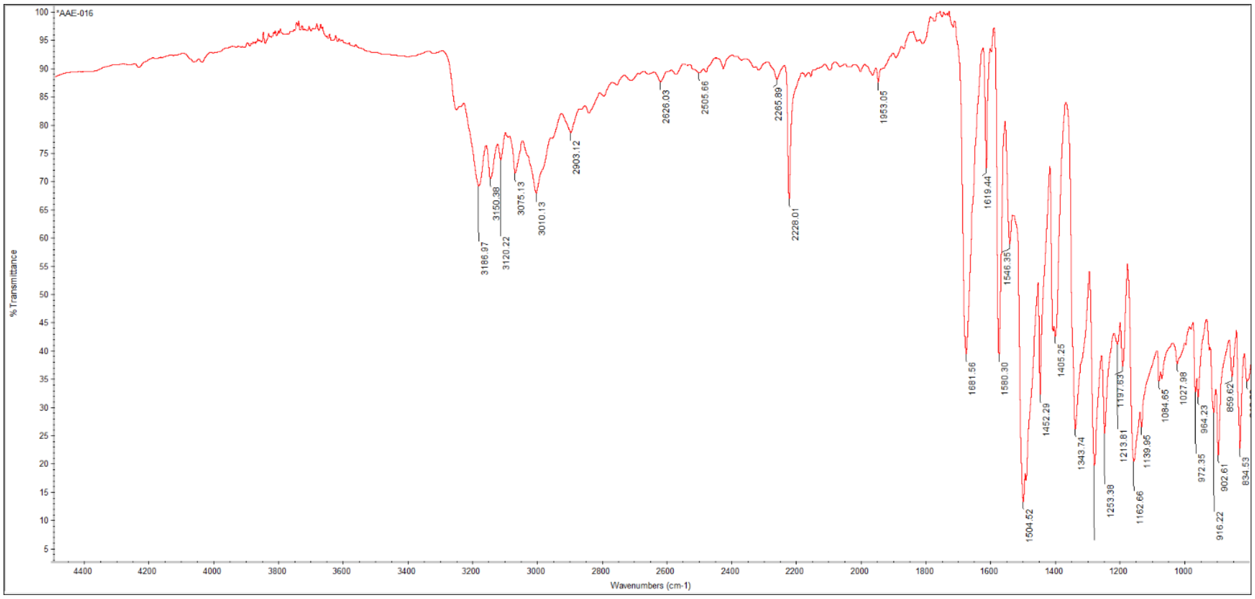

Figure S26. IR (solid) v/cm^{-1} for *N*-(5-nitro-1,2-benzothiazol-3-yl)-2-phenylacetamide (**8**).

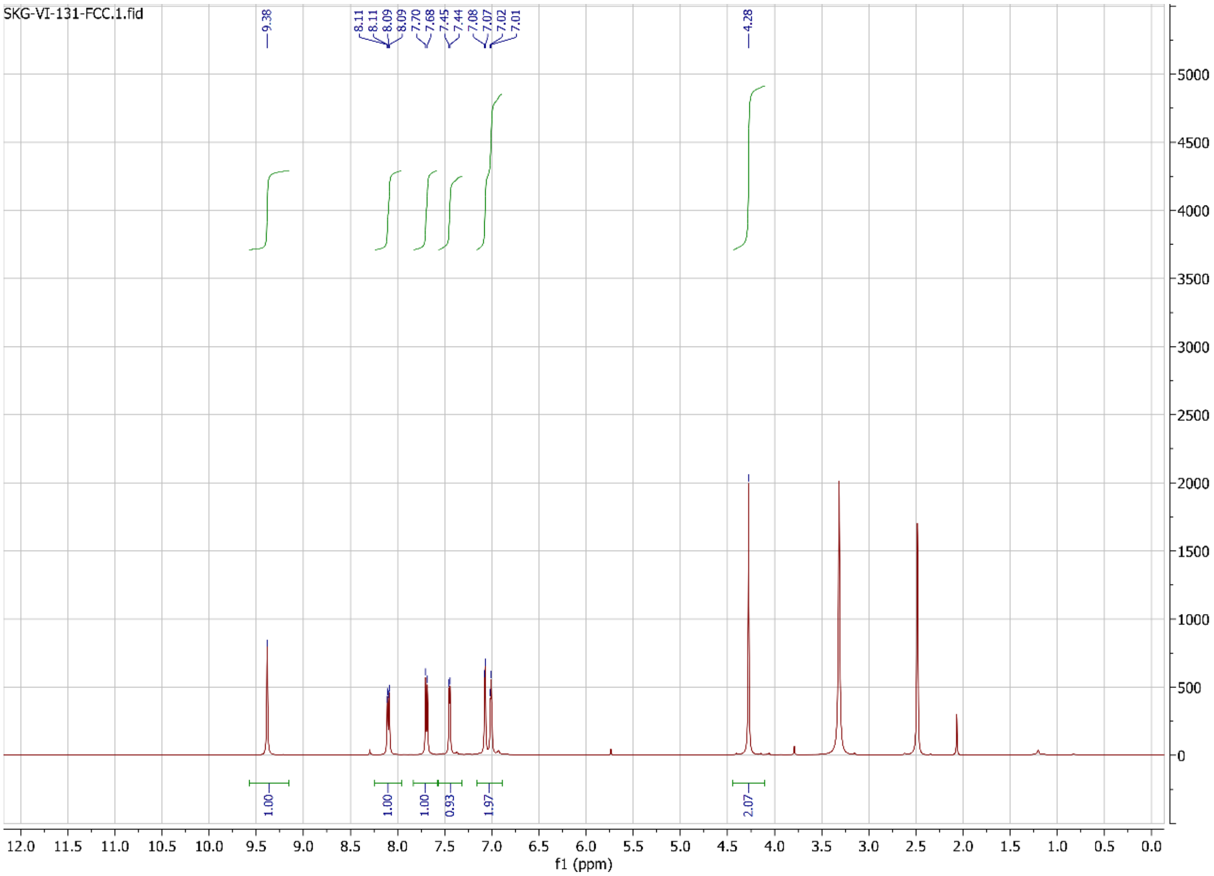

Figure S27. ^{1}H NMR for *N*-(5-nitro-1,2-benzothiazol-3-yl)-2-phenylacetamide (**9**).

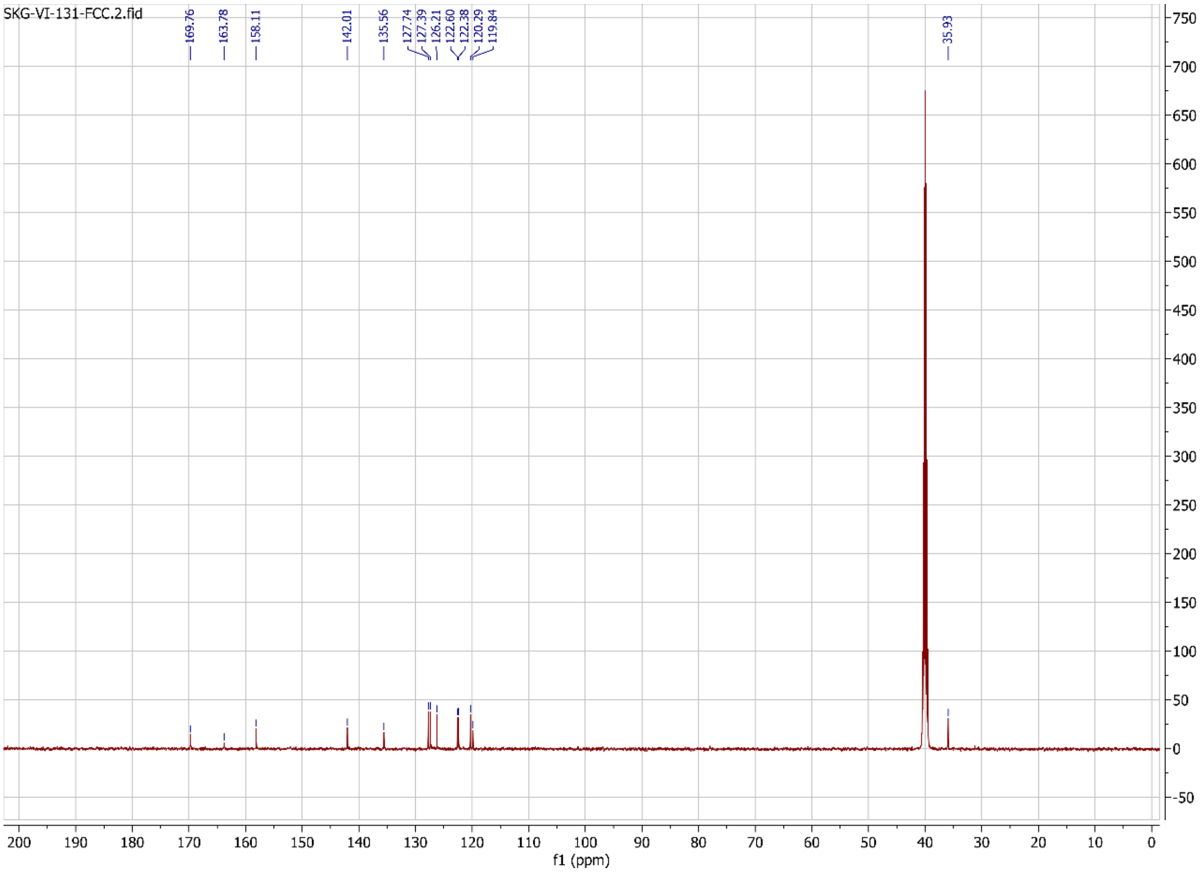

Figure S28. ^{13}C NMR for *N*-(5-nitro-1,2-benzothiazol-3-yl)-2-phenylacetamide (**9**).

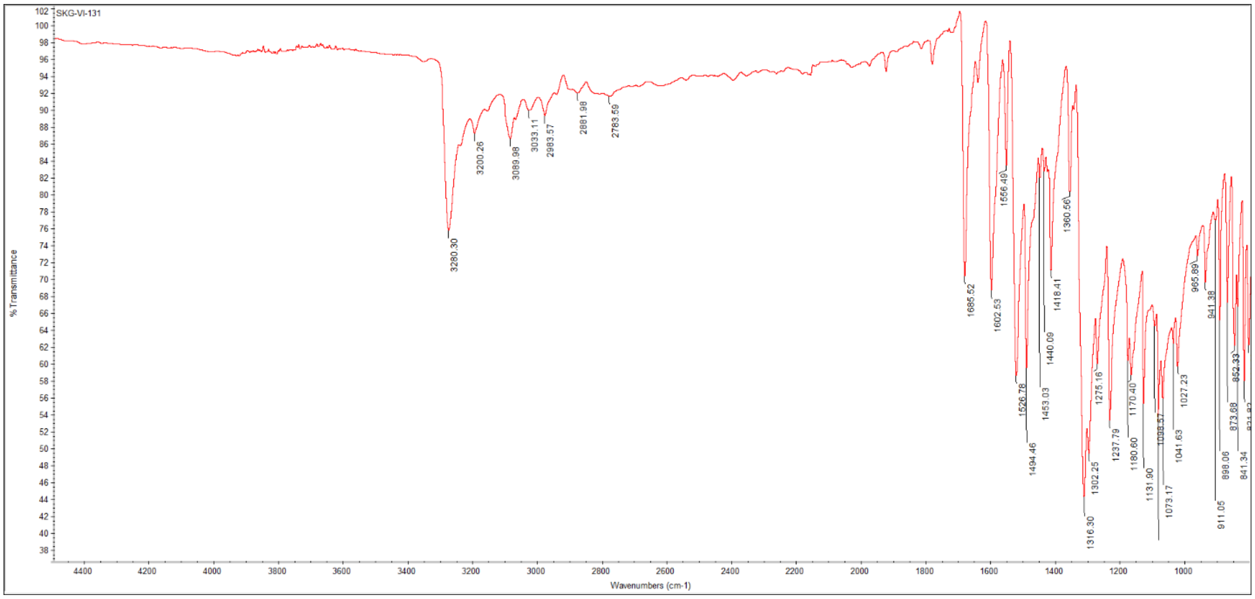

Figure S29. IR (solid) v/cm^{-1} for *N*-(5-nitro-1,2-benzothiazol-3-yl)-2-phenylacetamide (**9**).

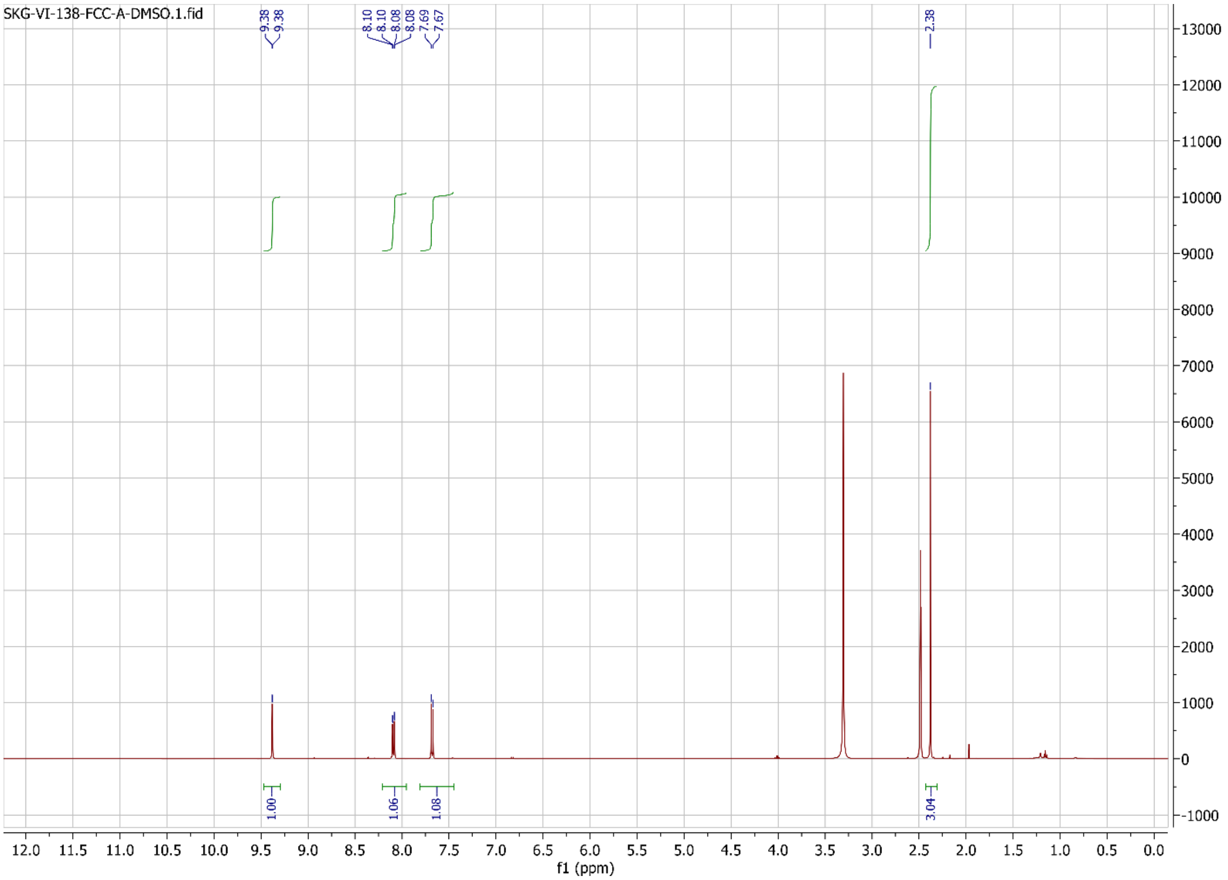

Figure S30. ^{1}H NMR for *N*-(5-nitro-1,2-benzothiazol-3-yl)acetamide (**10**).

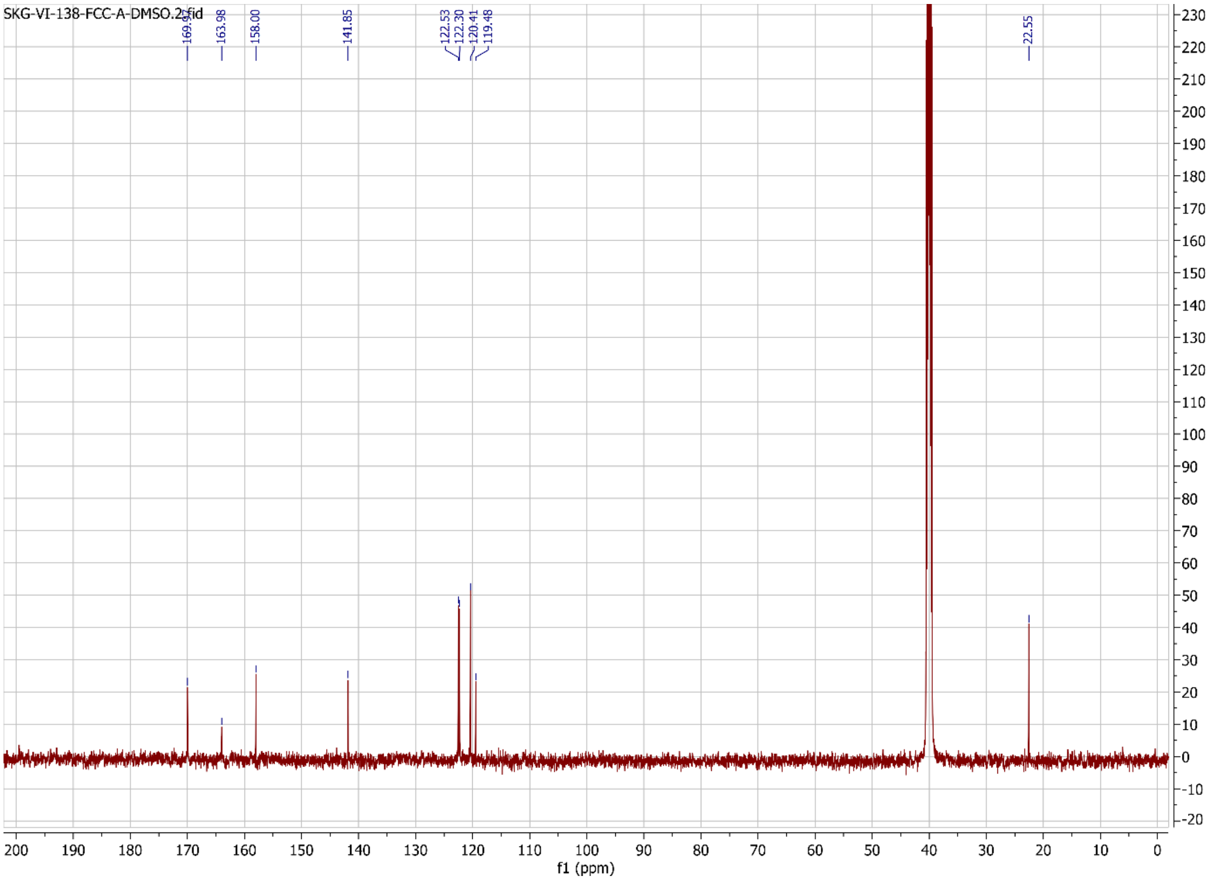

Figure S31. ^{13}C NMR for *N*-(5-nitro-1,2-benzothiazol-3-yl)acetamide (**10**).

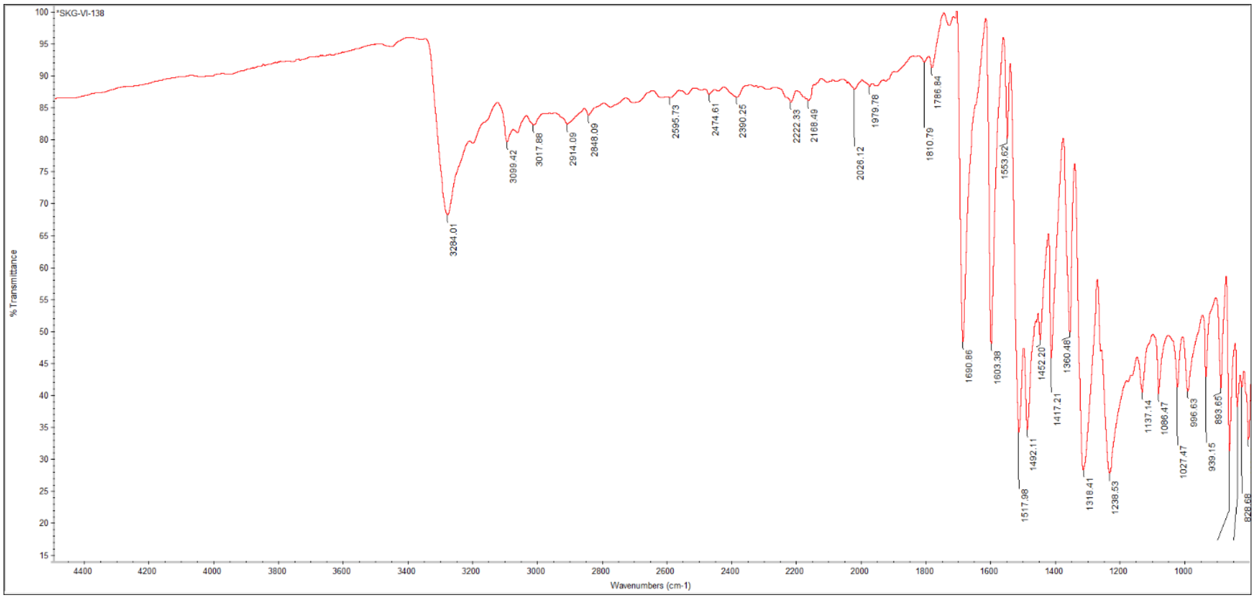

Figure S32. IR (solid) v/cm^{-1} for *N*-(5-nitro-1,2-benzothiazol-3-yl)acetamide (**10**).

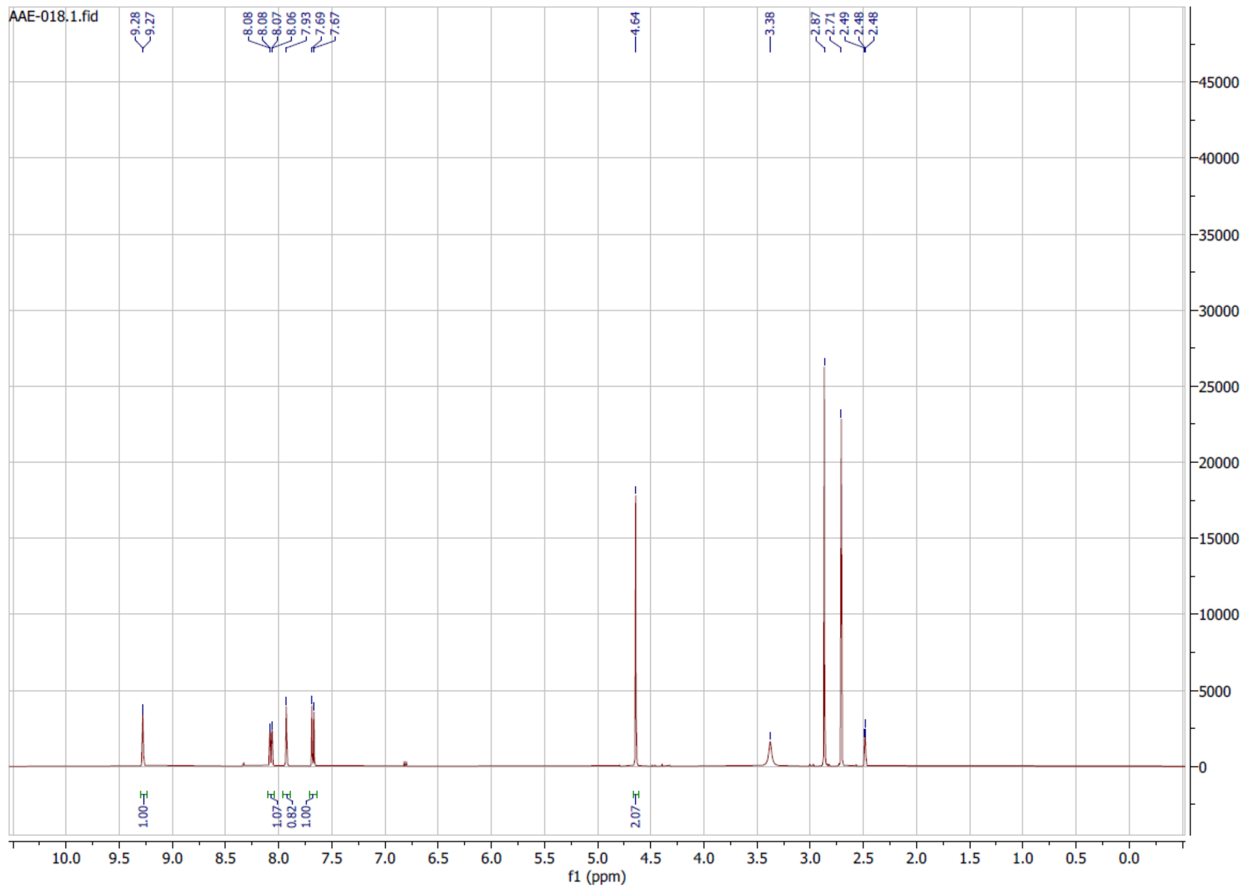

Figure S33. ^{1}H NMR for 2-chloro-*N*-(5-nitro-1,2-benzothiazol-3-yl)acetamide (**11**).

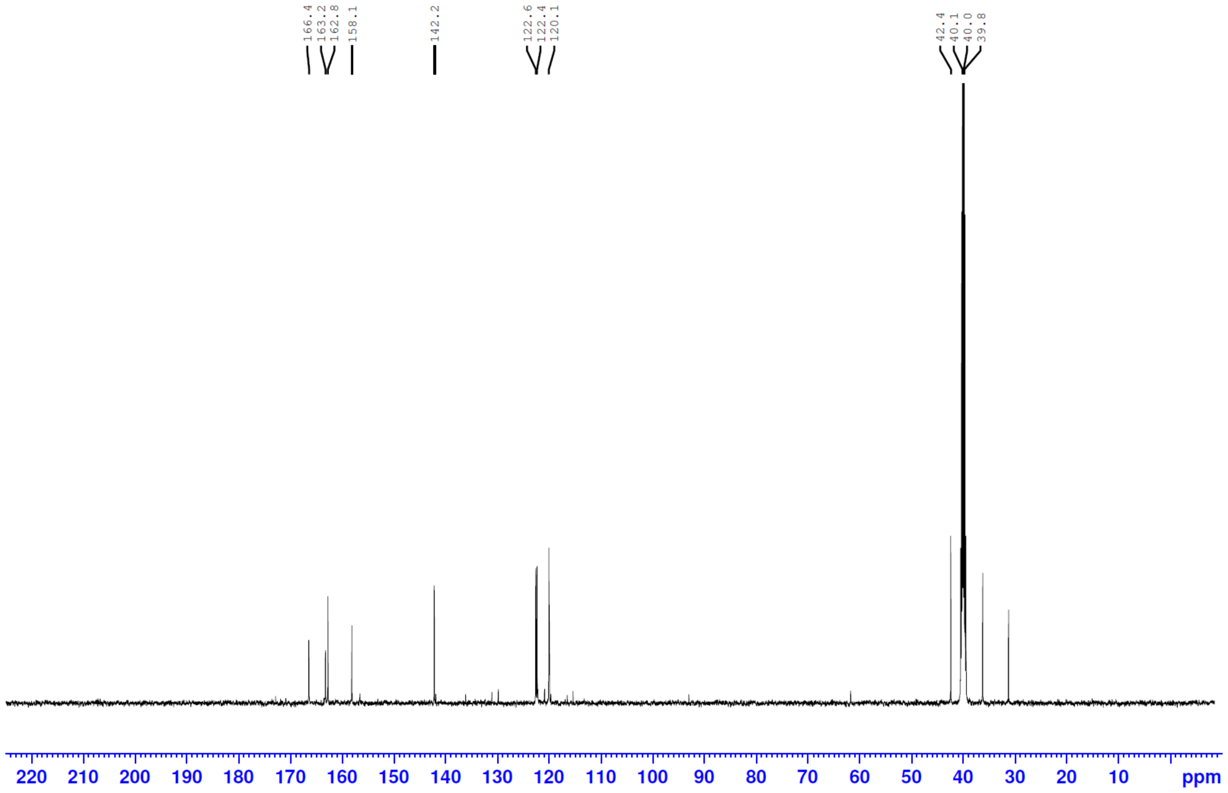

Figure S34. ^{13}C NMR for 2-chloro-*N*-(5-nitro-1,2-benzothiazol-3-yl)acetamide (**11**).

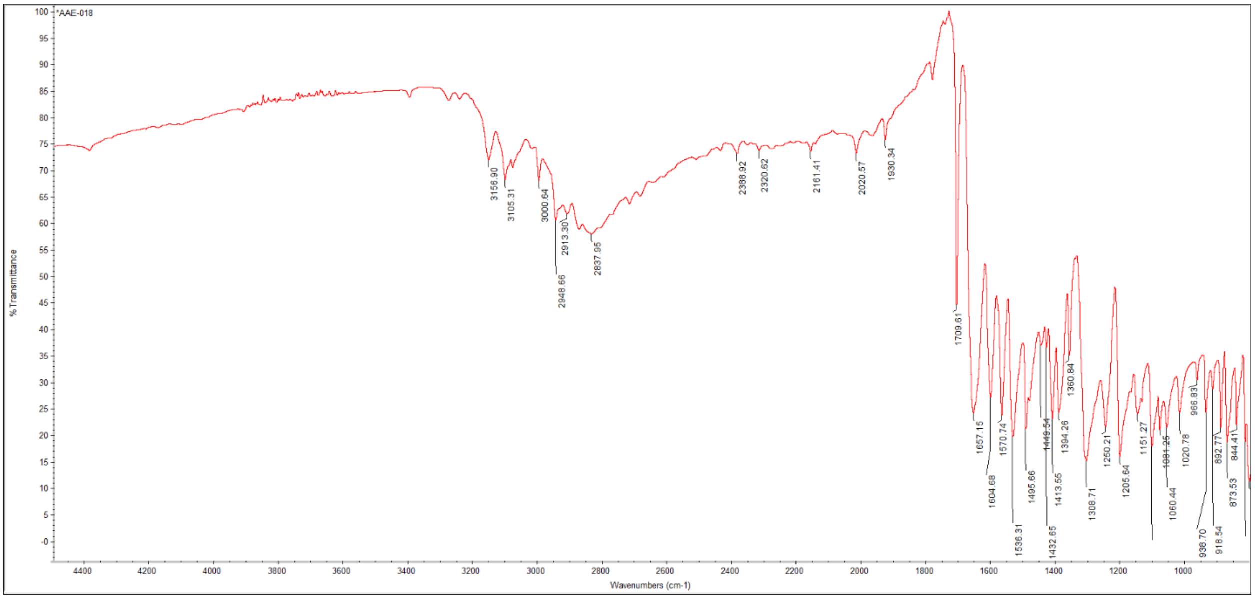

Figure S35. IR (solid) v/cm^{-1} for 2-chloro-*N*-(5-nitro-1,2-benzothiazol-3-yl)acetamide (**11**).

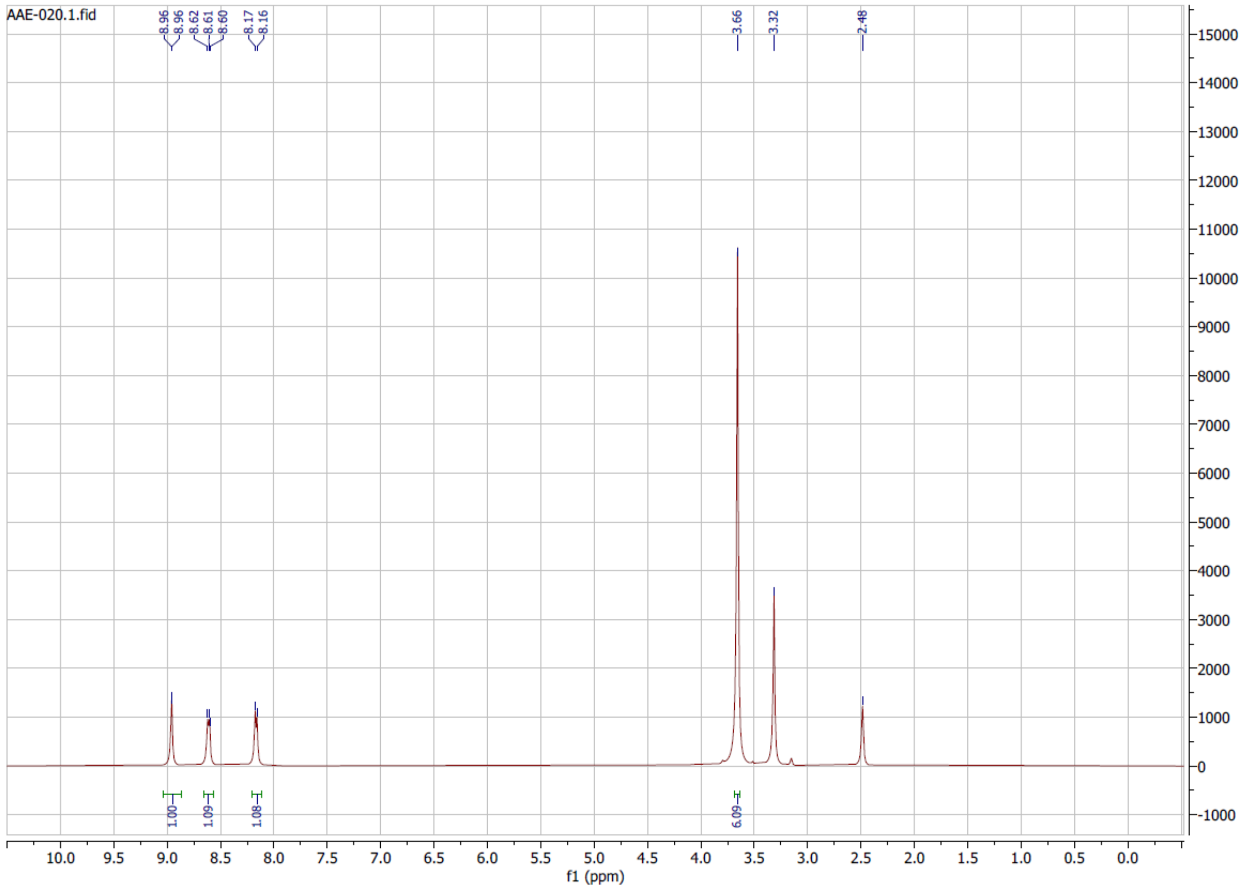

Figure S36. ^{1}H NMR for *N*-methanesulfonyl-*N*-(5-nitro-1,2-benzothiazol-3-yl)methanesulfonamide (**12**).

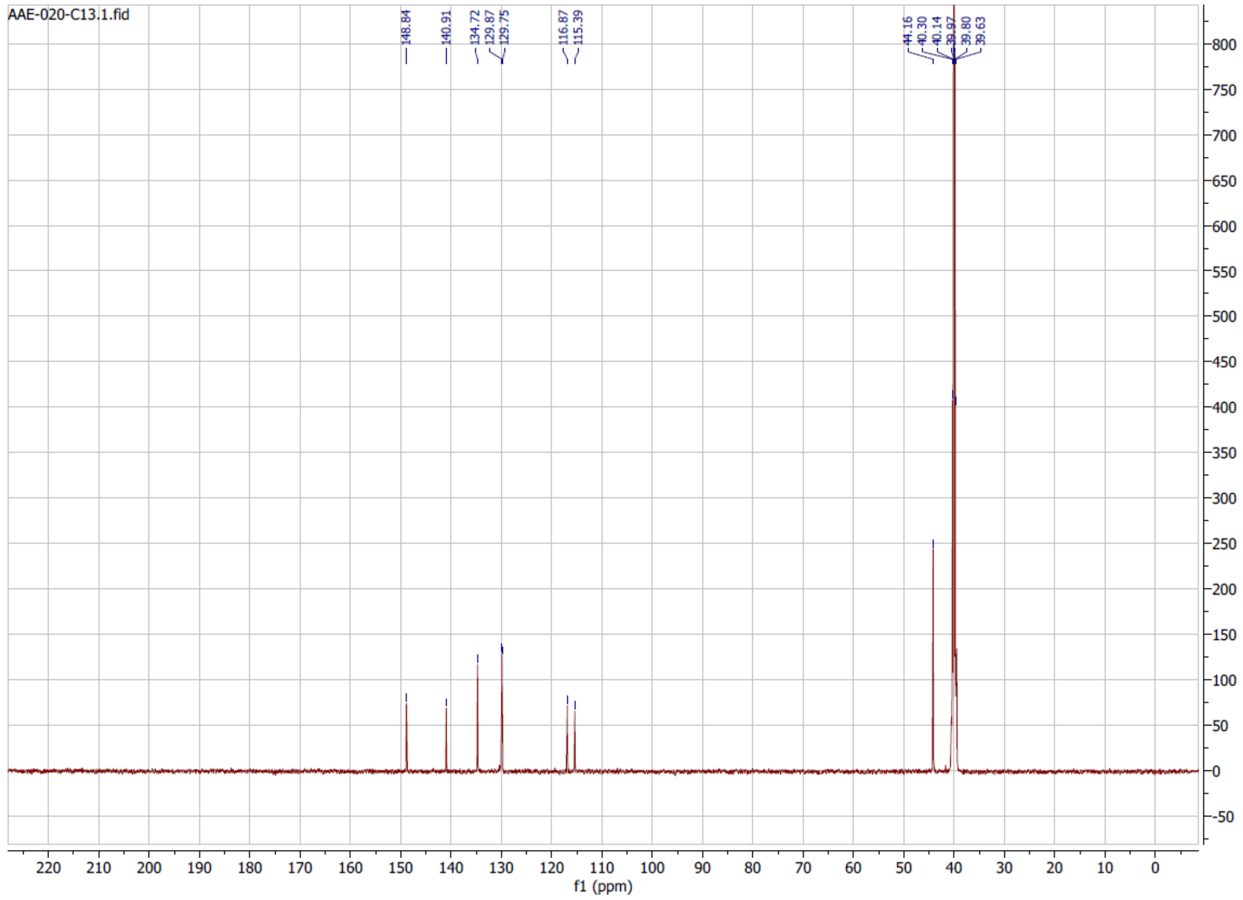

Figure S37. ^{13}C NMR for *N*-methanesulfonyl-*N*-(5-nitro-1,2-benzothiazol-3-yl)methanesulfonamide (**12**).

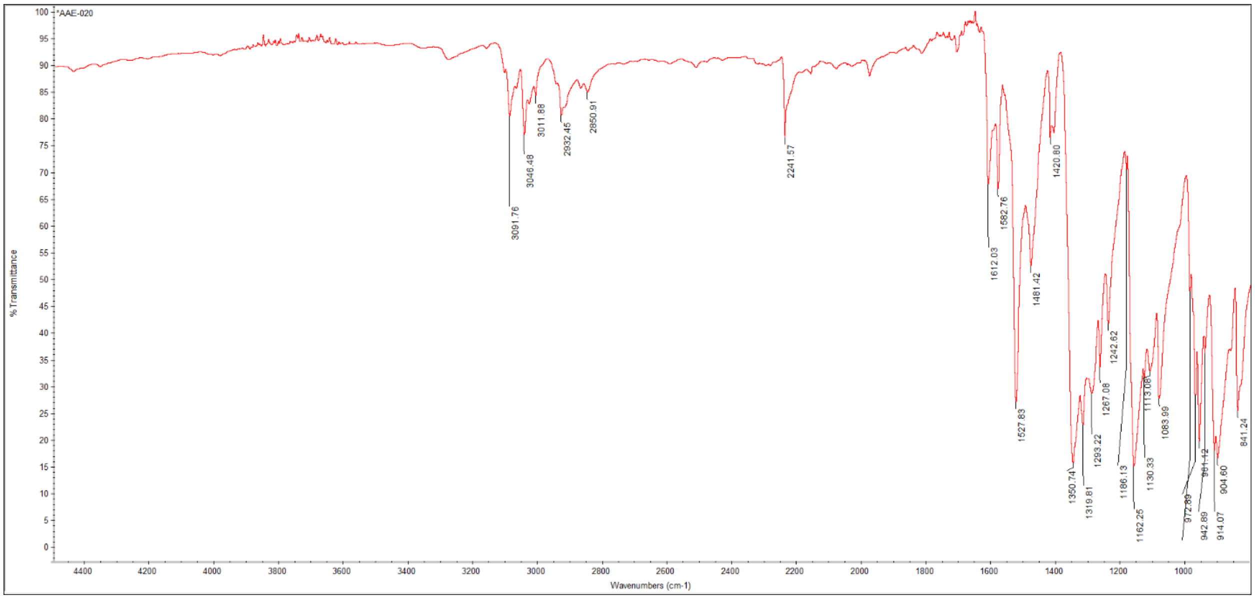

Figure S38. IR (solid) *v*/cm^{-1} for *N*-methanesulfonyl-*N*-(5-nitro-1,2-benzothiazol-3-yl)methanesulfonamide (**12**).

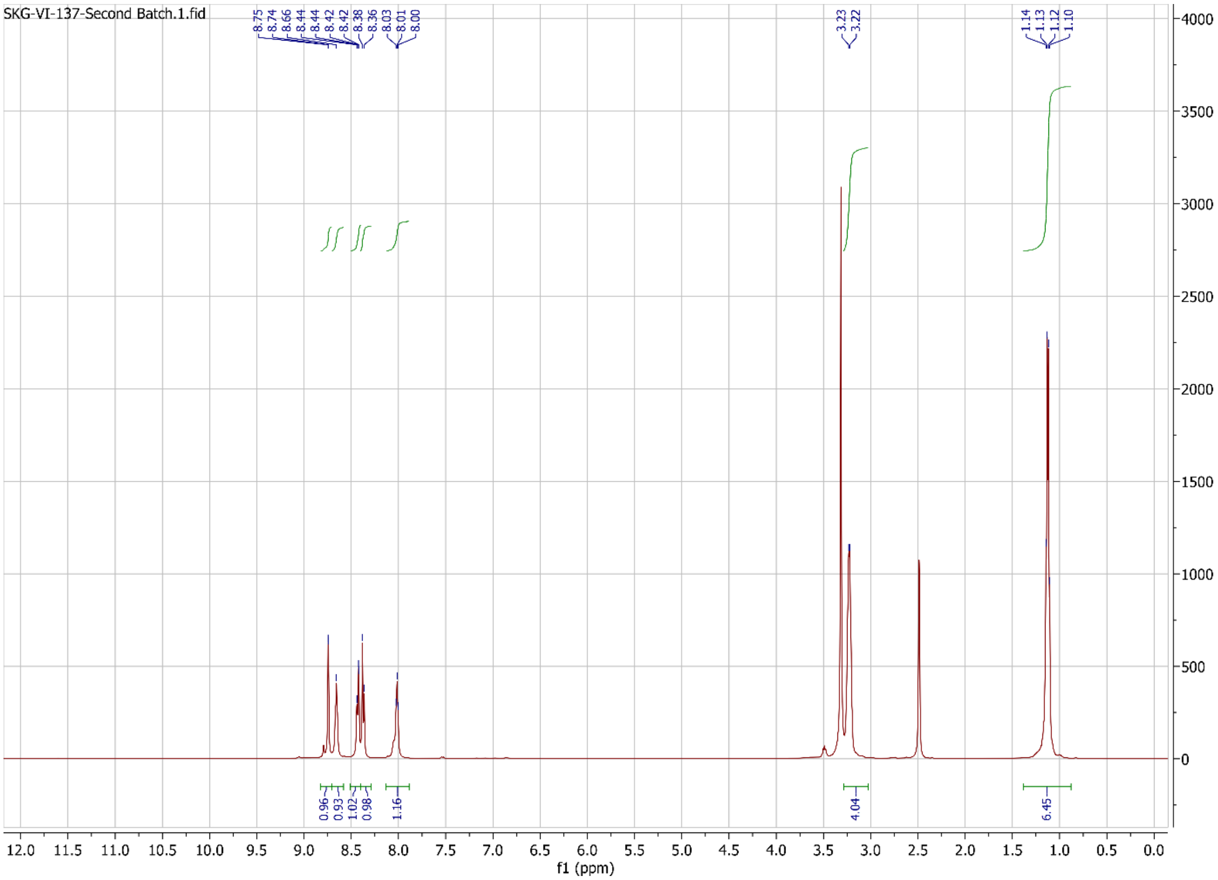

Figure S39. ^{1}H NMR for *N*-ethyl-1-[(ethylcarbamoyl)(5-nitro-1,2-benzothiazol-3-yl)amino]formamide (**13**).

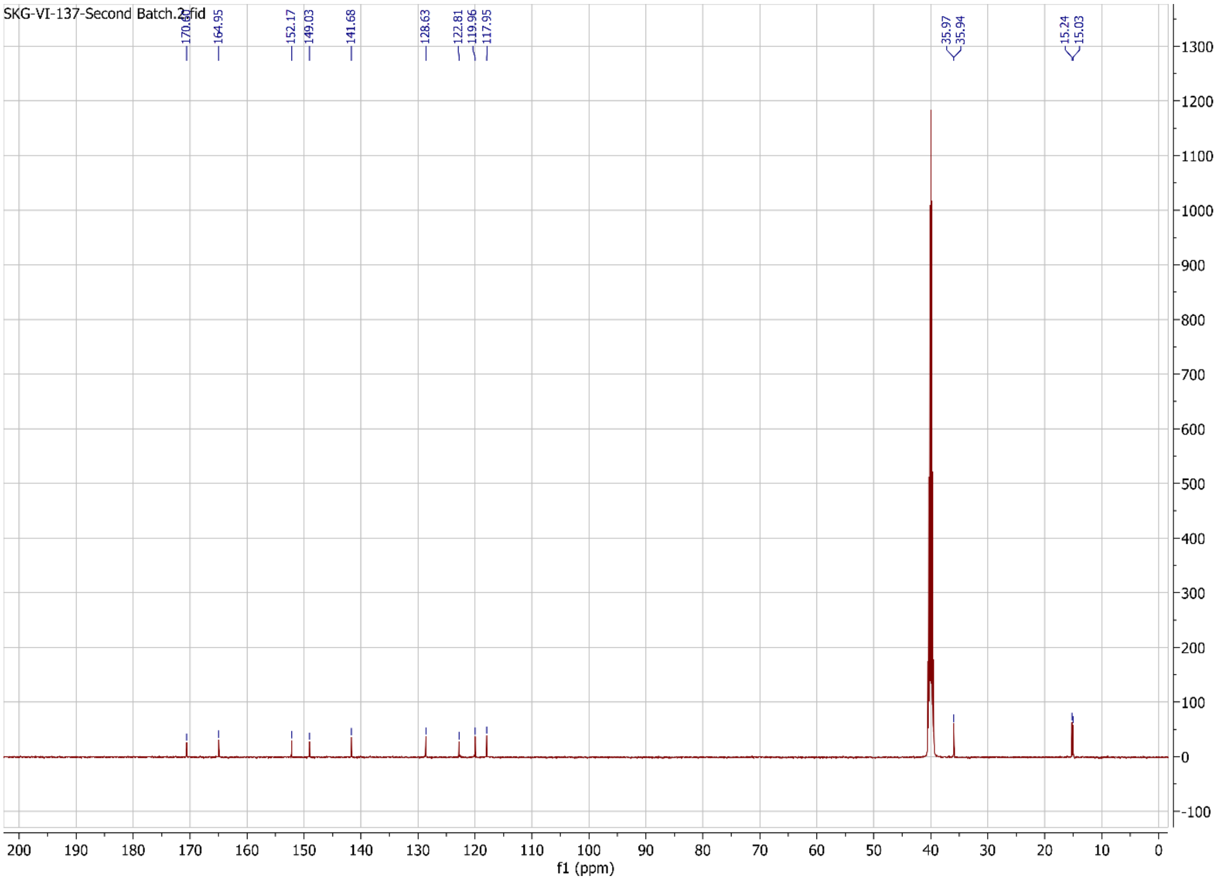

Figure S40. ^{13}C NMR for *N*-ethyl-1-[(ethylcarbamoyl)(5-nitro-1,2-benzothiazol-3-yl)amino]formamide (**13**).

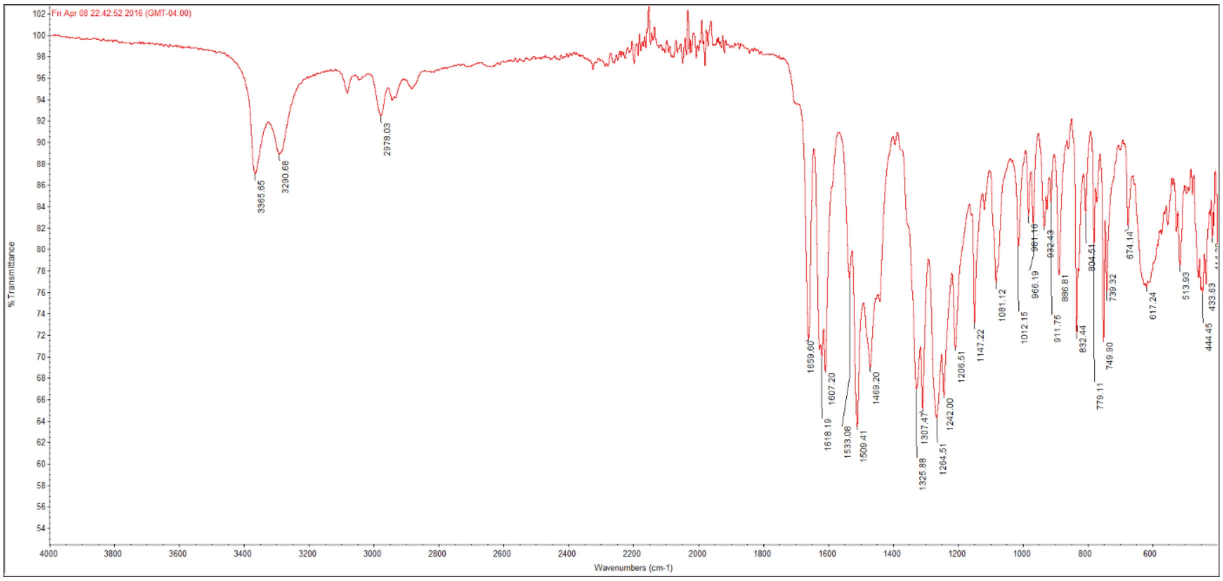

Figure S41. IR (solid) ν/cm^{-1} for *N*-ethyl-1-[(ethylcarbamoyl)(5-nitro-1,2-benzothiazol-3-yl)amino]formamide (**13**).

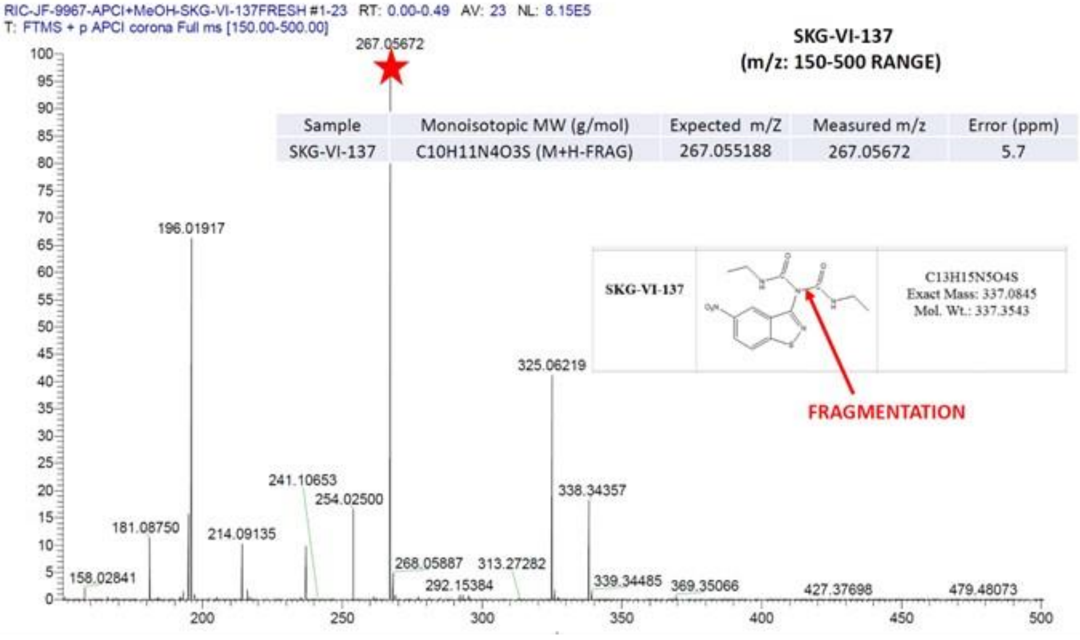

Figure S42. Mass spectrum (ionization in APCI) for *N*-ethyl-1-[(ethylcarbamoyl)(5-nitro-1,2-benzothiazol-3-yl)amino]formamide (**13**).

References

1. Fernandez-Escamilla, A. M.; Rousseau, F.; Schymkowitz, J.; Serrano, L., Prediction of sequence-dependent and mutational effects on the aggregation of peptides and proteins. *Nat. Biotechnol.* **2004,** *22* (10), 1302-6.

2. Rousseau, F.; Schymkowitz, J.; Serrano, L., Protein aggregation and amyloidosis: confusion of the kinds? *Curr. Opin. Struct. Biol.* **2006,** *16* (1), 118-26.

3. Linding, R.; Schymkowitz, J.; Rousseau, F.; Diella, F.; Serrano, L., A comparative study of the relationship between protein structure and beta-aggregation in globular and intrinsically disordered proteins. *J. Mol. Biol.* **2004,** *342* (1), 345-53.

4. Klimtchuk, E. S.; Prokaeva, T.; Frame, N. M.; Abdullahi, H. A.; Spencer, B.; Dasari, S.; Cui, H.; Berk, J. L.; Kurtin, P. J.; Connors, L. H.; Gursky, O., Unusual duplication mutation in a surface loop of human transthyretin leads to an aggressive drug-resistant amyloid disease.**,** *Proc. Natl. Acad. Sci. U.S.A.* **2018**, *115* (28), E6428-E6436.

5. Porat, Y.; Abramowitz, A.; Gazit, E., Inhibition of amyloid fibril formation by polyphenols: structural similarity and aromatic interactions as a common inhibition mechanism. *Chem. Biol. Drug. Des.* **2006,** *67* (1), 27-37.

6. Jiang, P.; Li, W.; Shea, J. E.; Mu, Y., Resveratrol inhibits the formation of multiple-layered beta-sheet oligomers of the human islet amyloid polypeptide segment 22-27. *Biophys. J.* **2011,** *100* (6), 1550-8.
